# Selective Esterification of Phosphonic Acids †

**DOI:** 10.3390/molecules26185637

**Published:** 2021-09-17

**Authors:** Damian Trzepizur, Anna Brodzka, Dominik Koszelewski, Ryszard Ostaszewski

**Affiliations:** Institute of Organic Chemistry, Polish Academy of Science, Kasprzaka 44/52, 01-224 Warsaw, Poland; dtrzepizur@icho.edu.pl (D.T.); awoltanska@icho.edu.pl (A.B.); dkoszelewski@icho.edu.pl (D.K.)

**Keywords:** phosphonic acids, alkyl orthoesters, selective esterification, temperature effect

## Abstract

Here, we report straightforward and selective synthetic procedures for mono- and diesterification of phosphonic acids. A series of alkoxy group donors were studied and triethyl orthoacetate was found to be the best reagent as well as a solvent for the performed transformations. An important temperature effect on the reaction course was discovered. Depending on the reaction temperature, mono- or diethyl esters of phosphonic acid were obtained exclusively with decent yields. The substrate scope of the proposed methodology was verified on aromatic as well as aliphatic phosphonic acids. The designed method can be successfully applied for small- and large-scale experiments without significant loss of selectivity or reaction yield. Several devoted experiments were performed to give insight into the reaction mechanism. At 30 °C, monoesters are formed via an intermediate (1,1-diethoxyethyl ester of phosphonic acid). At higher temperatures, similar intermediate forms give diesters or stable and detectable pyrophosphonates which were also consumed to give diesters. ^31^P NMR spectroscopy was used to assign the structure of pyrophosphonate as well as to monitor the reaction course. No need for additional reagents and good accessibility and straightforward purification are the important aspects of the developed protocols.

## 1. Introduction

Organophosphonates are a relatively diverse and common group of compounds, which feature the presence of a stable C–P bond. In nature, they are present in microorganisms as well as in metazoans. Furthermore, they constitute a considerable part of phosphorus compounds found in oceans [1]. It is also considered that organophosphonates could play an important role at the early stages of the development of life on Earth when the oxygen concentration in the atmosphere was negligible [2]. Currently, phosphonates have a large number of applications in agriculture [3] and medicine [4,5], as well as markers of chemical warfare agents (CWAs), and many others [6].

The synthesis of organophosphorus esters from related phosphites in reaction with organic halides (Michaelis–Arbuzov reaction) is well-established chemistry [7,8]. The corresponding transformations leading to phosphonates from hydrogenphosphonates (Michaelis–Becker [9,10], Atherton–Todd [11,12] reactions), phosphonic dichlorides [13,14] and phosphonochloridates [15,16] (alcoholysis) or even simpler substrates such as phosphoryl chloride [17] are also broadly employed as well. However, the abovementioned methods are connected with the generation of corrosive and hazardous halide waste [18]. Furthermore, most of the described methods are not suitable for application in a standard laboratory or require harsh reaction conditions, toxic solvents [10] and laborious preparation [19,20].

An alternative route for target compounds is direct esterification or transesterification of the phosphonic acids. The esterification can be achieved in the presence of chlorinated silica gel [21], by exploitation of Garegg–Samuelsson conditions [22], dicyclohexylcarbodiimide mediated esterification [23] or O-alkylation [24], whereas alkyl hydrogen phosphonates can be received from the diesters by partial hydrolysis [20,25] or via transesterification with silyl halides and selective cleavage by protic solvents [26]. Monoesters can also be obtained directly from phosphonic acids upon selective monoesterification. For successful syntheses, phenylarsonic acid [27,28], carbodiimides [29,30], solid-phase synthesis [31] and ionic liquids [24] were used. Unfortunately, none of the mentioned methods can be performed under mild conditions, with high yields and good selectivity towards monoesters.

**Scheme 1:**
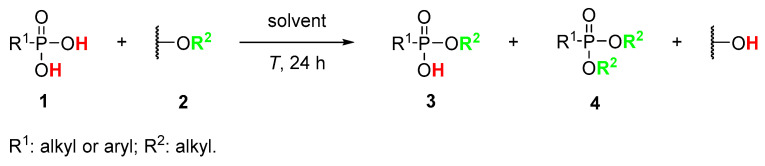
The general concept of esterification of phosphonic acids.

Recently, we have found that esterification of carboxylic acid proceeds efficiently upon the application of orthoesters [32], carbonates [33], orthocarbonates [34], acetals and ketals [35] as alkoxy group donors. The studies on the reaction mechanism proved that dialkoxy carboxylate is a key intermediate that is subsequently and irreversibly transformed into ethyl acetate which is a driving force for the reaction [32]. Since phosphonic acids are the isosteres of carboxylic acids, the same principle is proposed for the esterification of phosphonic acids (**1**) using corresponding alkoxy group donors (**2**). The esterification of phosphonic acids is more complicated since during the reaction two possible products, mono- (**3**) and diesters (**4**), can be formed simultaneously (Scheme 1). The straightforward synthesis of diester **4** is obvious [36,37,38,39] while the selective formation of monoesters is very tricky. The application of alkoxy group donors in monoesterification of phosphonic acids has never been reported before.

## 2. Results and Discussion

**Figure 1:**
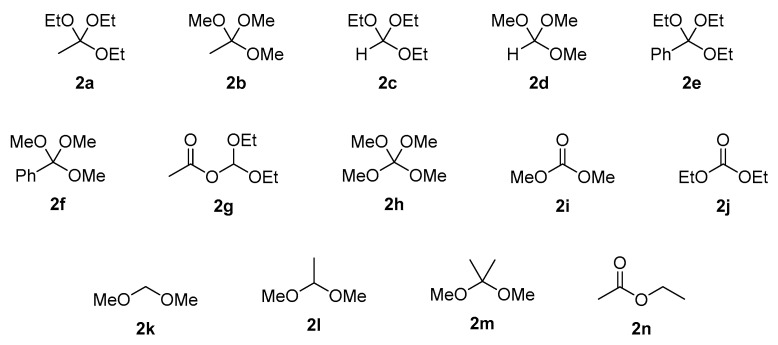
Structures of alkoxy group donors used in experiments.

For preliminary experiments, both butylphosphonic (**1a**) and phenylphosphonic (**1b**) acids were selected as model substrates. Initially, various potential alkoxy group donors were investigated in the monoesterification reaction (Table 1). Their structures are presented in Figure 1. The reactions were conducted in methyl *tert*-butyl ether (MTBE) at 30 °C for 24 h. The conversion of substrates and selectivity toward products were determined by ^1^H and ^31^P NMR analyses.

The application of triethyl orthoacetate (**2a**) as an ethoxy group donor resulting in high conversion, yielding 84% of **3a** and 83% of **3b** (Table 1, entries 1 and 15). In both cases, excellent selectivity towards monoesters **3a** and **3b** was observed (yields of diesters **4a** and **4b** were below 3%). Trimethyl orthoacetate (**2b**) was found to be a less efficient donor of an alkoxy group, the conversion of acid **1a** reached 25% and that of acid **2b** was 50% (Table 1, entries 2 and 16). In the case of triethyl orthoformate (**2c**), trimethyl orthoformate (**2d**), triethyl orthobenzoate (**2e**) and trimethyl orthobenzoate (**2f**), no substrate conversion was observed (Table 1, entries 3–6, 17–20). Along with **2a**, diethoxymethyl acetate (**2g**) was also found to be a selective ethoxy group donor. In reaction with butylphosphonic acid (**1a**), the conversion of substrate was 39% (Table 1, entry 7) and for phenylphosphonic acid (**1b**) it was 89% (Table 1, entry 21). The studied transformation was very selective towards monoesters **3**. The application of donor **2g** provides a higher conversion of phenylphosphonic acid with respect to butylphosphonic acid. Tetramethyl orthocarbonate (**2h**) was also active in the esterification reaction, providing ester **3a** in 23% and ester **3b** in 44% yields, respectively (Table 1, entries 8, 22). When other donors such as dimethyl carbonate (**2i**), diethyl carbonate (**2j**), formaldehyde dimethyl acetal (**2k**), acetaldehyde dimethyl acetal (**2l**), 2,2-dimethoxypropane (**2m**) and ethyl acetate (**2n**) were applied, no reaction occurred (Table 1, entries 9–14 and 23–28).

In the next step of our research, we tested the impact of the reaction medium using **2a** as a donor (Table 2), because the preliminary used MTBE was selected arbitrarily. For butylphosphonic acid **1a**, when the reaction was conducted in tetrahydrofuran, a slight drop in reaction yield was observed with respect to MTBE while in the case of phenylphosphonic acid **1b**, the result was similar (Table 2, entries 1 and 2). In dichloromethane, conversion was high for acid **1a** and almost quantitative for acid **1b**, with good selectivity towards monoesters **3a** and **3b** (Table 2, entry 3). In ethyl acetate and acetonitrile, the conversions were lower for both substrates (Table 2, entries 4 and 5). Acetone was found to be an inappropriate solvent with very low substrate conversion (Table 2, entry 6). The reaction proceeds effectively in toluene (Table 2, entry 7) but the highest conversion (95%) was observed when **2a** was used as a reagent and solvent (Table 2, entry 8). No reaction progress was observed in experiments conducted in dimethyl sulfoxide and dimethylformamide (Table 2, entries 9 and 10).

The analysis of data from Table 2 concludes that the best medium for the studied transformation is triethyl orthoacetate **2a** when used in large excess with respect to substrate (over 5 equiv.). After selection of the best solvent for reaction, we studied the impact of temperature on the esterification (Table 3). Reactions of butylphosphonic acid **1a** with orthoacetate **2a** were performed at temperatures varying from 30°C to 100 °C. The increase in the reaction temperature from 30 to 40 °C resulted in a higher conversion of the substrate (over 99%) and slightly influenced the yield of the product (Table 3, entries 1 and 2). At elevated temperatures, the conversion of the substrate became quantitative (Table 3, entries 2–8) and the selectivity towards diester increased gradually. The amount of monoester **3a** in the reaction mixture dropped to 1% at 90 °C (Table 3, entry 7). At 100 °C, partial decomposition of the product was observed (Table 3, entry 8).

In conclusion, the considered data indicate that at higher temperatures, diester **4a** is formed as a single product (Table 3, entries 7 and 8). The best conditions for diesterification of butylphosphonic acid **1a** involved reaction in excess of orthoester **2a** at 90 °C. An analogous esterification performed at 30 °C led to the selective formation of monoethyl butylphosphonate (**3a**).

With the optimized reaction conditions in hand, we proceeded to assess the generality and scope of the developed procedures. Table 4 summarizes the results of the preparation of several mono- and diesters from various phosphonic acids. It should be noted that for the experiments performed on a laboratory scale, the favourable amount of orthoacetate **2a** was 15 equiv. for monoesterification and 30 equiv. for diesterification.

The preparative esterification of model butylphosphonic acid (**1a**) proceeded efficiently and monoester **3a** and diester **4a** were obtained in 98% yields (Table 4, entries 1 and 2). For effective monoesterification of benzylphosphonic acid, a longer reaction time, 48 h, was required and respective monoester **3b** was obtained in a 76% isolated yield (Table 4, entry 3). Diester **4b** was obtained in an almost quantitative 98% yield (Table 4, entry 4). Monoesterification proceeded smoothly for the other alkylphosphonic acids (Table 4, entries 5, 7, 9 and 11) and yields of monoesters exceeded 83%. The selective monoesterification of aryl and benzylphosphonic acids was also successful. Presumably due to poor solubility of 4-hydroxybenzylphosphonic acid (**1i**) and [1,4-phenylenebis(methylene)]-bis(phosphonic acid) (**1m**), no product formation (**3i** and **3m**) was observed (Table 4, entries 17, 25).

Exhaustive esterification of other aliphatic acids in established conditions gave respective diesters **4c**, **4e** and **4f** in 89–97% yields (Table 4, entries 6, 10, and 12). For vinylphosphonic acid (**2d**), product **4d** was not received due to its rapid polymerization at elevated temperatures (Table 4, entry 8). Diesterification for arylphosphonic acids **1g** and **1h** proceeded smoothly and esters **4g** and **4h** were isolated in quantitative yield (Table 4, entries 14–16). Substituted derivatives of benzylphosphonic acid **1i**–**l** underwent double esterification with good to excellent yields (Table 4, entries 18, 20, 22 and 24). For 24 h, at 90 °C, reaction with substrate **1m** (*p*-xylene diphosphonic acid) proceeded with low conversion. Therefore, to accelerate the transformation, the temperature was raised to 145 °C, and the reaction mixture was refluxed for 24 h which led to product **4m** with quantitative yield (Table 4, entry 26).

It is important to note that using a single reagent—triethyl orthoacetate—two types of products, mono- (**3**) and diethylesters (**4**), were obtained under mild reaction conditions in a reasonable time without the use of any toxic or polluting reagents. In almost all cases, products were obtained as single compounds upon evaporation of the reaction mixture to dryness. The developed procedure partially fulfills green chemistry principles and can be applied to a fair range of phosphonic acids.

**Figure 2:**
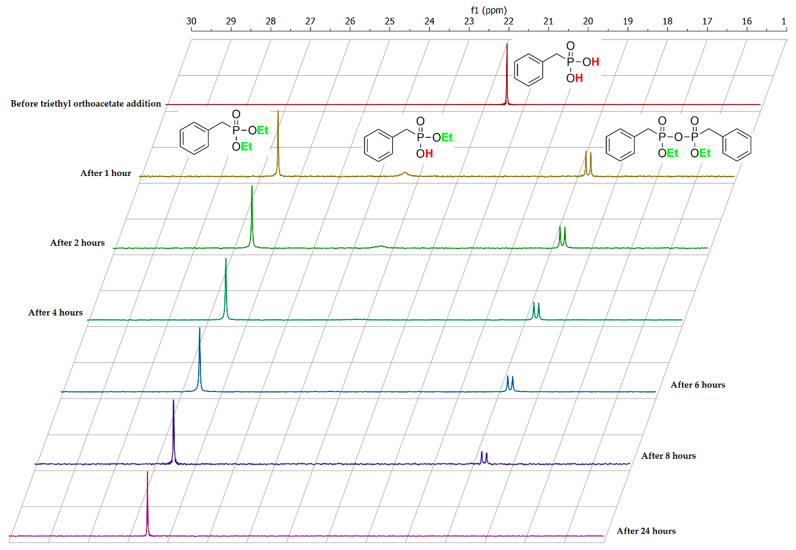
^31^P NMR spectrum of substrate **1b** stacked with spectra of reaction mixture recorded during the course of the reaction.

**Figure 3:**
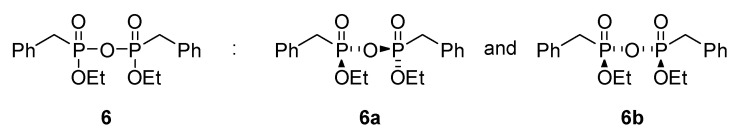
Structures of diastereomers **6a** and **6b** of diethyl dibenzylpyrophosphonate **6** which was identified in the reaction mixture.

The gain insight into the reaction mechanism, additional experiments were performed on one of the model substrates—benzylphosphonic acid **1b**—with selected donor **2a** at 30° and 75 °C. Close inspection of ^31^P NMR spectra of crude **3b** product obtained in reaction at 30 °C did not show any additional signals from possible reaction intermediates (see Supplementary Materials Section S2.3). However, in the reaction performed at the higher temperature, an interesting phenomenon was observed. Formally, before the reaction had started, the spectrum of the substrate **1b** was measured. It is at the top of Figure 2 and shows a single peak at 21.3 ppm. After the addition of triethyl orthoacetate and heating the reaction mixture for 1 h at 75 °C, the NMR spectra changed substantially. The signal associated with substrate **1b** diminished almost to zero and four new signals appeared on the spectra. The main peak at 26.5 ppm comes from diester **4b.** The amount of product **4b** increased rapidly during this first hour and reached a maximum after 24 h. The small and broad signal at 23.3 ppm represents monoester **3b**. The most significant peak of the **3b** product appeared in the first spectrum (after an hour). Further measurements demonstrated that the amount of **3b** dropped close to zero after 8 h. Surprisingly, two new signals at 18.65 and 18.77 ppm of similar intensity appeared. Close inspection of literature data [40] allowed us to assign those two signals to diastereomers **6a** and **6b** of pyrophosphonate **6** (Figure 3). The appearance of compound **6** as a reaction intermediate is a valuable clue to the reaction mechanism.

**Figure 4:**
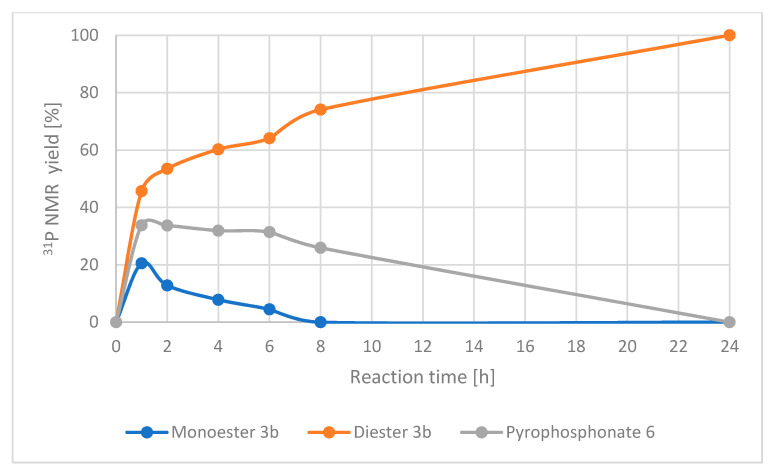
Changes in concentration of products with time.

The calculated ^31^P NMR yield of pyrophosphonate **6** reached 34% after 1 h and its amount remained at a similar level for the next 5 h and then slowly decreased to zero. The detailed data from the ^31^P NMR spectra analysis are summarized in Table S1 (SI). To sum up, the NMR spectra recorded during the reaction course showed that the amount of diester **4b** was continuously increasing with time while amounts of **3b** and **6** reached a kind of plateau and then were diminished to zero after 24 h. As mentioned above, at a lower reaction temperature (30 °C) signals from pyrophosphonate **6** were not observed and a higher reaction temperature was required to transform monoester **3b** into pyrophosphonate **6** and finally into diester **4b**. The graphical representation of the results described above is shown in Figure 4.

**Scheme 2:**
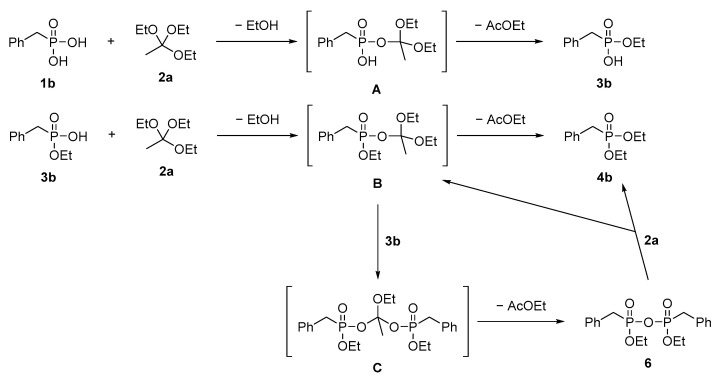
A plausible mechanism for esterification of phosphonic acid **1b** with orthoester **2a**.

The analysis of obtained data provided some insight into the reaction mechanism (Scheme 2). The first step is the reaction between triethyl orthoacetate (**2a**) and benzylphosphonic acid (**1b**) which leads to the formation of an unstable intermediate **A**. It readily decomposes to monoester **3b** by releasing one molecule of ethanol and ethyl acetate in a two-step process, at room temperature. The intermediate **A** can be formed only from selected substrates used as alkoxy group donors, depicted in Figure 1. This mechanism allowed us to explain why only monophosphonates were formed from orthoesters **2a** and **2b**, diethoxymethyl acetate **2g** and tetramethyl orthocarbonate **2h**. At higher temperatures, monoester **3b** reacts with a second molecule of triethyl orthoacetate to give intermediate **B**, similar to **A**. This intermediate can react with the second molecule of monoester **3b** to give compound **C** which is readily transformed into pyrophosphonate **6** and its structure was ambiguously assigned by kinetic experiments. Compound **6** reacts with the third molecule of orthoester **2a** to give the final product diester **4b** and intermediate **B**. According to this mechanism, for the formation of one molecule of diester **4**, three molecules of orthoester **2a** are required. This readily explains why the larger amount of triethyl orthoacetate favors double esterification with respect to monoesterification.

## 3. Materials and Methods

### 3.1. General

Commercially available reagents were used without additional purification. Phosphonic acids were purchased or prepared according to reported literature procedures. The water and hexane mixtures were previously distilled. Other solvents (analytical grade) were used without extra drying and purification. Reactions were performed in dry laboratory glassware under an air atmosphere (otherwise noted) using magnetic stirrers. Solvents and volatile reagents were evaporated under reduced pressure. Merck (Darmstadt, Germany) silica gel plates 60 F254 were used for TLC analysis.

The majority of obtained compounds did not need additional purification after reaction workup. Those requiring isolation were separated via distillation under reduced pressure on Kugelrohr apparatus. Melting points were measured on a Boetius hot plate microscope (Nagema, Dresden, Germany). The phosphorous nuclear magnetic resonance (^31^P{1H} NMR) spectra of analyzed compounds, dissolved in DMSO-d_6_, D_2_O or CDCl_3_, were recorded with a Bruker spectrometer (162.0 MHz) at 30 °C and using 85% H_3_PO_4_ as external calibration. ^1^H and ^13^C NMR spectra were also recorded in DMSO-d_6_, D_2_O or CDCl_3_ (30 °C) on the same Bruker spectrometer at 400 and 101 MHz, respectively. Chemical shifts were reported in ppm and referred to residual deuterated solvent signal; coupling constants (*J*) were noted in Hz. Low-resolution mass spectra were recorded on the API365i API 3000 spectrometer, and the ESI technique was used to analyte ionization. High-resolution mass spectra were recorded on the Maldi SYNAPT G2-S HDMS (Waters, Milford, MA, USA) apparatus with a QqTOF analyzer.

### 3.2. General Procedure for Esterification of Phosphonic Acid Using Various Alkoxy Group Donors

To a solution of butylphosphonic acid **1a** (1 equiv., 7 mg, 0.05 mmol) or benzylphosphonic acid **1b** (1 equiv., 9 mg, 0.05 mmol) in MTBE (1 mL), the respective alkoxy group donor **2** (3 equiv.) was added and stirred at 30 °C for 24 h. The solvent and other volatile compounds were evaporated under reduced pressure. The crude product was dissolved in 0.6 mL DMSO-d_6_ and the ^31^P NMR spectrum was recorded. Subsequently, the conversion of substrate **1** and yields of products **3** and **4** were determined on the basis of relative peak integrals.

### 3.3. General Procedure for Esterification of Phosphonic Acids Using Triethyl Orthoacetate in Different Solvents

To a solution of butylphosphonic acid **1a** (1 equiv., 7 mg, 0.05 mmol) or benzylphosphonic acid **1b** (1 equiv., 9 mg, 0.05 mmol), 1 mL of respective solvent triethyl orthoacetate **2a** (3 equiv., 25 mg, 0.15 mmol) was added and stirred at 30 °C for 24 h. The solvent and other volatile substances were evaporated under reduced pressure. Obtained crude was dissolved in 0.6 mL DMSO-d_6_ and the spectrum was recorded. The conversion of substrate **1** and yield of products **3** and **4** were determined based on ^31^P NMR spectrum analysis.

### 3.4. The Influence of Temperature on the Selectivity of Esterification Reaction of Butylphosphonic acid with Triethyl Orthoacetate

Butylphosphonic **1a** (1 equiv., 14 mg, 0.1 mmol)) in 1 mL orthoacetate **2a** was heated at the temperature indicated in Table 3 for 24 h. After that, the reaction mixture was cooled to room temperature and the excess **2a**, as well as volatile byproducts, was evaporated to dryness. Received crude was dissolved in DMSO-d_6_ and the prepared sample was used for the ^31^P NMR measurements, based on which the conversion of **1a** and product **3a** and **4a** yields were determined.

### 3.5. General Procedure for Selective Monoesterification of Phosphonic Acids ***1*** Using Triethyl Orthoacetate ***2a*** Leading to Ethyl Hydrogen Phosphonates ***3***

Phosphonic acid **1** (1 mmol) and triethyl orthoacetate (15 mmol, 2.75 mL) were stirred at 30 °C and the substrate conversion was monitored by ^31^P NMR. After the competition of the reaction, the orthoacetate excess and volatile by-product were evaporated off under vacuum. In several cases, the remaining product required no further purification. If necessary, the extraction procedure proposed by Campbell [41] was applied: the crude was diluted with diethyl ether (10 mL) and extracted twice with NaHCO_3_ (5 mL, 5% *w*/*v* solution in water). The aqueous fractions were combined and washed with two portions of ethyl ether (2 × 5 mL). The aqueous phase was acidified with hydrogen chloride solution (1 M) to reach pH 2 and extracted three times with ethyl acetate (3 × 5 mL). Combined ethyl acetate fractions were dried over magnesium sulfate and pure products were received after solvent evaporation to dryness.

### 3.6. General Procedure for Esterification of Phosphonic Acids ***1*** Using Triethyl Orthoacetate Leading to Diethyl Phosphonic Esters ***4***

Phosphonic acid **1** (1 mmol) and triethyl orthoacetate (5.5 mL, 30 mmol) were stirred at 90 °C (or, for sparingly soluble substrates, at a higher temperature) for 24 h under a reflux condenser. The progress of the reaction was monitored by ^31^P NMR. The excess of orthoester was evaporated under reduced pressure and the product—dialkyl phosphonate **4**—was received in pure form. In some cases, the purity of diester **4** was not satisfactory and additional purification by bulb-to-bulb vacuum distillation with Kugelrohr apparatus or column chromatography was performed.

#### 3.6.1. Ethyl Hydrogen Butylphosphonate (**3a**)

The synthesis of **3a** was conducted according to Section 3.5.

Yield 98%; ^1^H NMR data in accordance with the previous report [42]; colorless oil; ^1^H NMR (400 MHz, DMSO-d_6_) 3.90 (dq, *J*_H-P_ = 8.0, *J*_H-H_ = 7.0 Hz, 2H), 1.63–1.50 (m, 2H), 1.50–1.29 (m, 4H), 1.20 (t, *J*_H-H_ = 7.1 Hz, 3H), 0.86 (t, *J*_H-H_ = 7.2 Hz, 3H); ^13^C NMR (101 MHz, DMSO-d_6_) δ 59.9 (d, *J*_C-P_ = 6.0 Hz), 25.5 (d, *J*_C-P_ = 137.6 Hz), 24.5 (d, *J*_C-P_ = 4.9 Hz), 23.1 (d, *J*_C-P_ = 16.2 Hz), 16.4 (d, *J*_C-P_ = 5.9 Hz), 13.6; ^31^P NMR (162 MHz, DMSO-d_6_) δ 29.0.

#### 3.6.2. Ethyl Hydrogen Benzylphosphonate (**3b**)

The synthesis of **3b** was conducted according to Section 3.5, with the only difference being that the reaction time was extended to 48 h. Yield: 76%; ^1^H NMR and ^31^P NMR spectra were in accordance with previous literature reports [43]; pale yellow oil; ^1^H NMR (400 MHz, DMSO-d_6_) δ 7.51–7.15 (m, 5H; broad -OH, 1H), 3.93–3.84 (m, 2H), 3.06 (d, *J*_H-P_ = 21.4 Hz, 2H), 1.15 (t, *J*_H-H_ = 7.1 Hz, 3H); ^13^C NMR (101 MHz, DMSO-d_6_) δ 133.4 (d, *J*_C-P_ = 8.9 Hz), 129.7 (d, *J*_C-P_ = 6.4 Hz), 128.1 (d, *J*_C-P_ = 2.7 Hz), 126.1 (d, *J*_C-P_ = 3.4 Hz), 60.5 (d, *J*_C-P_ = 6.0 Hz), 33.6 (d, *J*_C-P_ = 133.6 Hz), 16.3 (d, *J*_C-P_ = 6.0 Hz); ^31^P NMR (162 MHz, DMSO-d_6_) δ 23.3.

#### 3.6.3. Ethyl Hydrogen Ethylphosphonate (**3c**)

The synthesis of **3c** was conducted according to Section 3.5.

Yield 89%; NMR data consistent with the previous report [44]; colorless oil; ^1^H NMR (400 MHz, DMSO-d_6_) δ 3.95–3.86 (m, 2H), 1.57 (dq, *J*_H-P_ = 17.8, *J*_H-H_ = 7.7 Hz, 2H), 1.20 (t, *J* = 7.1 Hz, 3H), 1.00 (dt, *J*_H-P_ = 19.2, *J*_H-H_ = 7.7 Hz, 3H); ^13^C NMR (101 MHz, DMSO-d_6_) δ 59.9 (d, *J*_C-P_ = 6.0 Hz), 18.9 (d, *J*_C-P_ = 139.3 Hz), 16.4 (d, *J*_C-P_ = 6.1 Hz), 6.8 (d, *J*_C-P_ = 6.4 Hz); ^31^P NMR (162 MHz, DMSO-d_6_) δ 30.2.

#### 3.6.4. Ethyl Hydrogen Vinylphosphonate (**3d**)

The synthesis of **3d** was conducted according to Section 3.5.

Yield 83%; NMR data in accordance with the previous report [45]; colorless oil. ^1^H NMR (400 MHz, DMSO-d_6_) δ 9.05 (broad s, 1H), 6.21–5.91 (m, 3H), 3.88 (dq, J_H-P_ = 8.2, *J*_H-H_ = 7.1 Hz, 2H), 1.20 (t, *J*_H-H_ = 7.1 Hz, 4H); ^13^C NMR (101 MHz, DMSO-d_6_) δ 132.9, 129.8, 128.1, 60.5 (d, *J*_C-P_ = 5.2 Hz), 16.32 (d, *J*_C-P_ = 6.2 Hz); ^31^P NMR (162 MHz, DMSO-d_6_) δ 14.0; HRMS (ESI−, *m*/*z*) calcd for C_4_H_9_O_3_P; [M − H]^−^: 135.0211; found: 135.0206.

#### 3.6.5. Ethyl Hydrogen Hexylphosphonate (**3e**)

The synthesis of **3e** was conducted according to Section 3.5, with the only difference being that the reaction time was extended to 48 h.

Yield 93%; colorless oil. ^1^H NMR (400 MHz, DMSO-d_6_) δ 7.08 (broad s, 1H), 3.94–3.85 (m, 2H), 1.62–1.51 (m, 2H), 1.51–1.38 (m, 2H), 1.38–1.22 (m, 6H), 1.20 (t, *J* = 7.1 Hz, 3H), 0.86 (t, *J* = 6.9 Hz, 3H); ^13^C NMR (101 MHz, DMSO-d_6_) δ 59.8 (d, *J*_C-P_ = 5.9 Hz), 30.8, 29.6 (d, *J*_C-P_ = 15.7 Hz), 25.8 (d, *J*_C-P_ = 137.4 Hz), 22.4 (d, *J*_C-P_ = 4.9 Hz), 21.9, 16.4 (d, *J*_C-P_ = 6.1 Hz), 13.9; ^31^P NMR (162 MHz, DMSO-d_6_) δ 28.9; HRMS (ESI+, *m*/*z*) calcd for C_8_H_19_O_3_P; [M + Na]^+^: 217.0970; found: 217.0966.

#### 3.6.6. Ethyl Hydrogen Dodecylphosphonate (**3f**)

The synthesis of **3f** was conducted according to Section 3.5, with the only difference being that the reaction time was extended to 72 h.

Yield 89%; NMR data in accordance with the previous report [46]; colorless thick oil; ^1^H NMR (400 MHz, DMSO-d_6_) δ 3.94–3.84 (m, 2H), 1.62–1.50 (m, 2H), 1.50–1.38 (m, 2H), 1.38–1.22 (m, 18H), 1.20 (t, *J* = 7.1 Hz, 3H), 0.92–0.78 (t, *J* = 7.0 Hz, 3H); ^13^C NMR (101 MHz, DMSO-d_6_) δ 59.7 (d, *J*_C-P_ = 6.0 Hz), 31.3, 29.9 (d, *J*_C-P_ = 15.8 Hz), 28.99, 28.96, 28.94, 28.8, 28.7, 28.6, 25.8 (d, *J*_C-P_ = 137.2 Hz), 22.6 (d, *J*_C-P_ = 4.9 Hz), 22.0, 16.4 (d, *J*_C-P_ = 5.9 Hz), 13.9; ^31^P NMR (162 MHz, DMSO-d6) δ 28.9.

#### 3.6.7. Ethyl Hydrogen Phenylphosphonate (**3g**)

The synthesis of **3g** was conducted according to Section 3.5.

Yield 56%; NMR data consistent with reported spectra [47]; yellowish oil. ^1^H NMR (400 MHz, DMSO-d6) δ 7.79–7.62 (m, 2H), 7.63–7.53 (m, 1H), 7.52–7.39 (m, 2H), 7.02 (broad s, 1H), 3.88 (dq, *J*_H-P_ = 8.0 Hz, *J*_H-H_ = 7.0 Hz, 2H), 1.17 (t, *J*_H-H_ = 7.1 Hz, 3H); ^13^C NMR (101 MHz, DMSO-d_6_) δ 131.6 (d, *J*_C-P_ = 3.0 Hz), 131.3 (d, *J*_C-P_ = 183.0 Hz), 130.9 (d, *J*_C-P_ = 9.6 Hz), 128.4 (d, *J*_C-P_ = 14.3 Hz), 60.6 (d, *J*_C-P_ = 5.2 Hz), 16.2 (d, *J*_C-P_ = 6.4 Hz); ^31^P NMR (162 MHz, DMSO-d_6_) δ 15.0.

#### 3.6.8. Ethyl Hydrogen (4-Methoxyphenyl)phosphonate (**3h**)

The synthesis of **3h** was conducted according to Section 3.5, with the only difference being that the reaction time was extended to 96 h.

Yield 65%; NMR data consistent with reported spectra [48]; pale yellow oil. ^1^H NMR (400 MHz, DMSO-d6) δ 7.88–7.40 (m, 2H), 7.26–6.59 (m, 2H), 6.15 (broad s, -OH and H_2_O), 3.97–3.81 (m, 2H), 3.80 (s, 3H), 1.15 (t, *J* = 7.0 Hz, 1H); ^13^C NMR (101 MHz, DMSO-d_6_) δ 161.7 (d, *J*_C-P_ = 2.8 Hz), 132.9 (d, *J*_C-P_ = 11.0 Hz), 122.7 (d, *J*_C-P_ = 190.1 Hz), 113.8 (d, *J*_C-P_ = 15.3 Hz), 60.4 (d, *J*_C-P_ = 4.8 Hz), 55.3, 16.2 (d, *J*_C-P_ = 6.2 Hz); ^31^P NMR (162 MHz, DMSO-d_6_) δ 15.5.

#### 3.6.9. Ethyl Hydrogen [(4-Nitrophenyl)methyl]phosphonate (**3j**)

Due to the poor solubility of the substrate **1j** in triethyl orthoacetate and its low conversion, the reaction was carried out according to the modified Section 3.5. The reaction was conducted at 40 °C for 24 h. The excess of orthoester was then evaporated off, and 83% conversion was ascertained by ^31^P NMR analysis, and the product was isolated via extraction. 

Yield: 29%; ^1^H NMR is in accordance with foregoing literature data [49]; milky white solid; m.p. 144.1–144.7 °C; ^1^H NMR (400 MHz, DMSO-d_6_) δ 8.22–7.88 (m, 2H), 7.73–7.20 (m, 2H), 4.02 (broad s, -OH and H_2_O), 3.91 (dq, *J*_H-P_ ≈ *J*_H-H_ = 7.1 Hz, 2H), 3.27 (d, *J*_H-P_ = 22.0 Hz, 2H), 1.16 (t, *J*_H-H_ = 7.1 Hz, 3H); ^13^C NMR (101 MHz, DMSO-d_6_) δ 146.1 (d, *J*_C-P_ = 3.4 Hz), 142.4, 130.9 (d, *J*_C-P_ = 6.2 Hz), 123.1 (d, *J*_C-P_ = 2.8 Hz), 60.7 (d, *J*_C-P_ = 6.0 Hz), 33.8 (d, *J*_C-P_ = 131.2 Hz), 16.3 (d, *J*_C-P_ = 6.0 Hz).^31^P NMR (162 MHz, DMSO-d_6_) δ 21.4.

#### 3.6.10. Ethyl Hydrogen [(4-Bromophenyl)methyl]phosphonate (**3k**)

The synthesis of **3k** was conducted according to Section 3.5.

Yield 78%; NMR spectra are in accordance with literature data [50]; white solid; m.p. 84.0–85.0 °C; ^1^H NMR (400 MHz, DMSO-d_6_) δ 7.62–7.33 (m, 2H), 7.33–7.05 (m, 2H), 4.46 (broad s, -OH and H_2_O), 3.88 (dq, *J*_H-H_ = 7.0 Hz, *J*_H-P_ = 6.6 Hz, 2H), 3.05 (d, *J*_H-P_ = 21.3 Hz, 2H), 1.15 (t, *J*_H-H_ = 7.0 Hz, 3H); ^13^C NMR (101 MHz, DMSO-d_6_) δ 133.0 (d, *J*_C-P_ = 8.9 Hz), 131.9 (d, *J*_C-P_ = 6.5 Hz), 130.9 (d, *J*_C-P_ = 2.8 Hz), 119.3 (d, *J*_C-P_ = 4.3 Hz), 60.5 (d, *J*_C-P_ = 5.9 Hz), 33.0 (d, *J*_C-P_ = 133.1 Hz), 16.3 (d, *J*_C-P_ = 6.1 Hz); ^31^P NMR (162 MHz, DMSO-d_6_) δ 22.4.

#### 3.6.11. Ethyl Hydrogen [(3-Bromophenyl)methyl]phosphonate (**3l**)

The synthesis of **3l** was conducted according to Section 3.5, with the only difference being that the reaction time was extended to 48 h.

Yield 49%; white solid; m.p. 85.1–85.6 °C; ^1^H NMR (400 MHz, DMSO-d_6_) δ 7.57–7.45 (m, 1H), 7.45–7.31 (m, 1H), 7.31–7.14 (m, 2H), 6.96 (broad s, OH), 3.90 (dq, *J*_H-P_ ≈ *J*_H-H_ = 7.1 Hz, 2H), 3.10 (d, *J*_H-P_ = 21.4 Hz, 1H), 1.16 (t, *J*_H-H_ = 7.1 Hz, 1H); ^13^C NMR (101 MHz, DMSO-d_6_) δ 136.4 (d, *J*_C-P_ = 8.7 Hz), 132.3 (d, *J*_C-P_ = 6.4 Hz), 130.2 (d, *J*_C-P_ = 2.9 Hz), 129.0 (d, *J*_C-P_ = 3.3 Hz), 128.8 (d, *J*_C-P_ = 6.4 Hz), 121.3 (d, *J*_C-P_ = 3.4 Hz), 60.6 (d, *J*_C-P_ = 6.1 Hz), 33.0 (d, *J*_C-P_ = 133.3 Hz), 16.3 (d, *J*_C-P_ = 5.9 Hz); ^31^P NMR (162 MHz, DMSO-d_6_) δ 22.7; HRMS (ESI−, *m*/*z*) calcd for C_9_H_11_O_3_PBr; [M-H]^−^: 276.9637; found: 276.9629.

#### 3.6.12. Diethyl Butylphosphonate (**4a**)

The synthesis of **4a** was conducted according to Section 3.6.

Yield: 98%; ^1^H NMR data consistent with the previous report [42]; colorless oil; ^1^H NMR (400 MHz, DMSO-d_6_) δ 3.97 (m, 4H), 1.74–1.62 (m, 2H), 1.54–1.31 (m, 4H), 1.22 (t, *J*_H-H_ = 7.1 Hz, 6H), 0.87 (t, *J*_H-H_ = 7.2 Hz, 3H); ^13^C NMR (101 MHz, DMSO-d_6_) δ 60.7 (d, *J*_C-P_ = 6.3 Hz), 24.2 (d, *J*_C-P_ = 138.7 Hz), 24.1 (d, *J*_C-P_ = 5.2 Hz), 22.9 (d, *J*_C-P_ = 16.3 Hz), 16.2 (d, *J*_C-P_ = 5.7 Hz), 13.4; ^31^P NMR (162 MHz, DMSO-d_6_) δ 32.0.

#### 3.6.13. Diethyl Benzylphosphonate (**4b**)

The synthesis of **4b** was conducted according to Section 3.6.

Yield 98%; ^1^H and ^31^P NMR spectra were in accordance with a previous report [43]; transparent oil. ^1^H NMR (400 MHz, DMSO-d6) δ 7.39–7.14 (m, 5H), 3.94 (dq, *J*_H-P_ = 7.9, *J*_H-H_ = 7.0, 4H), 3.21 (d, *J*_H-P_ = 21.6 Hz, 2H), 1.16 (t, *J*_H-H_ = 7.0 Hz, 6H); ^13^C NMR (101 MHz, DMSO-d_6_) δ 132.3 (d, *J*_C-P_ = 9.0 Hz), 129.7 (d, *J*_C-P_ = 6.6 Hz), 128.2 (d, *J*_C-P_ = 3.0 Hz), 126.4 (d, *J*_C-P_ = 3.4 Hz), 61.3 (d, *J*_C-P_ = 6.5 Hz), 32.3 (d, *J*_C-P_ = 135.1 Hz), 16.1 (d, *J*_C-P_ = 5.8 Hz); ^31^P NMR (162 MHz, DMSO-d_6_) δ 26.5.

#### 3.6.14. Diethyl Ethylphosphonate (**4c**)

The synthesis of **4c** was conducted according to Section 3.6.

Yield 97%; NMR data consistent with the previous report [44]; colorless oil. ^1^H NMR (400 MHz, DMSO-d_6_) δ 4.02–3.92 (m, 4H), 1.68 (dq, *J*_H-P_ = 17.8, *J*_H-H_ = 7.6 Hz, 2H), 1.22 (t, *J*_H-H_ = 7.0 Hz, 6H), 1.01 (dt, *J*_H-P_ = 19.7, *J*_H-H_ = 7.7 Hz, 3H); ^13^C NMR (101 MHz, DMSO-d_6_) δ 60.8 (d, *J*_C-P_ = 6.3 Hz), 17.8 (d, *J*_C-P_ = 140.5 Hz), 16.3 (d, *J*_C-P_ = 5.7 Hz), 6.4 (d, *J*_C-P_ = 6.6 Hz); ^31^P NMR (162 MHz, DMSO-d_6_) δ 33.1.

#### 3.6.15. Diethyl Hexylphosphonate (**4e**)

The synthesis of **4e** was conducted according to Section 3.6. 

Yield 91%; NMR data in accordance with the previous report [51]; colorless oil; ^1^H NMR (400 MHz, DMSO-d_6_) δ 4.02–3.91 (m, 4H), 1.76–1.57 (m, 2H), 1.52–1.39 (m, 2H), 1.39–1.29 (m, 2H), 1.29–1.18 (m, 10H), 0.86 (t, *J* = 6.8 Hz, 3H); ^13^C NMR (101 MHz, DMSO-d_6_) δ 60.7 (d, *J*_H-P_ = 6.4 Hz), 30.7, 29.4 (d, *J*_H-P_ = 15.7 Hz), 24.5 (d, *J*_H-P_ = 138.4 Hz), 22.0 (d, *J*_H-P_ = 5.0 Hz), 21.8, 16.2 (d, *J*_H-P_ = 5.5 Hz), 13.8; ^31^P NMR (162 MHz, DMSO-d_6_) δ 31.9.

#### 3.6.16. Diethyl Dodecylphosphonate (**4f**)

The synthesis of **4e** was conducted according to Section 3.6. 

Yield 89%; NMR data in accordance with the previous report [48]; transparent oil; ^1^H NMR (400 MHz, DMSO-d_6_) δ 4.02–3.90 (m, 4H), 1.75–1.61 (m, 2H), 1.51–1.38 (m, 2H), 1.37–1.18 (m, 18H and t, *J* = 7.1 Hz, 6H), 0.93–0.73 (m, 3H); ^13^C NMR (101 MHz, DMSO-d_6_) δ 60.7 (d, *J*_C-P_ = 6.3 Hz), 31.3, 29.7 (d, *J*_C-P_ = 15.7 Hz), 29.0, 28.9, 28.9, 28.8, 28.7, 28.5, 24.5 (d, *J*_C-P_ = 138.4 Hz), 22.0, 22.0 (d, *J*_C-P_ = 5.2 Hz), 16.2 (d, *J*_C-P_ = 5.6 Hz), 13.9; ^31^P NMR (162 MHz, DMSO-d_6_) δ 31.9.

#### 3.6.17. Diethyl Phenylphosphonate (**4g**)

The synthesis of **4g** was conducted according to Section 3.6. 

Yield 98%; NMR data consistent with a previous report [52]; transparent oil; ^1^H NMR (400 MHz, DMSO-d_6_) δ 7.78–7.68 (m, 2H), 7.67–7.59 (m, 1H), 7.58–7.43 (m, 2H), 4.16–3.85 (m, 4H), 1.23 (t, *J* = 7.0 Hz, 6H); ^13^C NMR (101 MHz, DMSO-d_6_) δ 132.4 (d, *J*_C-P_ = 3.1 Hz), 131.2 (d, *J*_C-P_ = 9.8 Hz), 128.7 (d, *J*_C-P_ = 14.7 Hz), 128.5 (d, *J*_C-P_ = 186.3 Hz), 61.6 (d, *J*_C-P_ = 5.6 Hz), 16.1 (d, *J*_C-P_ = 6.0 Hz); ^31^P NMR (162 MHz, DMSO-d_6_) δ 17.8.

#### 3.6.18. Diethyl (4-Methoxyphenyl)phosphonate (**4h**)

The synthesis of **4h** was conducted according to Section 3.6. 

Yield 99%; NMR data in accordance with the literature [53]; colorless oil. ^1^H NMR (400 MHz, DMSO-d_6_) δ 7.68–7.59 (m, 2H), 7.11–7.03 (m, 2H), 4.03–3.91 (m, 4H), 3.82 (s, 3H), 1.21 (t, *J* = 7.1 Hz, 6H); ^13^C NMR (101 MHz, DMSO-d_6_) δ 162.4 (d, *J*_C-P_ = 3.5 Hz), 133.3 (d, *J*_C-P_ = 11.2 Hz), 119.6 (d, *J*_C-P_ = 193.2 Hz), 114.2 (d, *J*_C-P_ = 15.7 Hz), 61.3 (d, *J*_C-P_ = 5.4 Hz), 55.3, 16.1 (d, *J*_C-P_ = 6.1 Hz); ^31^P NMR (162 MHz, DMSO-d_6_) δ 18.8.

#### 3.6.19. Diethyl [(4-Hydroxyphenyl)methyl]phosphonate (**4i**)

The synthesis of **4i** was conducted according to Section 3.6.

Yield 79%; NMR spectra were in accordance with a previous report [54]; colorless oil; ^1^H NMR (400 MHz, DMSO-d_6_) δ 9.25 (s, -OH), 7.21–6.87 (m, 2H), 6.76–6.54 (m, 2H), 3.91 (dq, *J*_H-P_ ≈ J_H-H_ ≈ 7.1 Hz, 4H), 3.06 (d, *J*_H-P_ = 20.9 Hz, 1H), 1.16 (t, *J*_H-H_ = 7.1 Hz, 1H); ^13^C NMR (101 MHz, DMSO-d_6_) δ 156.0 (d, *J*_C-P_ = 3.2 Hz), 130.6 (d, *J*_C-P_ = 6.6 Hz), 121.9 (d, *J*_C-P_ = 9.1 Hz), 115.1 (d, *J*_C-P_ = 2.8 Hz), 61.2 (d, *J*_C-P_ = 6.6 Hz), 31.3 (d, *J*_C-P_ = 136.2 Hz), 16.2 (d, *J*_C-P_ = 5.7 Hz); ^31^P NMR (162 MHz, DMSO-d_6_) δ 27.1.

#### 3.6.20. Diethyl [(4-Nitrophenyl)methyl]phosphonate (**4j**)

The synthesis of **4j** was conducted according to Section 3.6. 

Yield 99%; ^1^H and ^13^C NMR spectra were in accordance with reported data [55]; pinkish oil; ^1^H NMR (400 MHz, DMSO-d^6^) δ 8.31–8.04 (m, 2H), 7.56 (m, 2H), 3.98 (dq, *J*_H-P_ = 8.4 Hz, *J*_H-H_ = 7.0 Hz, 4H), 3.45 (d, *J*_H-P_ = 22.4 Hz, 2H), 1.18 (t, *J*_H-H_ = 7.1 Hz, 6H); ^13^C NMR (101 MHz, DMSO-d_6_) δ 146.3 (d, *J*_C-P_ = 4.1 Hz), 141.0 (d, *J*_C-P_ = 9.0 Hz), 131.0 (d, *J*_C-P_ = 6.4 Hz), 123.3 (d, *J*_C-P_ = 2.9 Hz), 61.6 (d, *J*_C-P_ = 6.5 Hz), 32.3 (d, *J*_C-P_ = 133.3 Hz), 16.1 (d, *J*_C-P_ = 5.6 Hz); ^31^P NMR (162 MHz, DMSO-d_6_) δ 24.9.

#### 3.6.21. Diethyl [(4-Bromophenyl)methyl]phosphonate (**4k**)

The synthesis of **4k** was conducted according to Section 3.6. 

Yield 75%; NMR data in accordance with the literature [56]; colorless oil; ^1^H NMR (400 MHz, DMSO-d_6_) δ 7.64–7.39 (m, 2H), 7.39–7.12 (m, 2H), 3.95 (dq, *J*_H-P_ = 8.3, *J*_H-H_ = 7.1 Hz, 4H), 3.22 (d, *J*_H-P_ = 21.6 Hz, 2H), 1.17 (t, *J*_H-H_ = 7.1 Hz, 6H); ^13^C NMR (101 MHz, DMSO-d_6_) δ 131.9 (d, *J*_C-P_ = 9.0 Hz), 131.9 (d, *J*_C-P_ = 6.6 Hz), 131.1 (d, *J*_C-P_ = 3.0 Hz), 119.7 (d, *J*_C-P_ = 4.5 Hz), 61.4 (d, *J*_C-P_ = 6.5 Hz), 31.6 (d, *J*_C-P_ = 134.7 Hz), 16.1 (d, *J*_C-P_ = 5.7 Hz), ^31^P NMR (162 MHz, DMSO-d_6_) δ 25.8.

#### 3.6.22. Diethyl [(3-Bromophenyl)methyl]phosphonate (**4l**)

The synthesis of **4l** was conducted according to Section 3.6. The product was purified on a silica gel column (hexanes/ethyl acetate 1:1). 

Yield 74%; NMR data in accordance with the literature [57]; colorless liquid. ^1^H NMR (400 MHz, DMSO-d_6_) δ 7.54–7.46 (m, 1H), 7.47–7.37 (m, 1H), 7.33–7.18 (m, 2H), 3.96 (dq, *J*_H-P_ = 8.3 Hz, *J*_H-H_ = 7.0 Hz, 4H), 3.30 (d, *J*_H-P_ = 10.3 Hz, 2H), 1.17 (t, *J*_H-H_ = 7.1 Hz, 6H); ^13^C NMR (101 MHz, DMSO-d_6_) δ 135.2 (d, *J*_C-P_ = 9.0 Hz), 132.4 (d, *J*_C-P_ = 6.5 Hz), 130.3 (d, *J*_C-P_ = 2.8 Hz), 129.3 (d, *J*_C-P_ = 3.4 Hz), 128.8 (d, *J*_C-P_ = 6.5 Hz), 121.3 (d, *J*_C-P_ = 3.4 Hz), 61.4 (d, *J*_C-P_ = 6.4 Hz), 31.6 (d, *J*_C-P_ = 134.8 Hz), 16.1 (d, *J*_C-P_ = 5.8 Hz); ^31^P NMR (162 MHz, DMSO-d_6_) δ 25.9.

#### 3.6.23. Tetraethyl [1,4-Phenylenebis(methylene)]bis(phosphonate) (**4m**)

The synthesis of **4m** was conducted according to Section 3.6, with the only difference being that the reaction temperature was set to 145 °C. 

Yield 99%; NMR data in accordance with the literature report [58]; colorless solid; m.p. 75–77 °C; ^1^H NMR (400 MHz, DMSO-d_6_) δ 7.21 (s, 4H), 4.66–3.63 (m, 8H), 3.18 (d, *J*_H-P_ = 20.2 Hz, 2H), 1.16 (t, *J*_H-H_ = 7.1 Hz, 12H); ^13^C{ NMR (101 MHz, DMSO-d_6_) δ 130.4, 130.4, 130.4, 129.6, 129.6, 129.6, 129.5, 129.5, 61.3, 61.3, 61.2, 31.9 (d, *J*_C-P_ = 135.8 Hz), 16.16, 16.13, 16.11; ^31^P NMR (162 MHz, DMSO-d_6_) δ 26.5.

## 4. Conclusions

Systematic studies were undertaken to design green and sustainable synthesis of monoethyl and diethyl esters of phosphonic acids. Several alkoxy group donors were utilized for direct esterification of substrates and only for triethyl or trimethyl orthoacetate, diethoxymethyl acetate and tetramethyl orthocarbonate were the expected products obtained. Triethyl orthoacetate (**2a**) was found to be the best reagent as well as a solvent for esterification reactions. In the present studies, we discovered the effect of temperature on selective esterification of phosphonic acids. For the reactions conducted, at 30 °C the dominant formation of monoethyl esters was observed, while at 90 °C diesters **4** were obtained in good to excellent yields. The generality and applicability of the new method were verified on thirteen aromatic and aliphatic phosphonic acids. For benzylphosphonic acid (**1b**), mono- and diesterification procedures were performed in 1 and 10 mmol scales without significant loss of selectivity or reaction yield.

Additional experiments were performed to give insight into the reaction mechanism. The first step in the reaction led to the formation of highly unstable intermediate **A** which readily transforms into monoester **3** at room temperature. At a higher temperature, a subsequent reaction starts giving diesters **4** via an important pyrophosphonate **6** intermediate which was characterised by ^31^P NMR spectroscopy.

It is important to note that for both reactions, no additional reagents are required and crude products obtained after reaction are sufficiently pure for most applications.

## Figures and Tables

**Table 1:** The influence of alkoxy group donors on esterification of butylphosphonic (**1a**) and benzylphosphonic (**1b**) acids; ^1^ Section 3.2.

Entry	Substrate	Donor	Conversion of 1 [%] ^2^	Product Yield [%] ^2^	
				3	4
1	**1a**	**2a**	98	84	3
2		**2b**	25	25	0
3		**2c**	0	0	0
4		**2d**	0	0	0
5		**2e**	0	0	0
6		**2f**	0	0	0
7		**2g**	39	34	0
8		**2h**	24	23	1
9		**2i**	0	0	0
10		**2j**	0	0	0
11		**2k**	0	0	0
12		**2l**	0	0	0
13		**2m**	0	0	0
14		**2n**	0	0	0
15	**1b**	**2a**	89	83	1
16		**2b**	50	49	1
17		**2c**	0	0	0
18		**2d**	0	0	0
19		**2e**	0	0	0
20		**2f**	0	0	0
21		**2g**	89	83	6
22		**2h**	52	44	8
23		**2i**	0	0	0
24		**2j**	0	0	0
25		**2k**	0	0	0
26		**2l**	0	0	0
27		**2m**	0	0	0
28		**2n**	0	0	0

^1^ Reaction conditions: phosphonic acid **1a**–**b** (1 equiv., 0.05 mmol), respective donor **2a**–**n** (3 equiv., 0.15 mmol) in 1 mL of MTBE at 30 °C for 24 h. ^2^ The substrate conversion and product yield determined by ^31^P NMR.

**Table 2:** The influence of a solvent on the substrate conversion and the selectivity of esterification reaction with triethyl orthoacetate (**2a**); ^1^ Section 3.3.

Entry	Solvent	Substrate 1a Conversion [%] ^2^	Product Yield [%] ^2^		Substrate 1b Conversion [%] ^2^	Product Yield [%] ^2^	
			3a	4a		3b	4b
1	MTBE	89	87	2	90	85	5
2	THF	66	62	1	89	80	1
3	DCM	88	78	1	98	84	3
4	EtOAc	76	75	1	68	66	2
5	MeCN	71	65	2	50	41	2
6	Me_2_CO	15	10	0	3	2	1
7	PhMe	76	74	2	89	81	3
8	Neat ^3^	95	89	5	95	88	7
9	DMSO	0	0	0	0	0	0
10	DMF	0	0	0	0	0	0

^1^ Reaction conditions: phosphonic acid **1a**–**b** (1 equiv., 0.05 mmol), triethyl orthoacetate (3 equiv., 0.15 mmol) in 1 mL of solvent at 30 °C for 24 h. ^2^ The substrate conversion and product yield determined by ^31^P NMR. ^3^ One milliliter of triethyl orthoacetate.

**Table 3:** Temperature influence on the selectivity of esterification of butylphosphonic acid (**1a**) with triethyl orthoacetate (**2a**); ^1^ Section 3.4.

Entry	Temperature [°C]	Substrate 1a Conversion [%] ^2^	Product Yield [%] ^2^	
			3a	4a
1	30	97	92	5
2	40	>99	94	6
3	50		87	13
4	60		73	27
5	70		29	66
6	80		21	79
7	90		1	96
8	100		0	95

^1^ Reaction conditions: butylphosphonic acid **1a** (1 equiv., 0.1 mmol), triethyl orthoacetate (1 mL). ^2^ The substrate conversion and product yield determined by ^31^P NMR.

**Table 4:** The yields of mono- (**3**) and diesters (**4**) obtained from respective phosphonic acids (**1**) with triethyl orthoacetate **2a** as ethoxy group donor and solvent; ^1^ Section 3.5 and Section 3.6.

Entry	R	Product	Conditions	Yield [%] ^2^	^31^P NMR [ppm]
1	*n*-Butyl	**3a**	30 °C, 24 h	98	29.1
2		**4a**	90 °C, 24 h	98	32.0
3	Benzyl	**3b**	30 °C, 48 h	76	23.3
4		**4b**	90 °C, 24 h	98	26.5
5	Ethyl	**3c**	30 °C, 96 h	89	30.2
6		**4c**	90 °C, 24 h	97	33.1
7	Vinyl	**3d**	25 °C, 24 h	83	14.0
8 ^3^		**4d**	90 °C, 24 h	<1	16.9
9	*n*-Hexyl	**3e**	30 °C, 48 h	93	28.9
10		**4e**	90 °C, 24 h	91	31.9
11	*n*-Dodecyl	**3f**	30 °C, 72 h	89	28.9
12		**4f**	90 °C, 24 h	89	31.9
13	Phenyl	**3g**	30 °C, 24 h	56	15.0
14		**4g**	90 °C, 24 h	98	17.8
15	4-methoxyphenyl	**3h**	30 °C, 96 h	65	15.5
16		**4h**	90 °C, 24 h	99	18.8
17 ^4^	4-hydroxybenzyl	**3i**	30 °C, 96 h	<1	-
18		**4i**	90 °C, 24 h	79	27.1
19	4-nitrobenzyl	**3j**	40 °C, 24 h	29	21.4
20		**4j**	90 °C, 24 h	99	24.9
21	4-bromobenzyl	**3k**	30 °C, 24 h	78	22.4
22		**4k**	90 °C, 24 h	75	25.8
23	3-bromobenzyl	**3l**	30 °C, 48 h	49	22.7
24		**4l**	90 °C, 24 h	74	25.9
25 ^4^	*p*-xylylene	**3m**	30 °C, 48 h	<1	-
26		**4m**	145 °C, 24 h	99	26.5

^1^ Monoesterification and diesterification were conducted according to Section 3.5 and Section 3.6, respectively. Reactions were monitored by ^31^P NMR and terminated after complete conversion of substrate. ^2^ The yield of isolated product. ^3^ The substrate readily polymerized at elevated temperatures. ^4^ No reaction, due to insolubility of substrate.

## Data Availability

On request to those interested.

## Supplementary Materials

The following are available online. Table S1: Summarized data from the ^31^P NMR measurements during the course of the esterification at elevated temperature, Table S2: Recorded ^31^P NMR shifts for substrate phosphonic acids, Table S3: Recorded ^31^P NMR shifts for obtained mono- and diesters, as well as ^1^H, ^13^C and ^31^P NMR spectra for all isolated products.

## Sample Availability

Samples of the compounds are available from the authors.
