# Supplementary material for: Selective Esterification of Phosphonic Acids
Source: Molecules. 2021 Sep 17;26(18):5637. doi: 10.3390/molecules26185637 (PMC8466293; doi:10.3390/molecules26185637)
Supplement: Supplementary file 1 [file molecules-26-05637-s001.zip › molecules-1342104-supplementary.pdf]

molecules

Supporting Information

Selective Esterification of Phosphonic Acids †

Damian Trzepizur, Anna Brodzka, Dominik Koszelewski and Ryszard Ostaszewski *

Institute of Organic Chemistry, Polish Academy of Science, Kasprzaka 44/52, 01-224 Warsaw, Poland; dtrzepizur@icho.edu.pl (D.T.); awoltanska@icho.edu.pl (A.B.); dkoszelewski@icho.edu.pl (D.K.)
* Correspondence: ryszard.ostaszewski@icho.edu.pl

† This work is dedicated to Professor Janusz Jurczak on the occasion of his 80th birthday.

Table of Contents

1. Results of additional experiments

1.1. *Studies on the reaction progress* 3

2. Analytical data for the studied compounds

2.1. *Structures and ^{31}P NMR chemical shifts for applied substrates* 4

2.2. *Symbols, structures, and ^{31}P NMR chemical shifts of received products* 5

2.3. *^{1}H, ^{13}C, and ^{31}P NMR spectra of the monoesters* 6

2.4. *^{1}H, ^{13}C, and ^{31}P NMR spectra of the diesters* 23

1. Results of additional experiments

1.1. *Studies on the reaction progress*

Table S1. Summarized data from the ^{31}P NMR measurements during the course of the esterification at elevated temperature. [1]

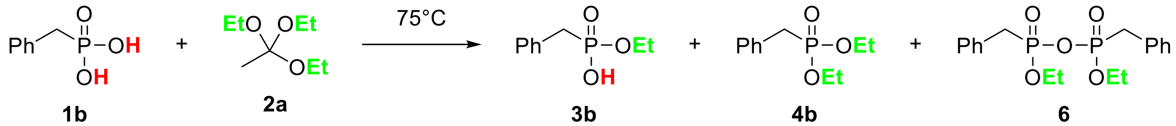

Entry	Time of reaction [h]	Substrate **1b** conversion [%]	Product yield [%] [2]		
			**3b**	**4b**	**6**
1	1	>99	21	46	34
2	2		13	53	34
3	4		8	60	32
4	6		4	64	31
5	8		0	74	26
6	24		0	99	0

[1] Reaction conditions: benzylphosphonic acid **1b** (1 equiv., 0.5 mmol), triethyl orthoacetate (30 equiv., 15 mmol). [2] The conversion of **1b** and product yields were determined on the basis of relative ^{31}P NMR integrals.

2. Analytical data for the studied compounds

2.1. *Structures and ^{31}P NMR chemical shifts for applied substrates*

Table S2. Recorded ^{31}P NMR shifts for substrate phosphonic acids.

Symbol	Chemical Name	Structure	^{31}P NMR chemical shift [ppm] [1]
**1a**	*n*-butylphosphonic acid	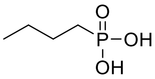	27.8
**1b**	benzylphosphonic acid	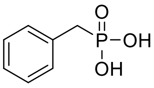	21.3
**1c**	ethylphosphonic acid	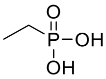	29.1
**1d**	vinylphosphonic acid	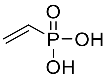	12.0
**1e**	*n*-hexylphosphonic acid	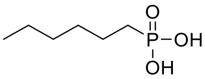	27.4
**1f**	*n*-dodecylphosphonic acid	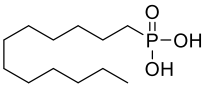	27.6
**1g**	phenylphosphonic acid	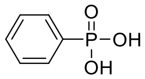	13.0
**1h**	(4-methoxyphenyl)phosphonic acid	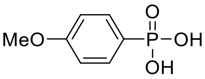	13.7
**1i**	[(4-hydroxyphenyl)methyl]-phosphonic acid	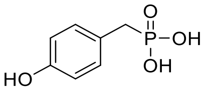	22.4
**1j**	[(4-nitrophenyl)methyl]-phosphonic acid	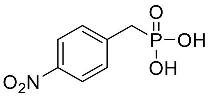	24.9
**1k**	[(4-bromophenyl)methyl]-phosphonic acid	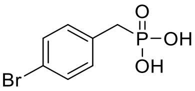	20.6
**1l**	[(3-bromophenyl)methyl]-phosphonic acid	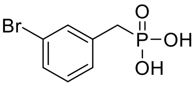	20.5
**1m**	[1,4-phenylenebis(methylene)]-bis(phosphonic acid)	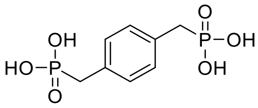	21.1

[1] Chemical shifts were measured for samples dissolved in dimethyl sulfoxide-d_6 (DMSO-d_6).

2.2. *Symbols, structures, and ^{31}P NMR chemical shifts of received products*

Table S3. Recorded ^{31}P NMR shifts for obtained mono- and diesters.

Symbol	Monoester structure	^{31}P NMR shift [ppm] [1]	Symbol	Diester structure	^{31}P NMR shift [ppm] [1]
**3a**	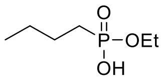	29.1	**4a**	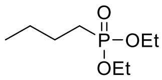	32.0
**3b**	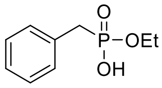	23.3	**4b**	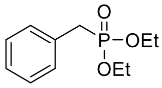	26.5
**3c**	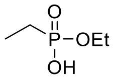	30.2	**4c**	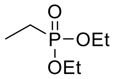	33.1
**3d**	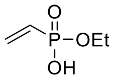	14.0	**4d**	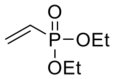	16.9
**3e**	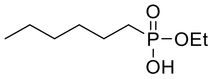	28.9	**4e**	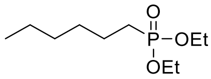	31.9
**3f**	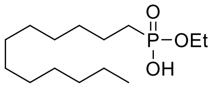	28.9	**4f**	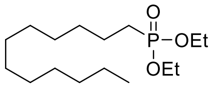	31.9
**3g**	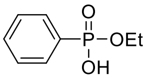	15.0	**4g**	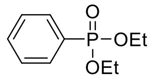	17.8
**3h**	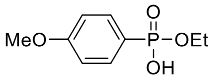	15.5	**4h**	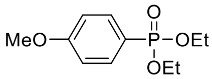	18.8
**3i**	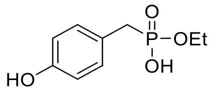	24.2	**4i**	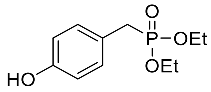	27.1
**3j**	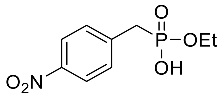	21.4	**4j**	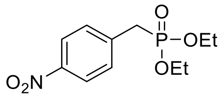	24.9
**3k**	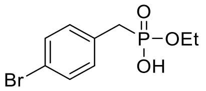	22.4	**4k**	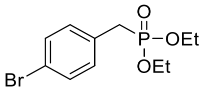	25.8
**3l**	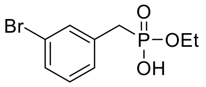	22.7	**4l**	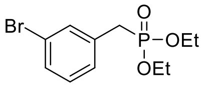	25.9
**3m**	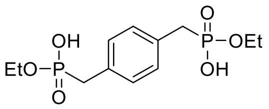	-	**4m**	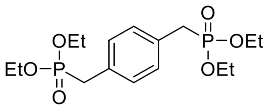	26.5

[1] Chemical shifts were measured for samples also dissolved in DMSO-d_6.

2.3. *^{1}H, ^{13}C, and ^{31}P NMR spectra of the monoesters*

Ethyl hydrogen butylphosphonate (**3a**)

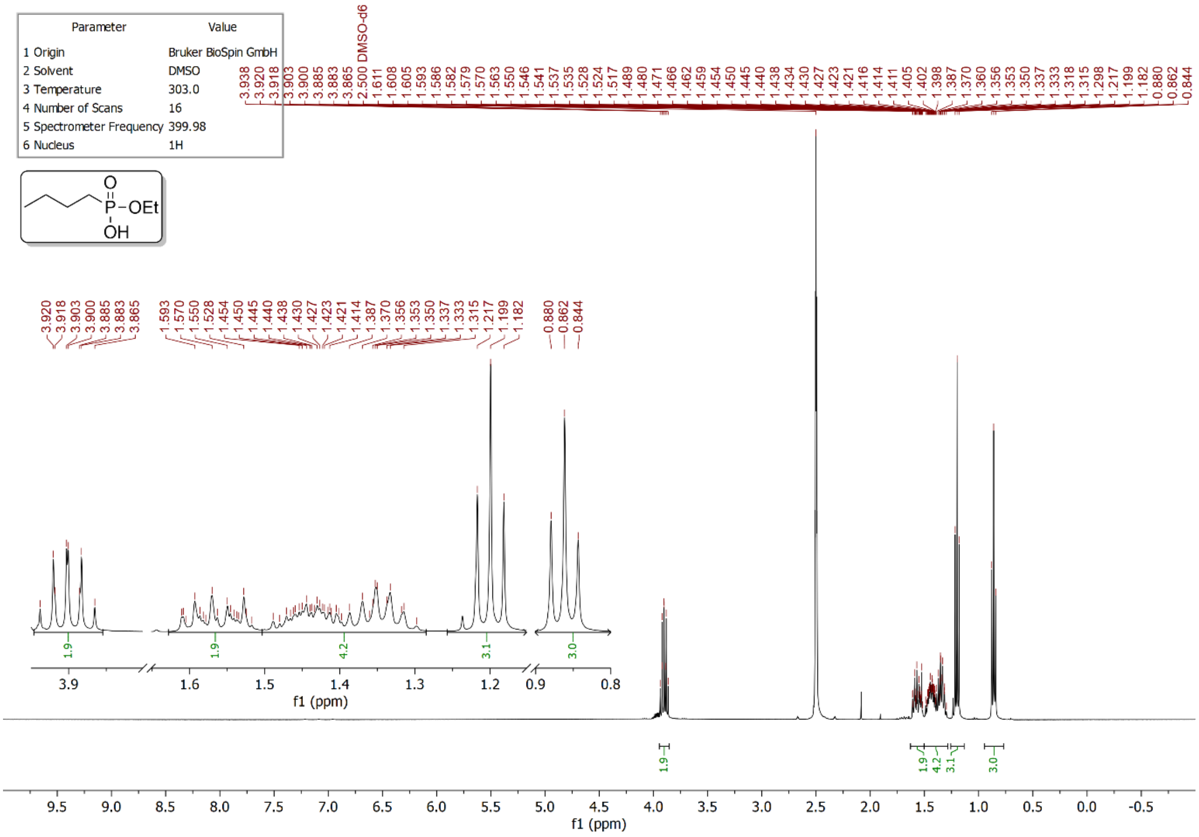

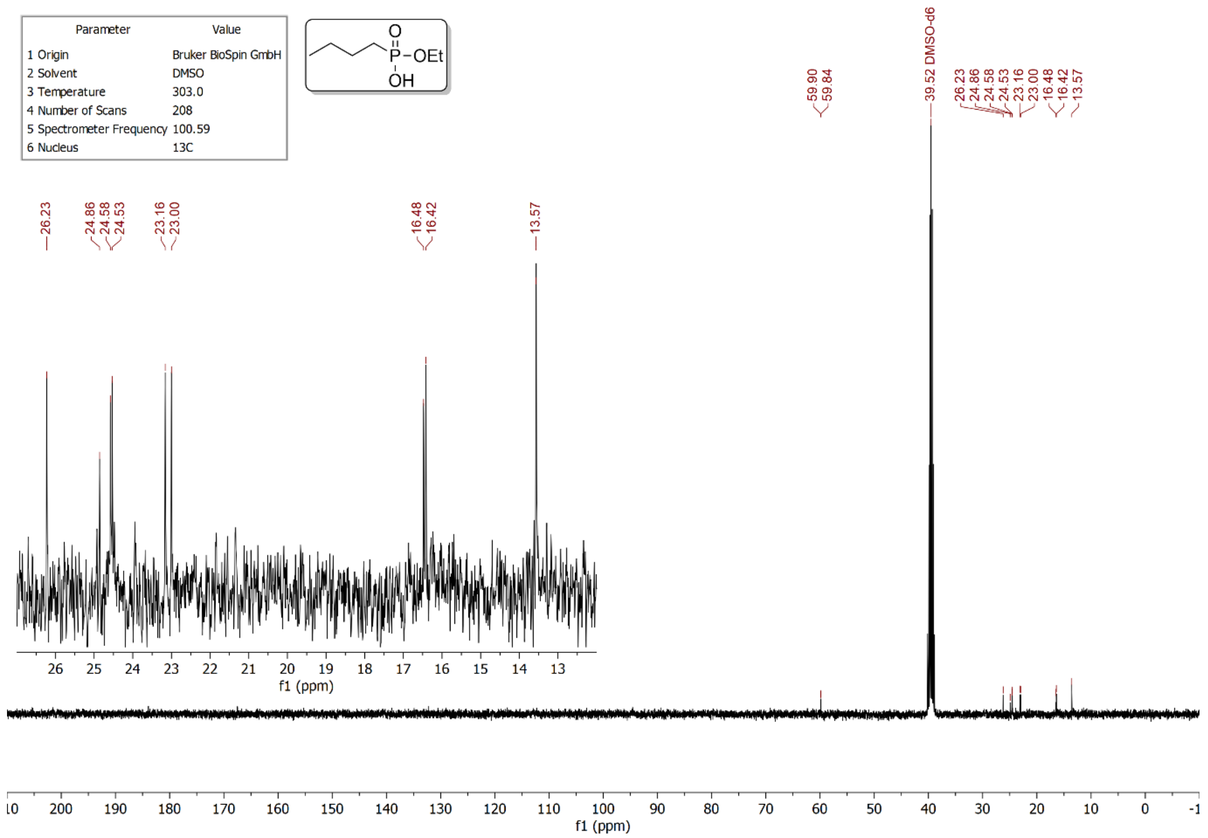

Parameter Value
1 Origin Bruker BioSpin GmbH
2 Solvent DMSO
3 Temperature 303.0
4 Number of Scans 208
5 Spectrometer Frequency 100.59
6 Nucleus 13C
P-OEt
OH
59.90
59.84
39.52 DMSO-d6
26.23
24.86
24.58
24.53
23.16
23.00
16.48
16.42
13.57
f1 (ppm)

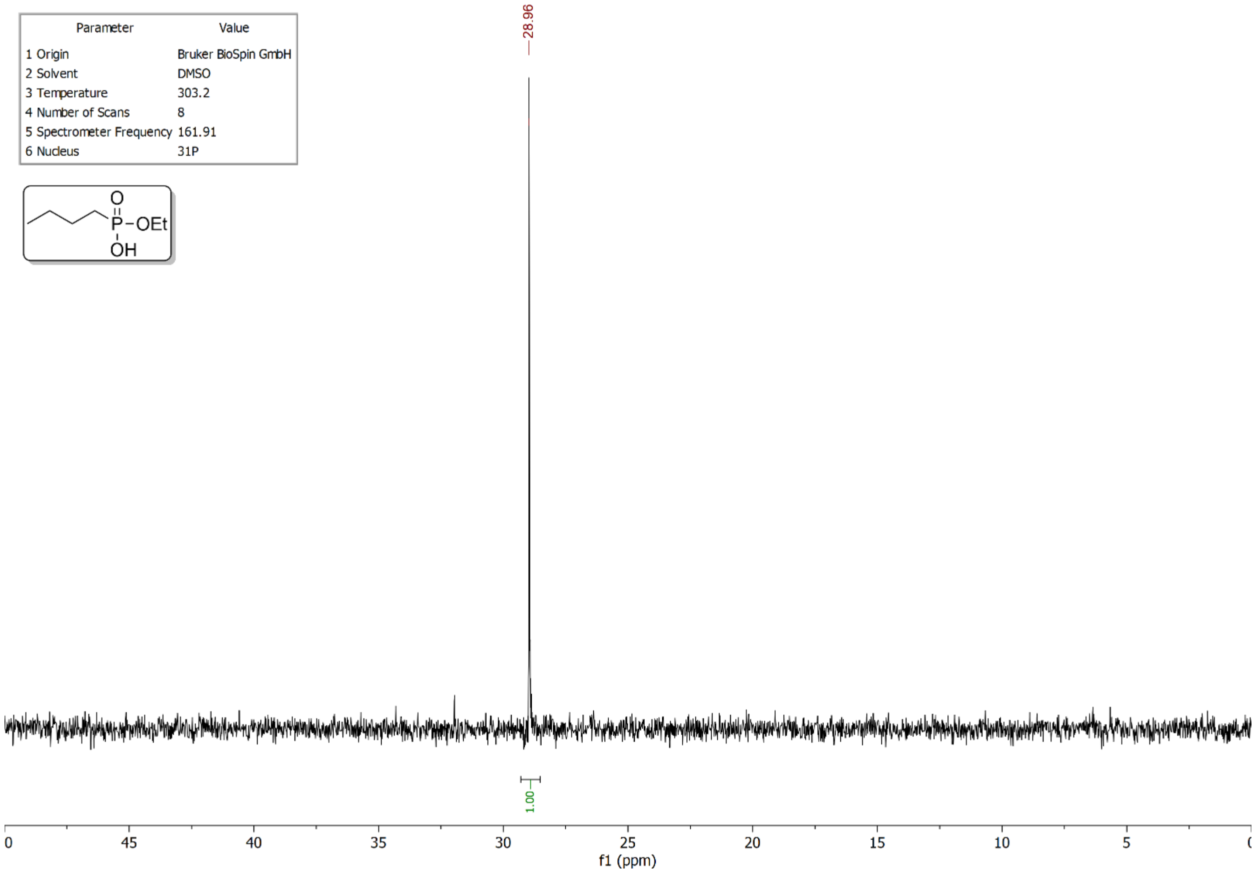

Parameter Value
1 Origin Bruker BioSpin GmbH
2 Solvent DMSO
3 Temperature 303.2
4 Number of Scans 8
5 Spectrometer Frequency 161.91
6 Nucleus 31P
P-OEt
OH
28.96
1.00
f1 (ppm)

Ethyl hydrogen benzylphosphonate (**3b**)

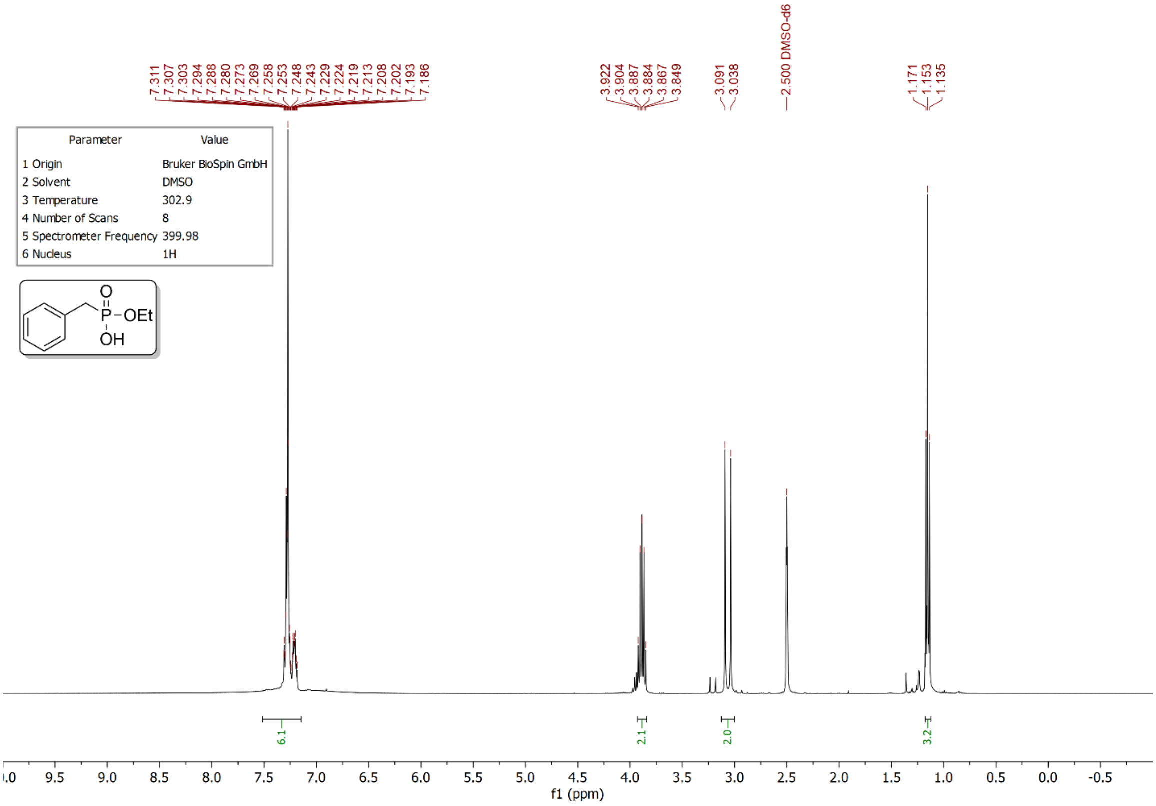

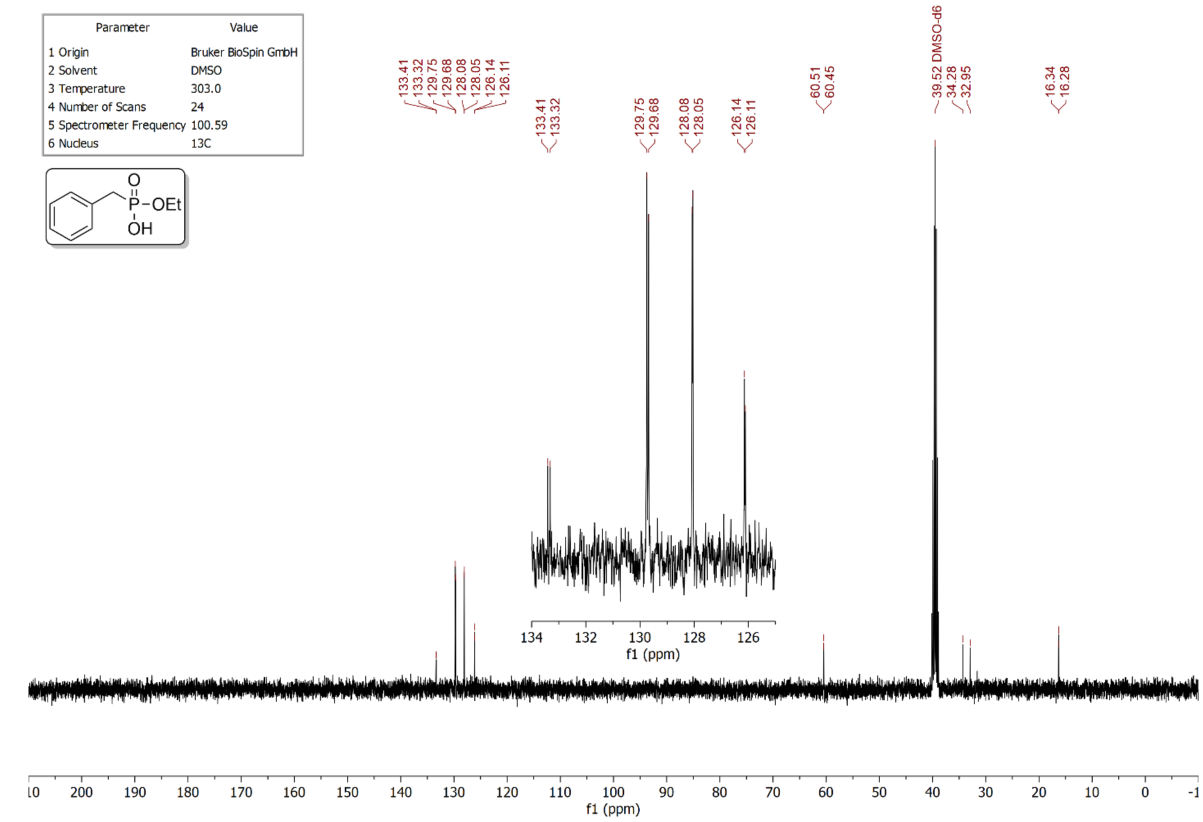
Parameter Value
1 Origin Bruker BioSpin GmbH
2 Solvent DMSO
3 Temperature 303.0
4 Number of Scans 24
5 Spectrometer Frequency 100.59
6 Nucleus 13C
P-OEt
OH
133.41
133.32
129.75
129.68
128.08
128.05
126.14
126.11
60.51
60.45
39.52 DMSO-d6
34.28
32.95
16.34
16.28
f1 (ppm)

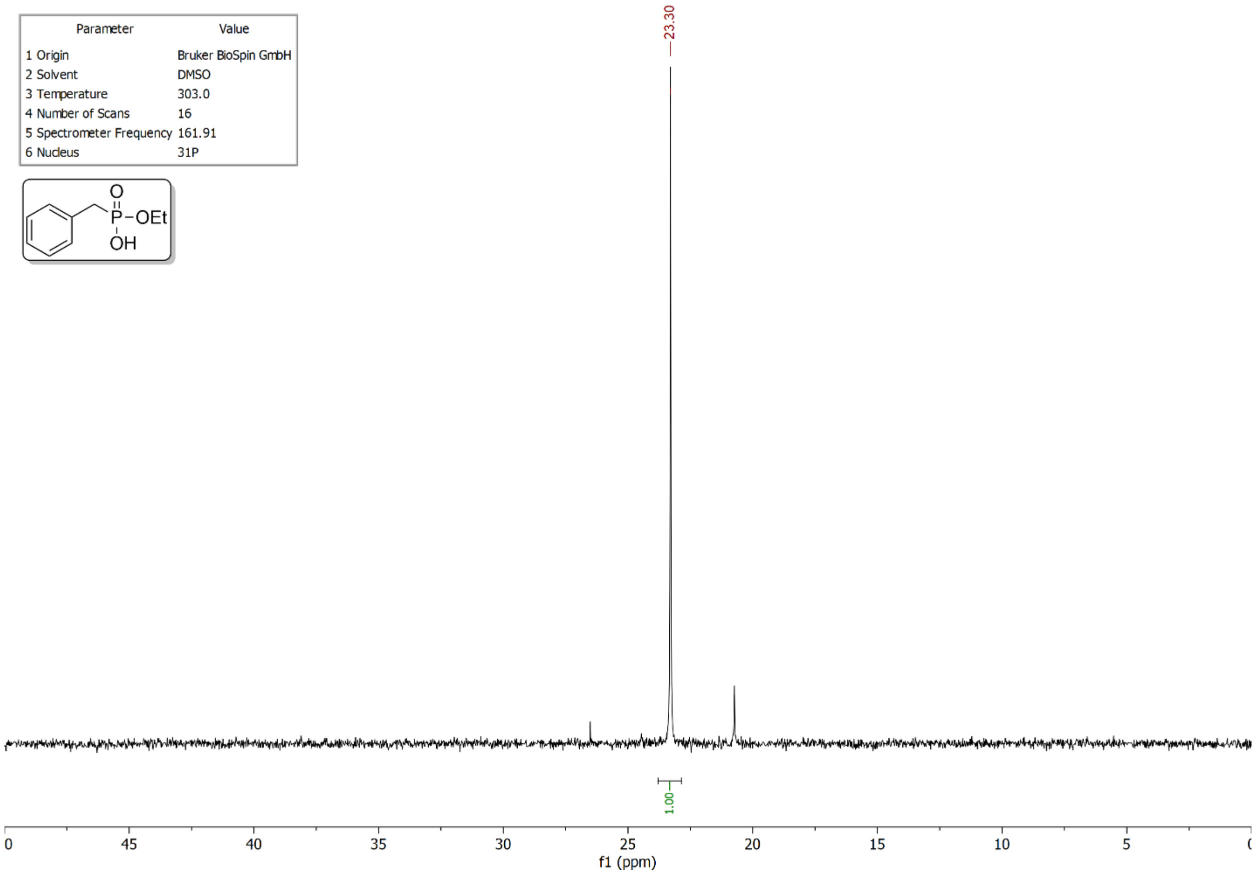
Parameter Value
1 Origin Bruker BioSpin GmbH
2 Solvent DMSO
3 Temperature 303.0
4 Number of Scans 16
5 Spectrometer Frequency 161.91
6 Nucleus 31P
P-OEt
OH
23.30
1.00
f1 (ppm)

Ethyl hydrogen ethylphosphonate (**3c**)

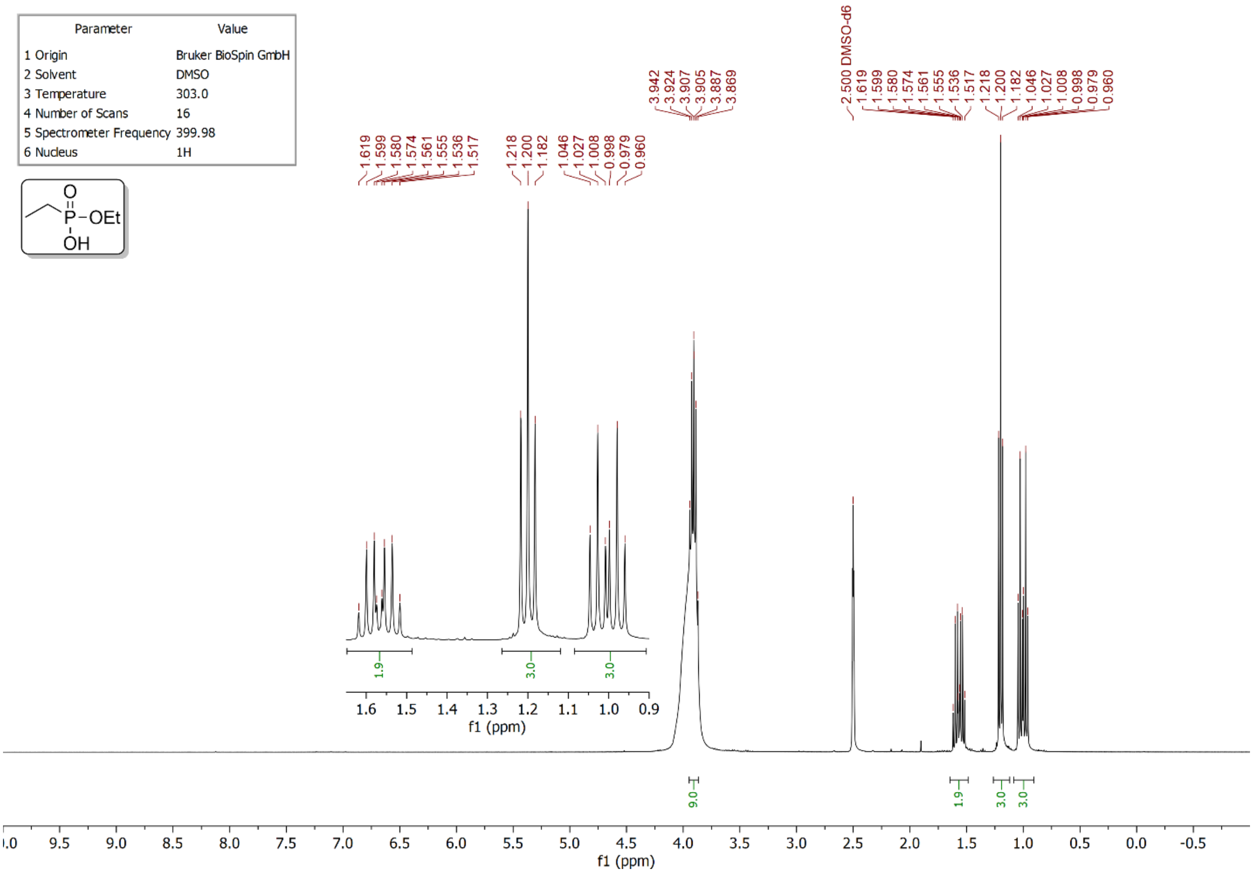

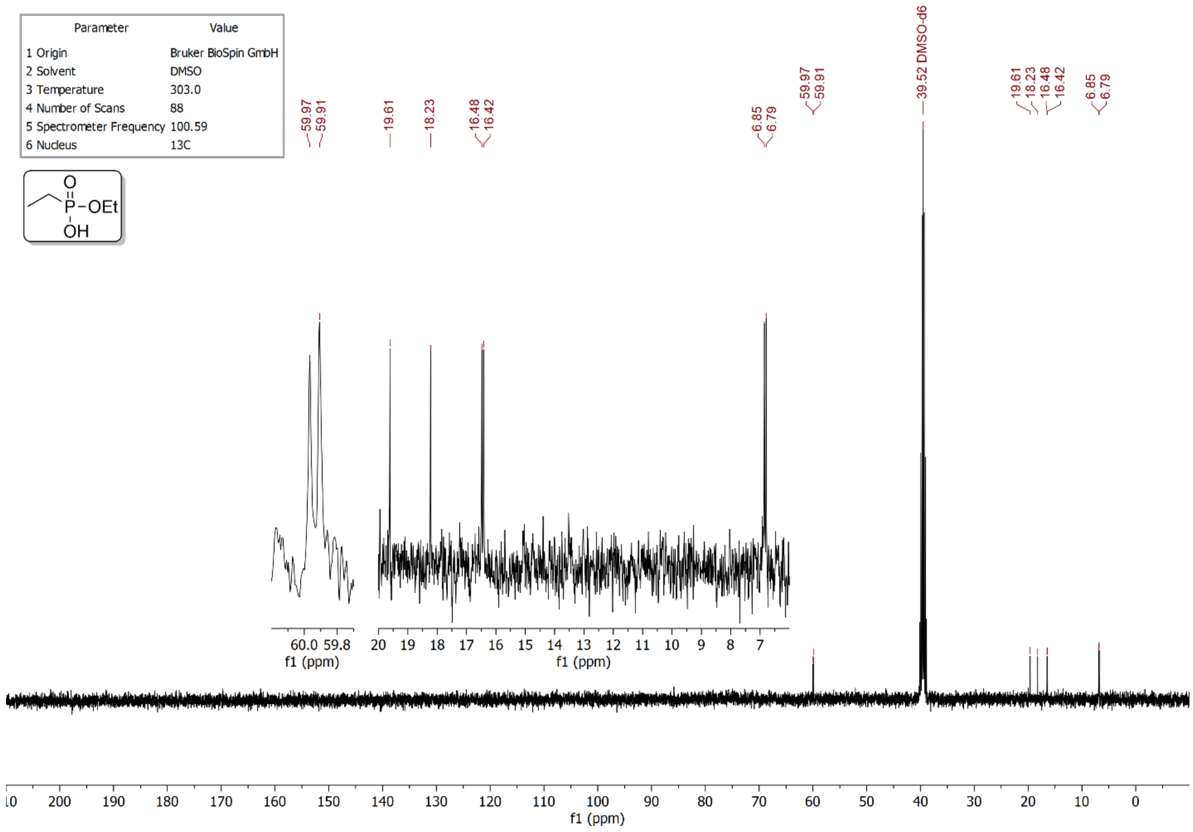
Parameter Value
1 Origin Bruker BioSpin GmbH
2 Solvent DMSO
3 Temperature 303.0
4 Number of Scans 88
5 Spectrometer Frequency 100.59
6 Nucleus 13C
O
P-OEt
OH
59.97
59.91
19.61
18.23
16.48
16.42
6.85
6.79
39.52 DMSO-d6
60.0 59.8
f1 (ppm)
f1 (ppm)

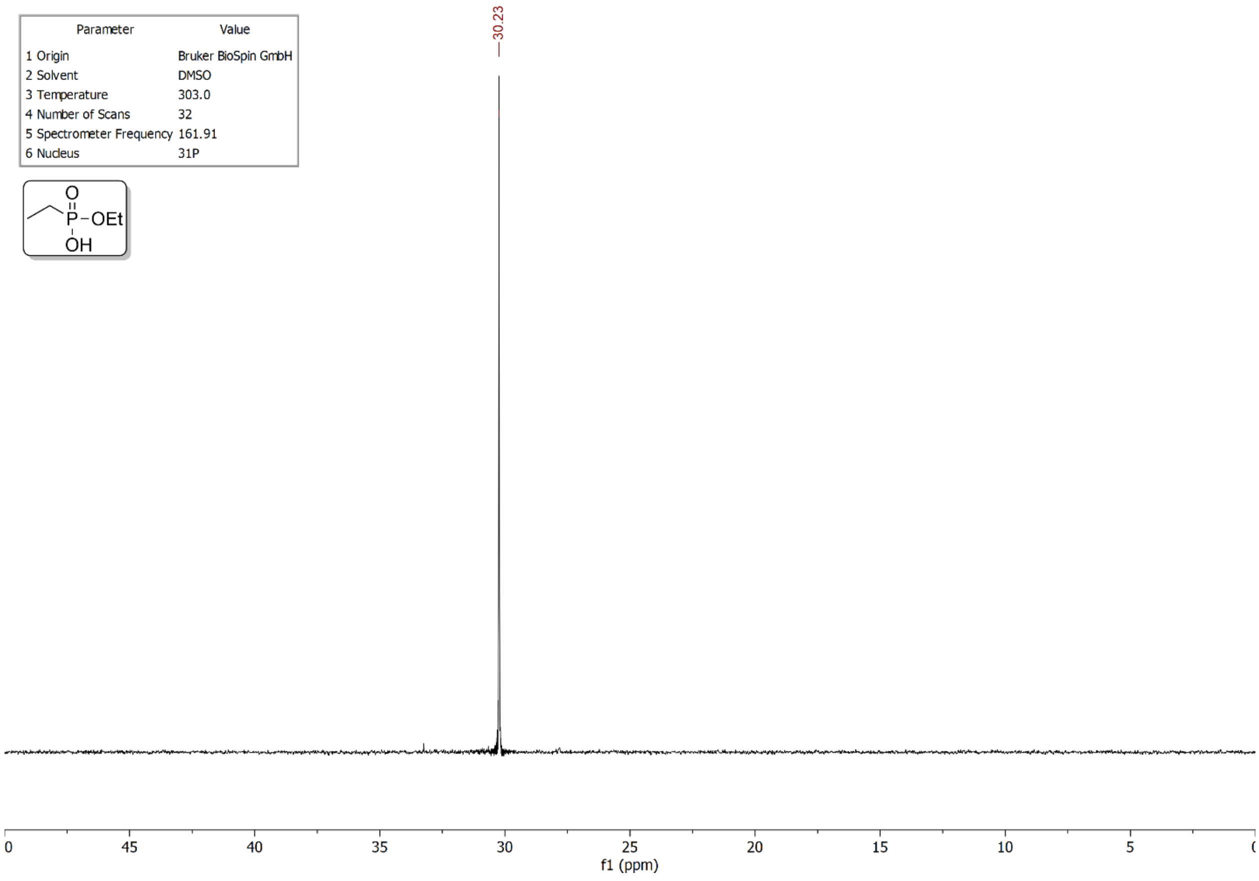
Parameter Value
1 Origin Bruker BioSpin GmbH
2 Solvent DMSO
3 Temperature 303.0
4 Number of Scans 32
5 Spectrometer Frequency 161.91
6 Nucleus 31P
O
P-OEt
OH
30.23
f1 (ppm)

Ethyl hydrogen vinylphosphonate (**3d**)

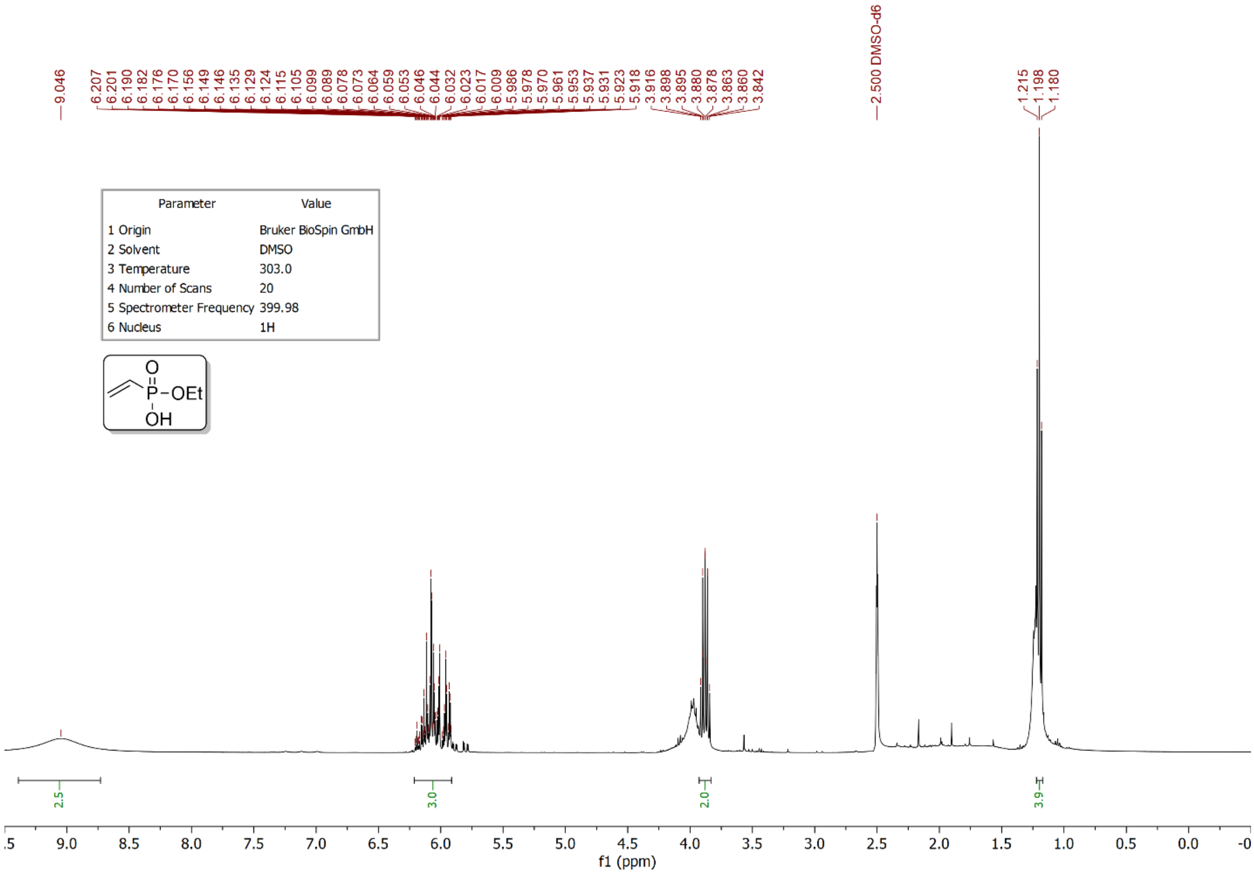

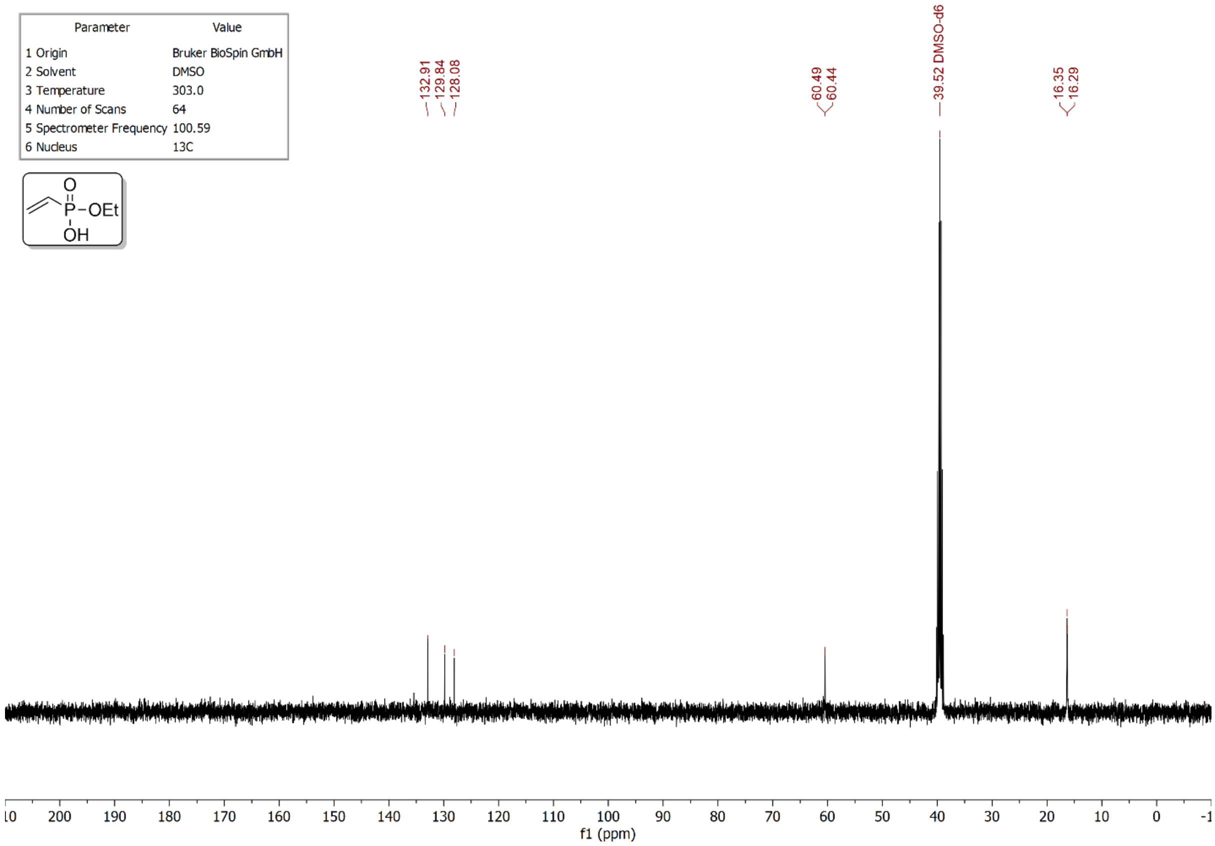
Parameter Value
1 Origin Bruker BioSpin GmbH
2 Solvent DMSO
3 Temperature 303.0
4 Number of Scans 64
5 Spectrometer Frequency 100.59
6 Nucleus 13C
O
P–OEt
OH
132.91
129.84
128.08
60.49
60.44
39.52 DMSO-d6
16.35
16.29
f1 (ppm)

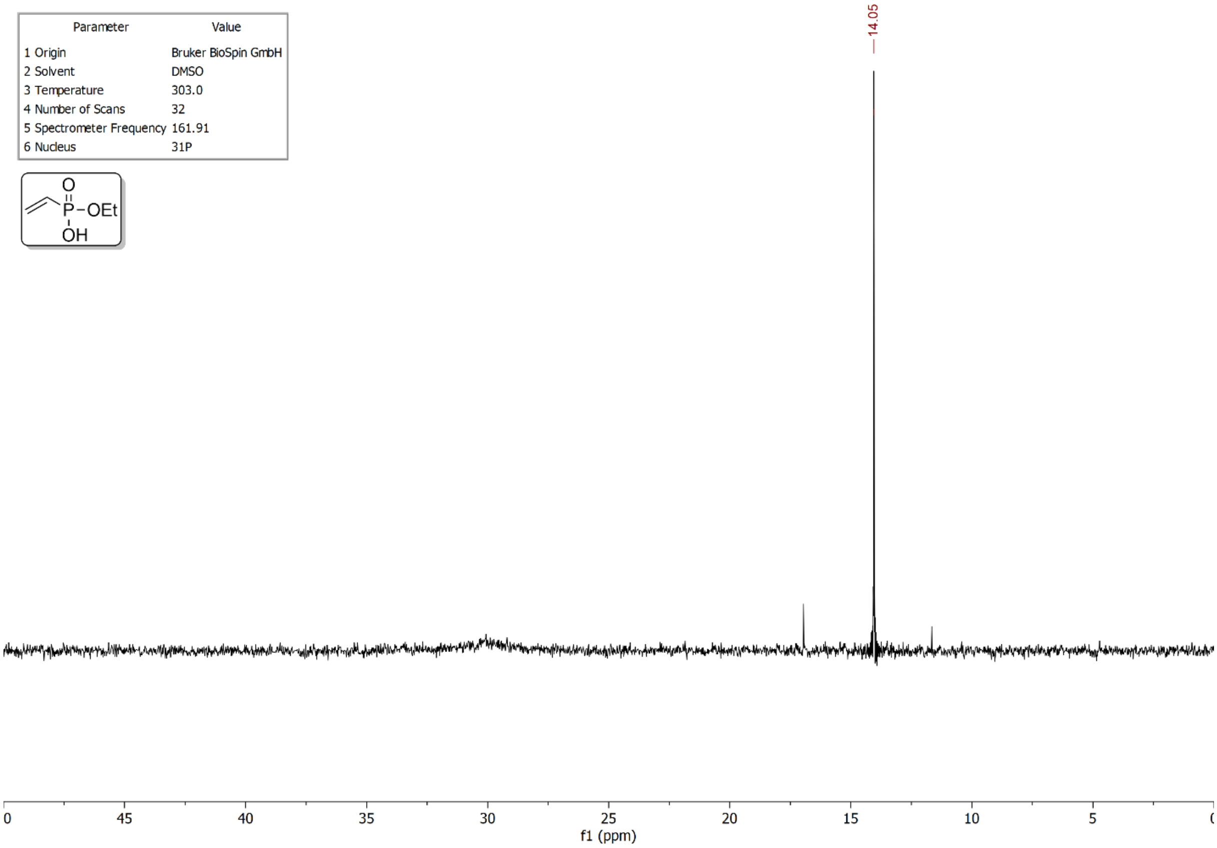
Parameter Value
1 Origin Bruker BioSpin GmbH
2 Solvent DMSO
3 Temperature 303.0
4 Number of Scans 32
5 Spectrometer Frequency 161.91
6 Nucleus 31P
O
P–OEt
OH
14.05
f1 (ppm)

Ethyl hydrogen hexylphosphonate (**3e**)

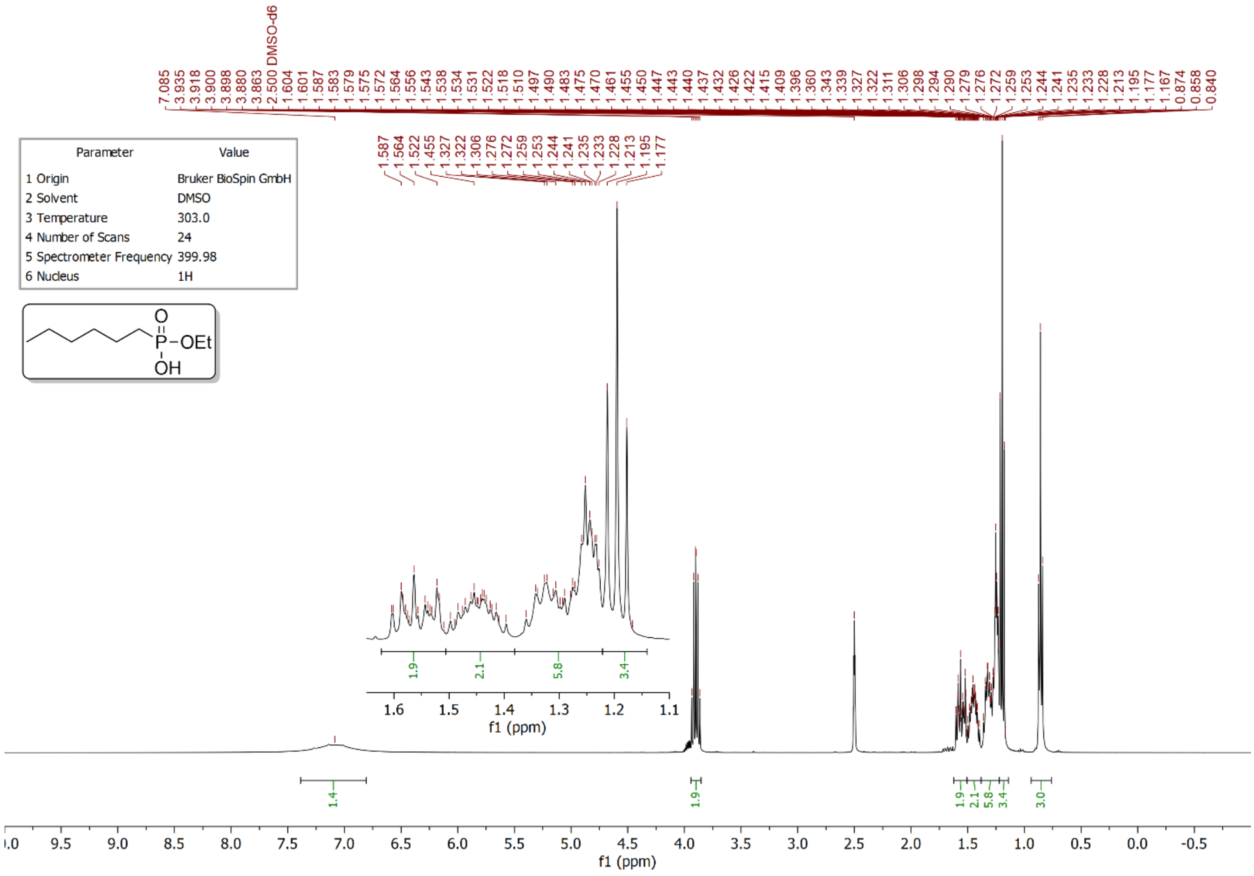

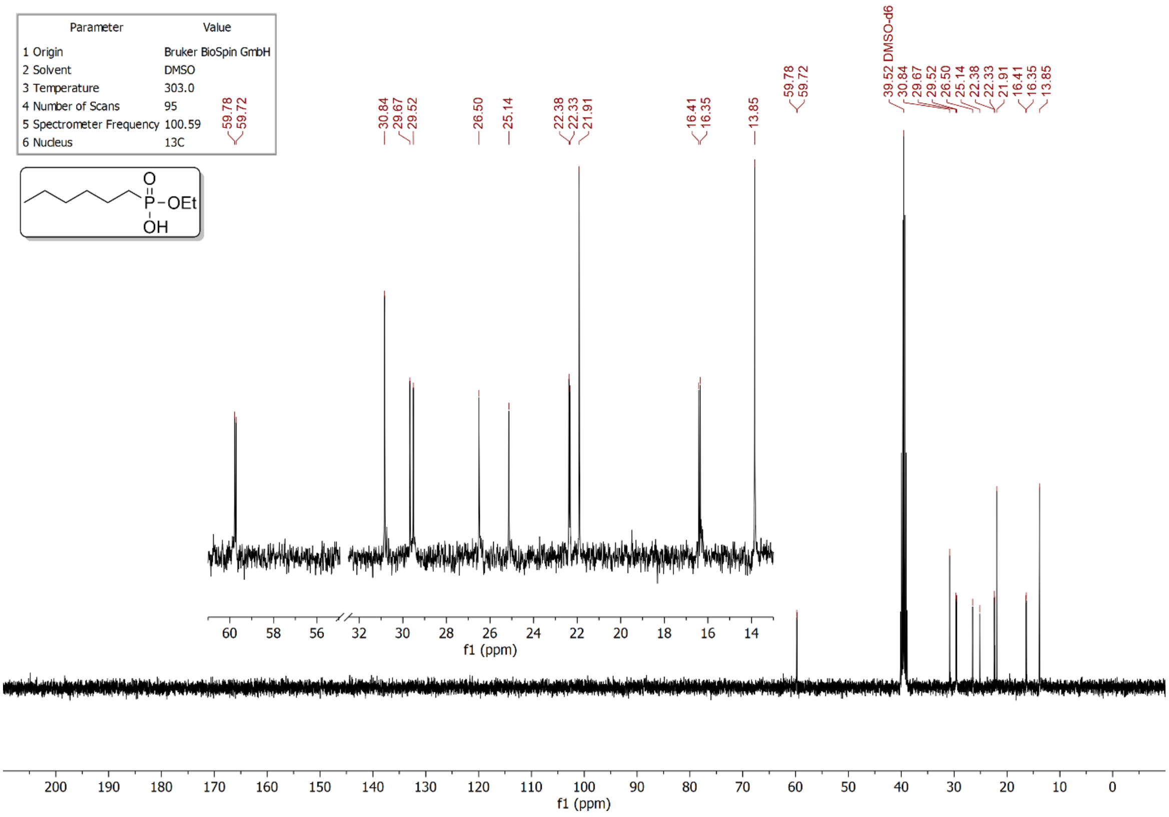

Parameter Value
1 Origin Bruker BioSpin GmbH
2 Solvent DMSO
3 Temperature 303.0
4 Number of Scans 95
5 Spectrometer Frequency 100.59
6 Nucleus 13C
P-OEt
OH
59.78
59.72
30.84
29.67
29.52
26.50
25.14
22.38
22.33
21.91
16.41
16.35
13.85
39.52 DMSO-d6
f1 (ppm)

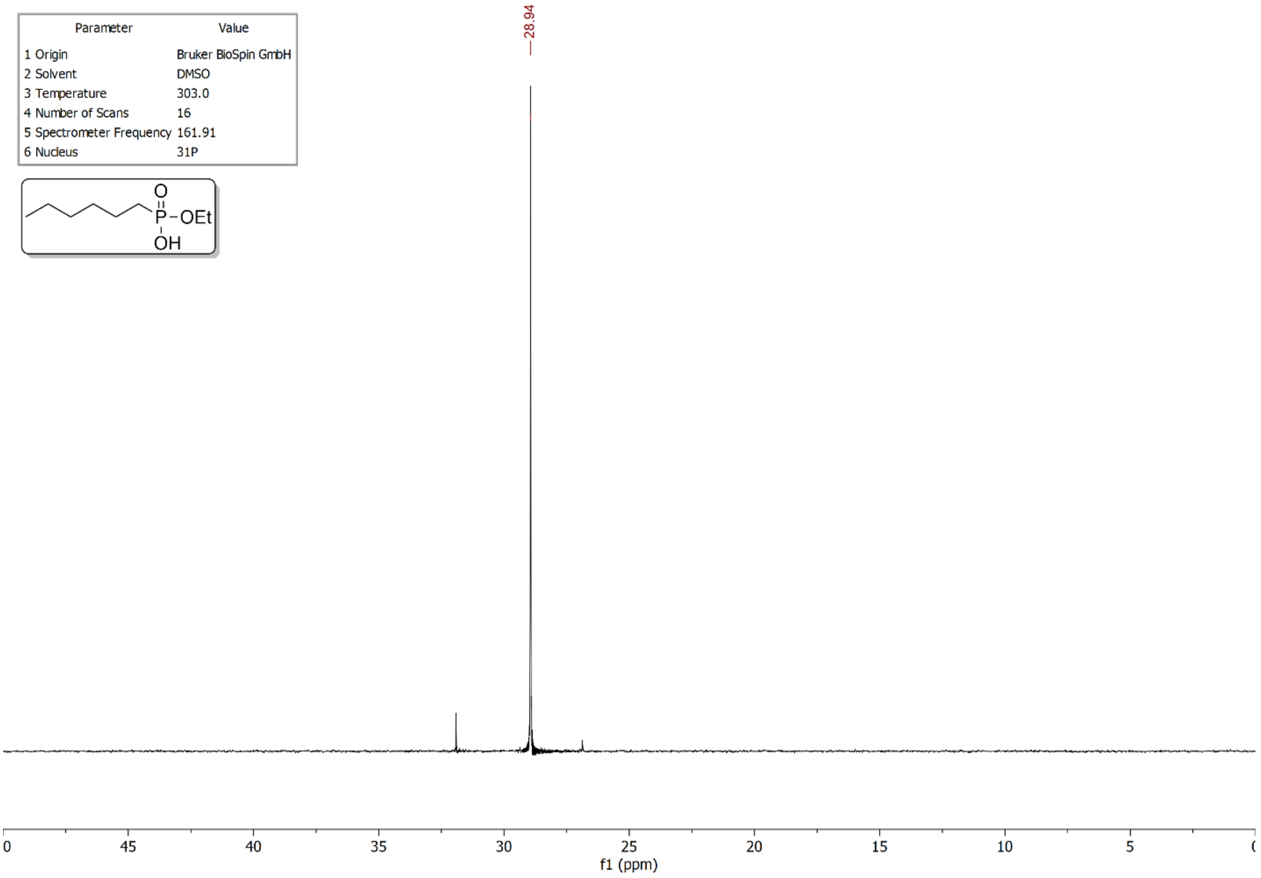

Parameter Value
1 Origin Bruker BioSpin GmbH
2 Solvent DMSO
3 Temperature 303.0
4 Number of Scans 16
5 Spectrometer Frequency 161.91
6 Nucleus 31P
P-OEt
OH
28.94
f1 (ppm)

Ethyl hydrogen dodecylphosphonate (**3f**)

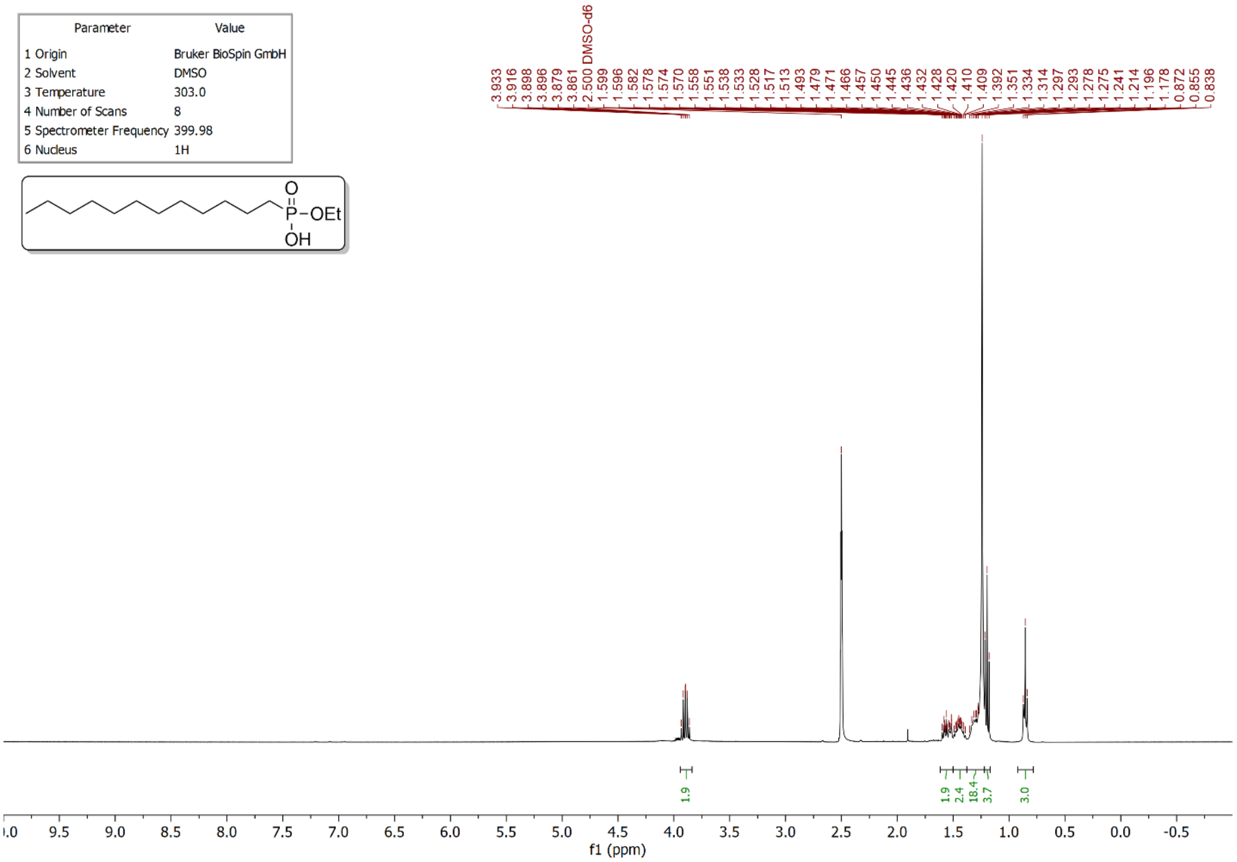

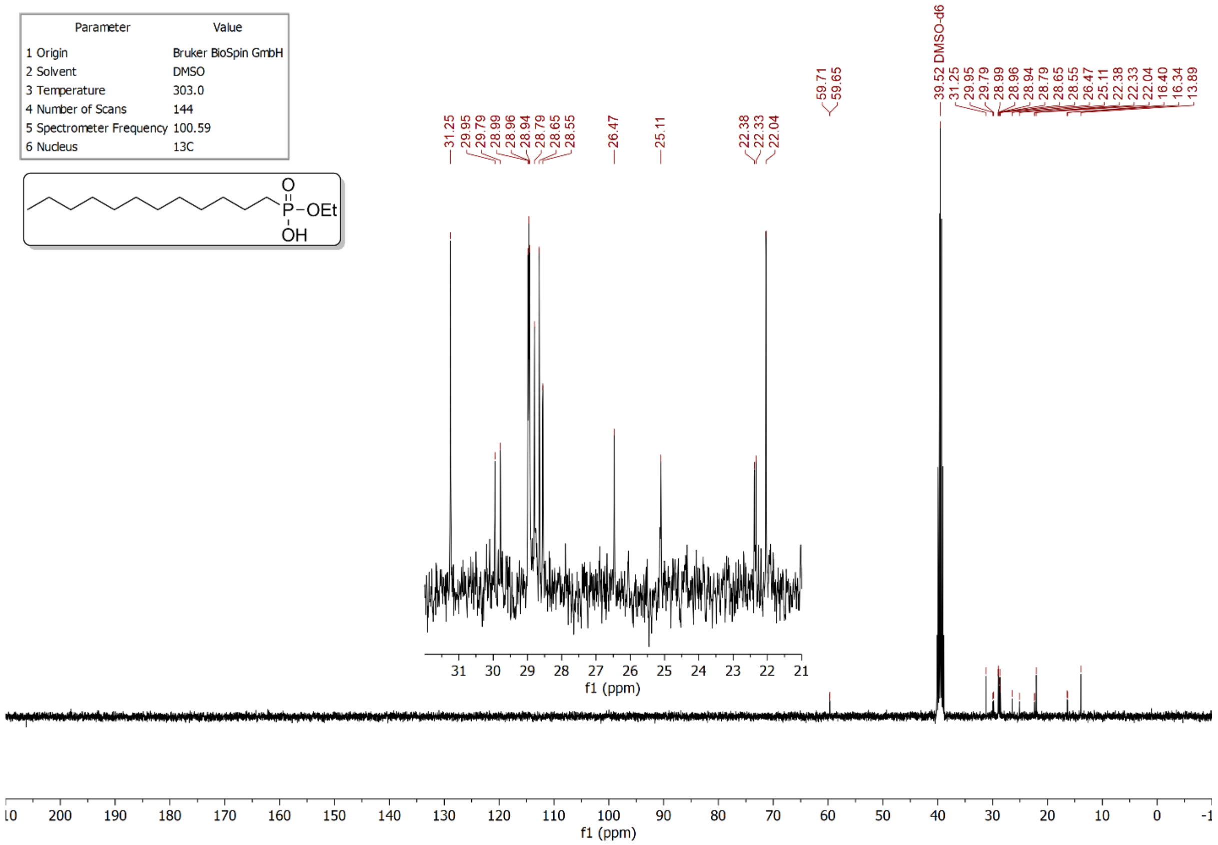

Parameter Value
1 Origin Bruker BioSpin GmbH
2 Solvent DMSO
3 Temperature 303.0
4 Number of Scans 144
5 Spectrometer Frequency 100.59
6 Nucleus 13C
O
P-OEt
OH
31.25
29.95
29.79
28.99
28.96
28.94
28.79
28.65
28.55
26.47
25.11
22.38
22.33
22.04
59.71
59.65
39.52 DMSO-d6
16.40
16.34
13.89
f1 (ppm)

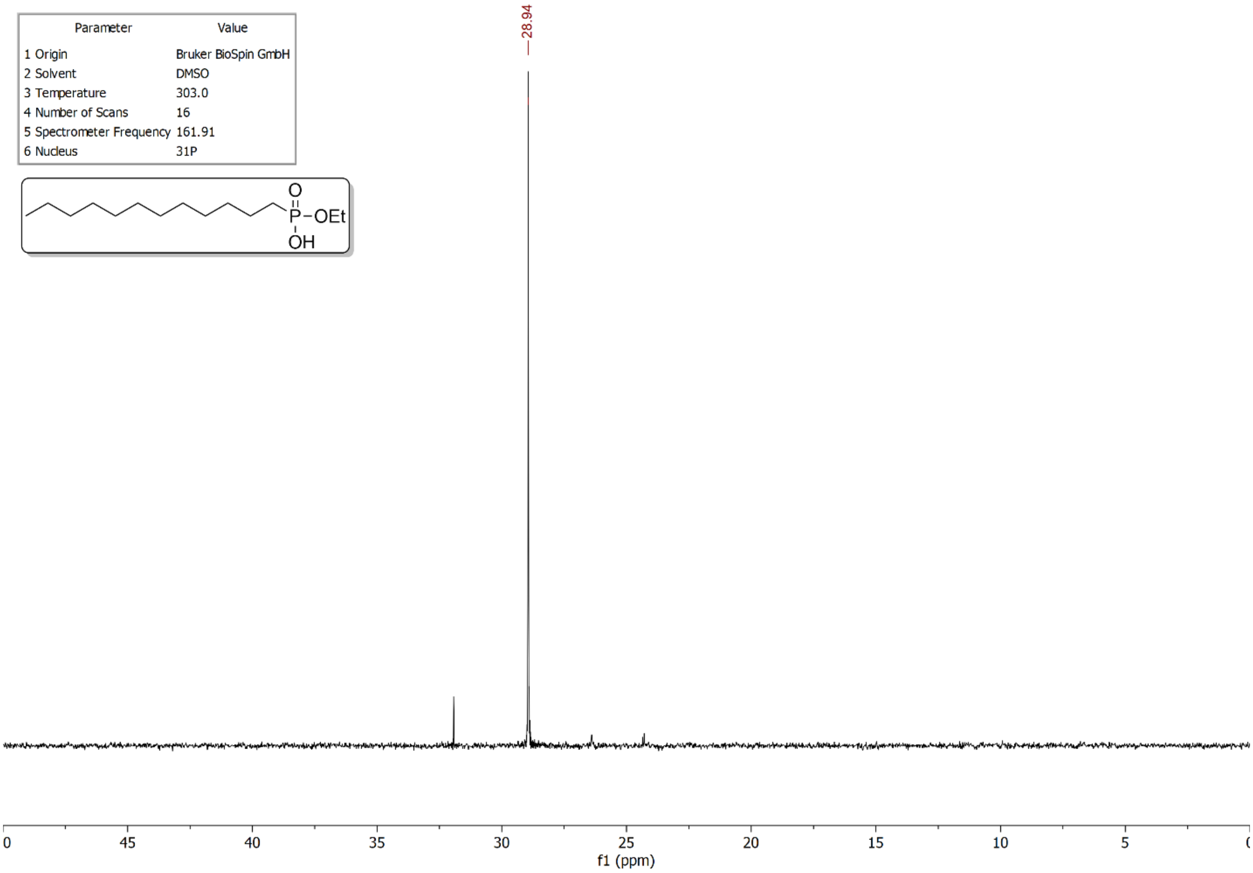

Parameter Value
1 Origin Bruker BioSpin GmbH
2 Solvent DMSO
3 Temperature 303.0
4 Number of Scans 16
5 Spectrometer Frequency 161.91
6 Nucleus 31P
O
P-OEt
OH
28.94
f1 (ppm)

Ethyl hydrogen phenylphosphonate (**3g**)

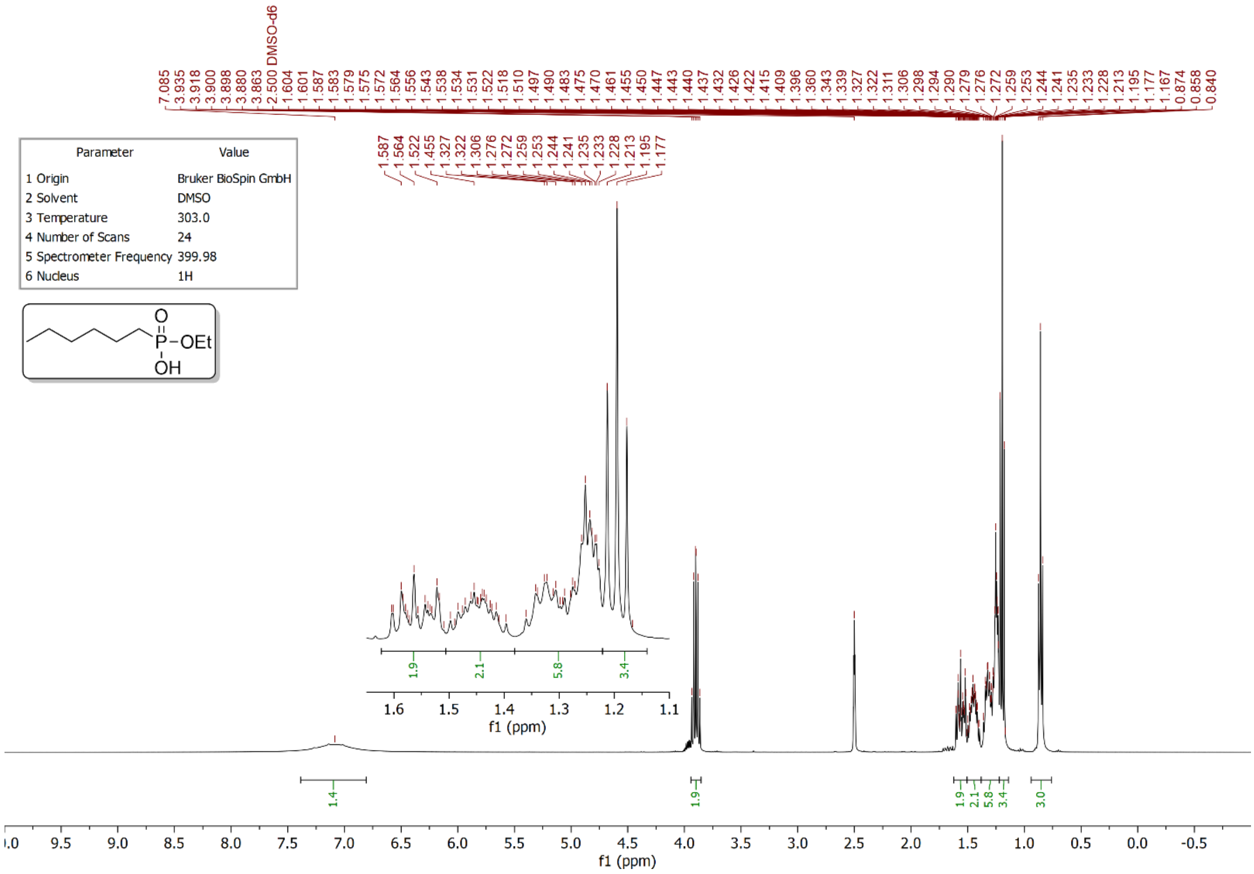

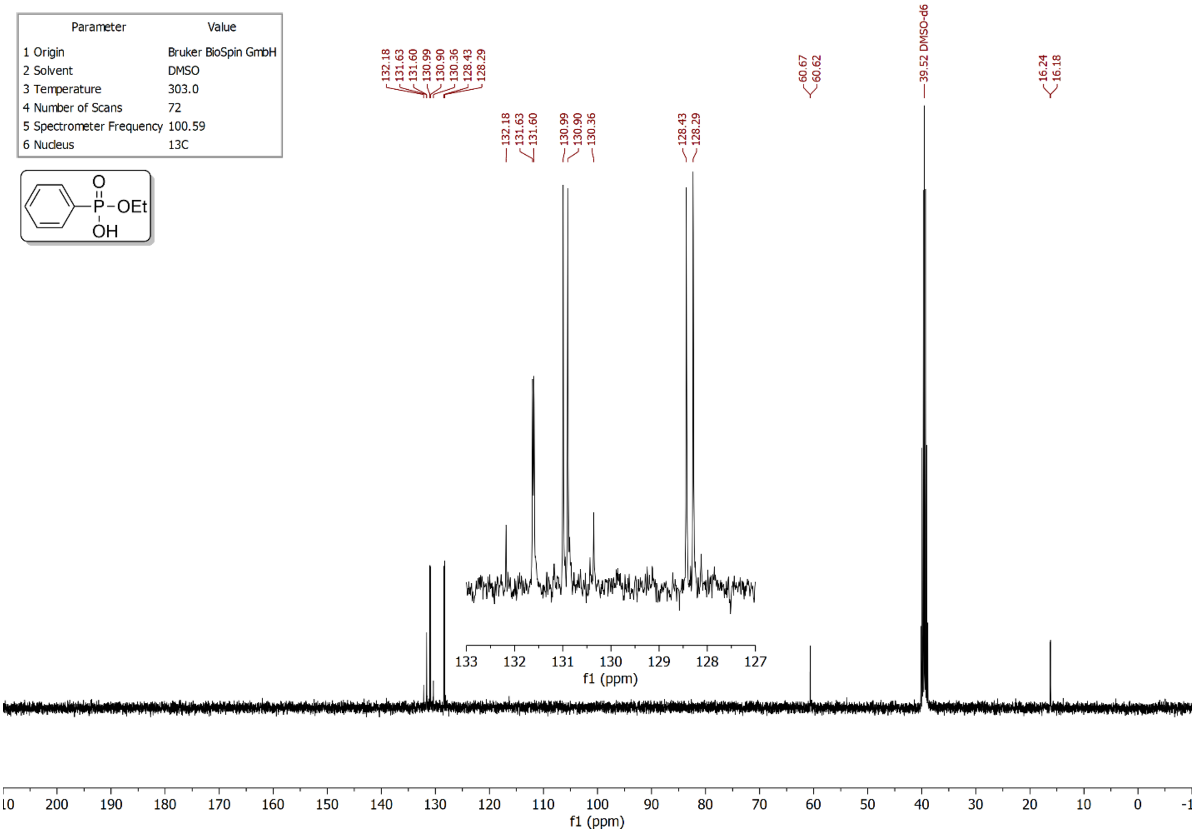
Parameter Value
1 Origin Bruker BioSpin GmbH
2 Solvent DMSO
3 Temperature 303.0
4 Number of Scans 72
5 Spectrometer Frequency 100.59
6 Nucleus 13C
P-OEt
OH
132.18
131.63
131.60
130.99
130.90
130.36
128.43
128.29
60.67
60.62
39.52 DMSO-d6
16.24
16.18
f1 (ppm)

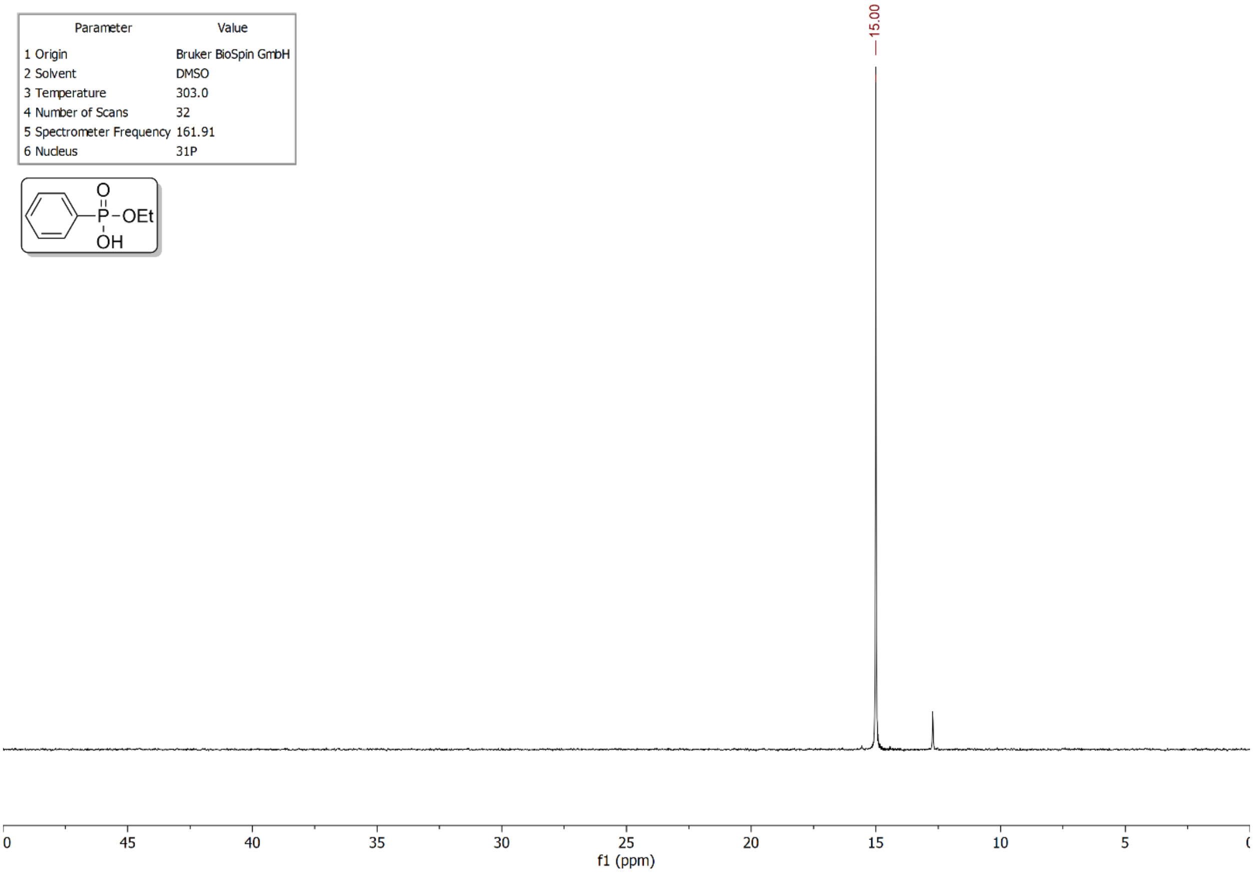
Parameter Value
1 Origin Bruker BioSpin GmbH
2 Solvent DMSO
3 Temperature 303.0
4 Number of Scans 32
5 Spectrometer Frequency 161.91
6 Nucleus 31P
P-OEt
OH
15.00
f1 (ppm)

Ethyl hydrogen (4-methoxyphenyl)phosphonate (**3h**)

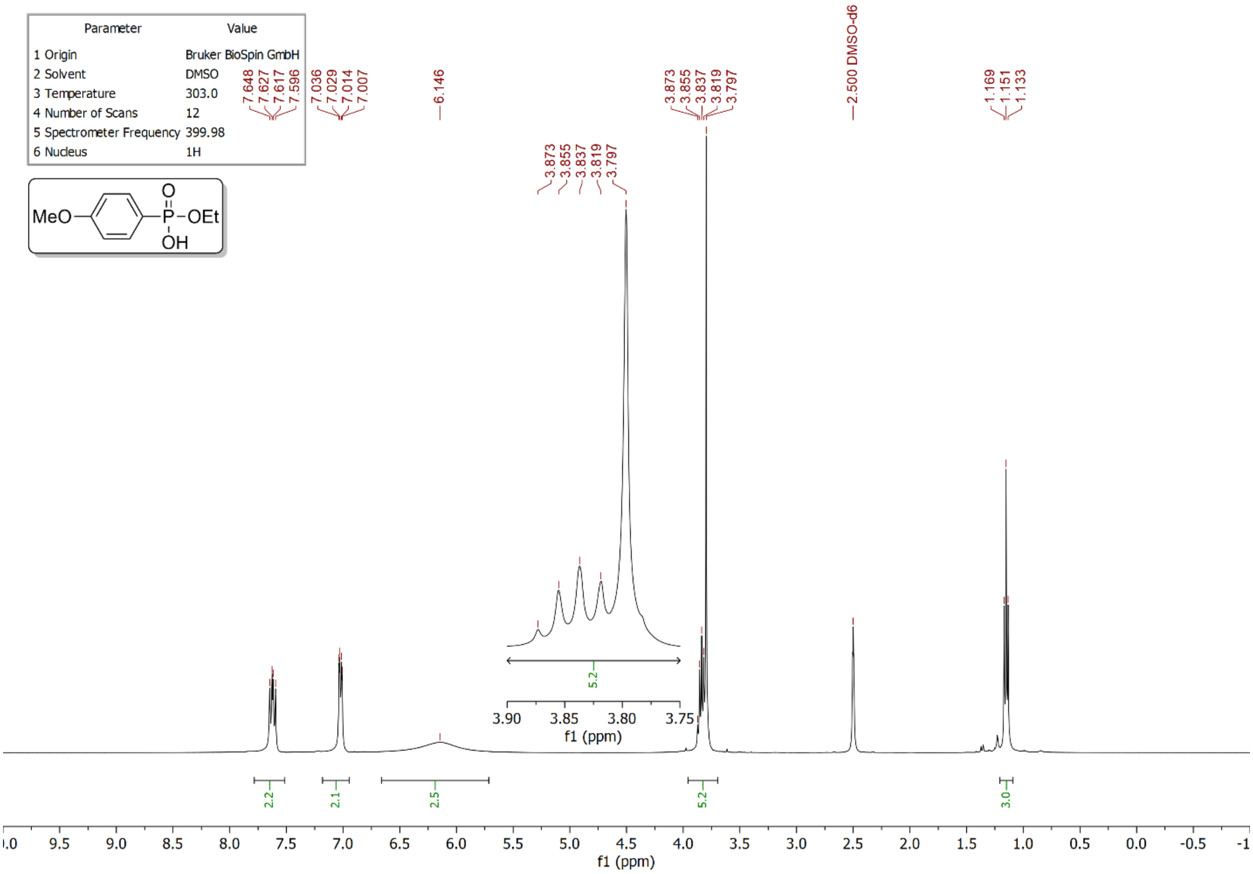

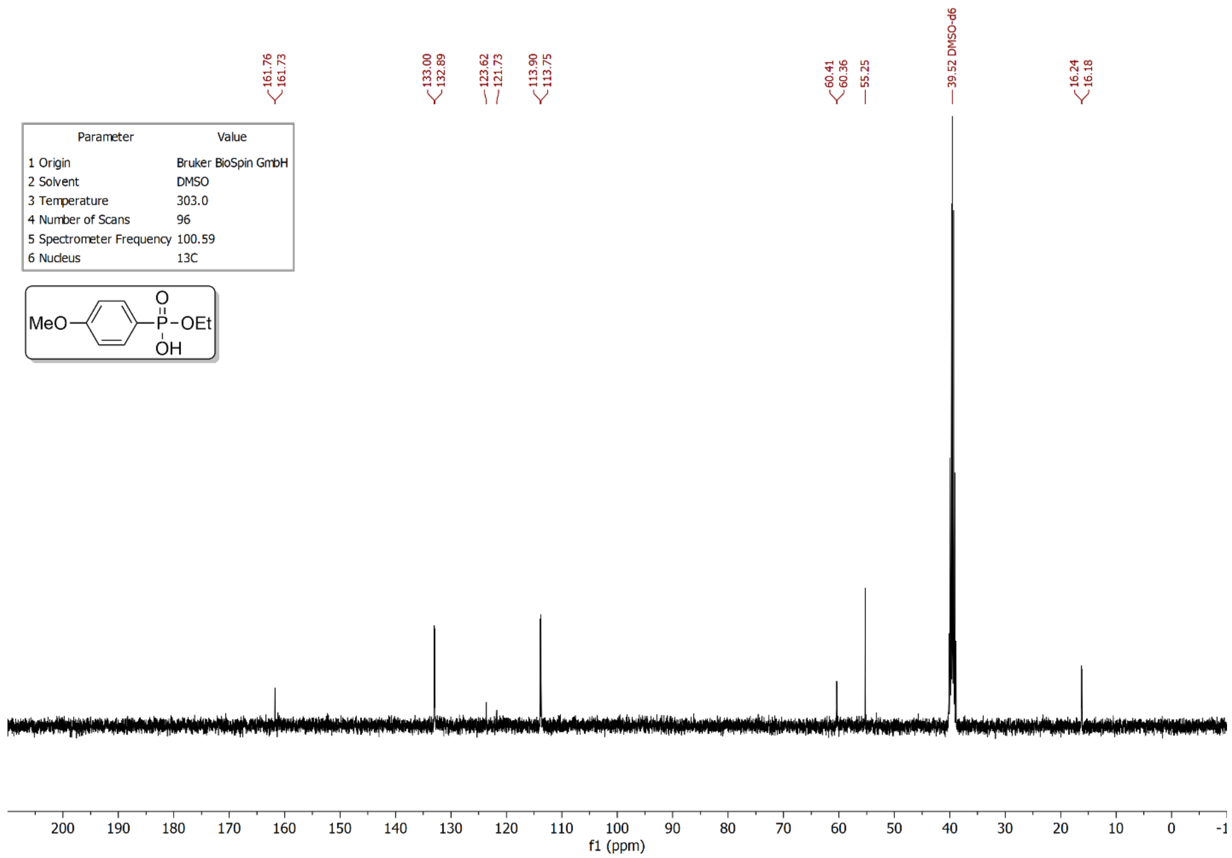
161.76
161.73
133.00
132.89
123.62
121.73
113.90
113.75
60.41
60.36
55.25
39.52 DMSO-d6
16.24
16.18
Parameter Value
1 Origin Bruker BioSpin GmbH
2 Solvent DMSO
3 Temperature 303.0
4 Number of Scans 96
5 Spectrometer Frequency 100.59
6 Nucleus 13C
MeO
O
P
OEt
OH
f1 (ppm)

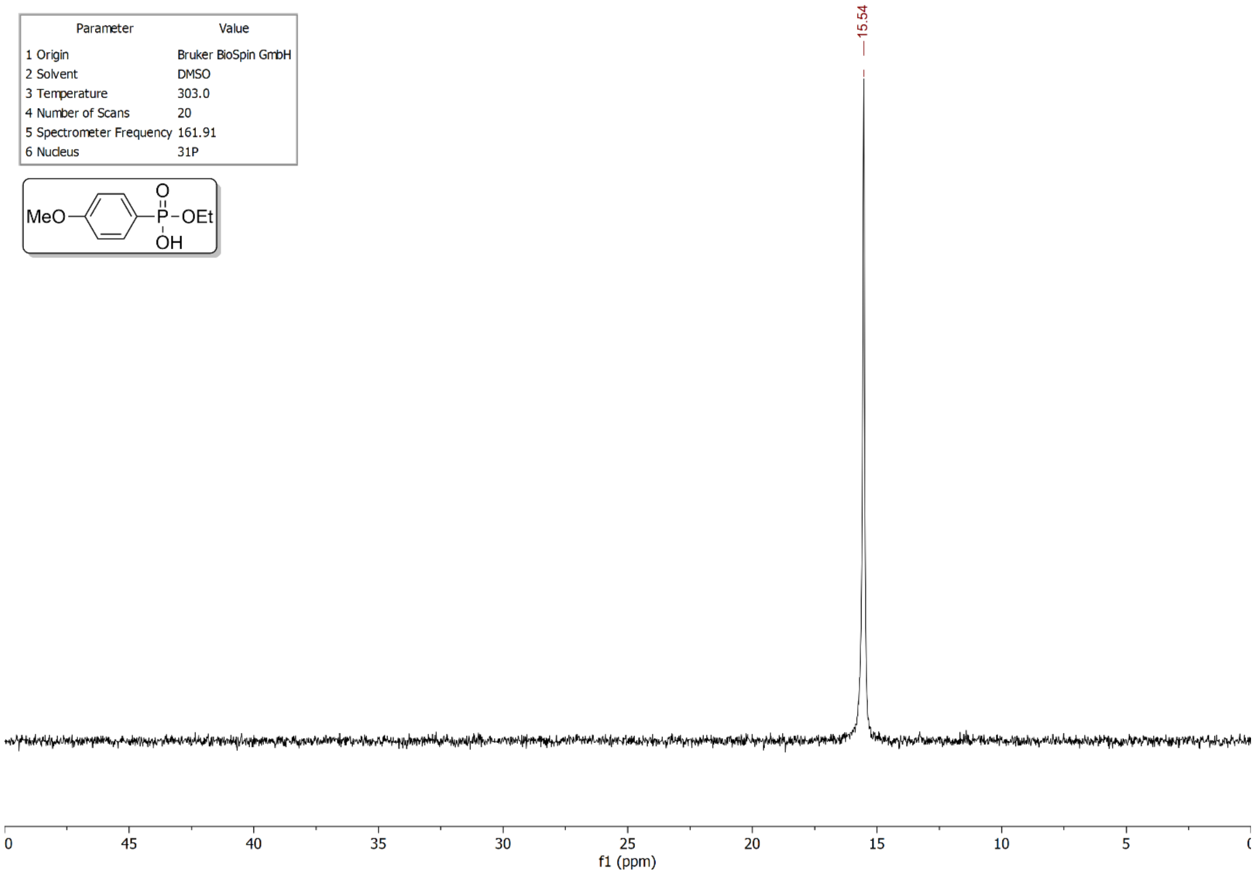
15.54
Parameter Value
1 Origin Bruker BioSpin GmbH
2 Solvent DMSO
3 Temperature 303.0
4 Number of Scans 20
5 Spectrometer Frequency 161.91
6 Nucleus 31P
MeO
O
P
OEt
OH
f1 (ppm)

Ethyl hydrogen [(4-nitrophenyl)methyl]phosphonate (**3j**)

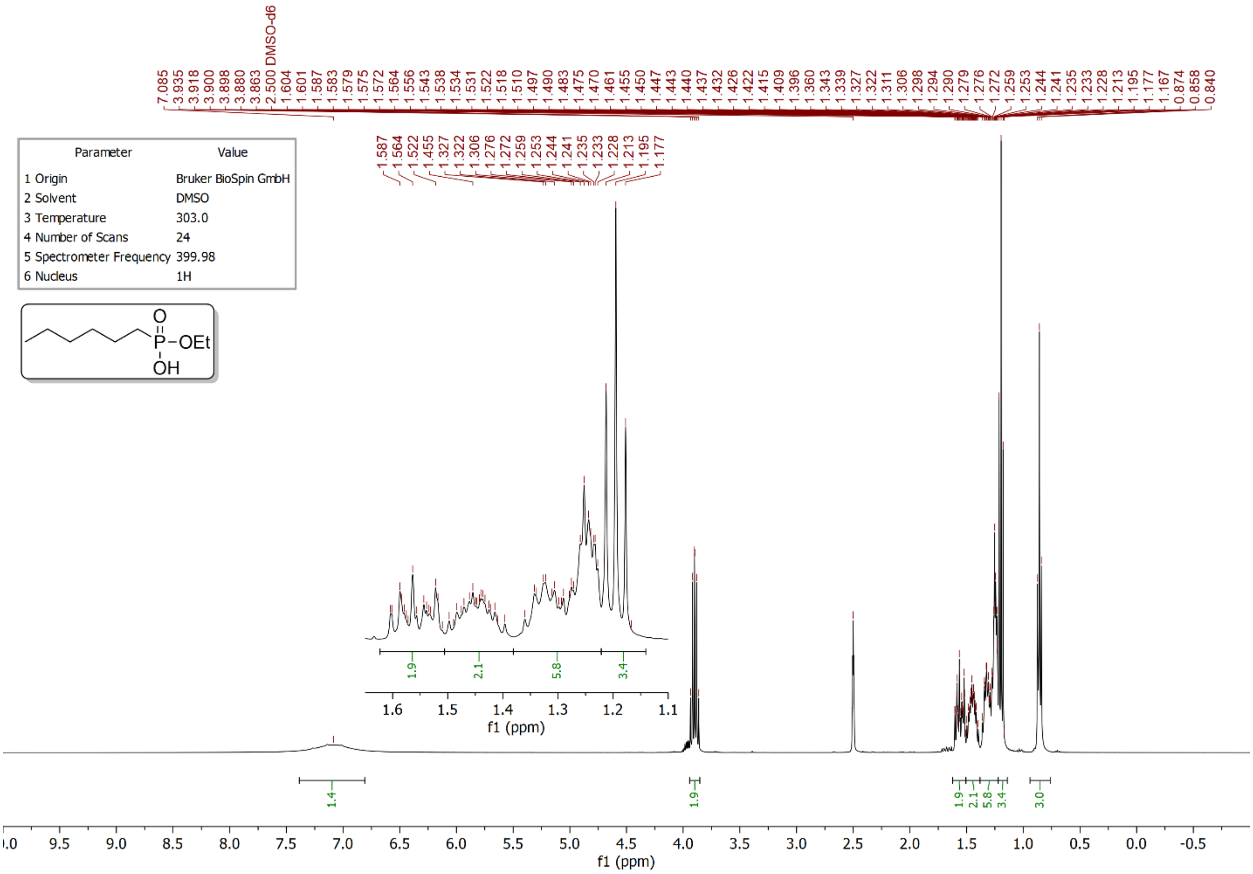

Parameter	Value
1 Origin	Bruker BioSpin GmbH
2 Solvent	DMSO
3 Temperature	303.0
4 Number of Scans	24
5 Spectrometer Frequency	399.98
6 Nucleus	1H

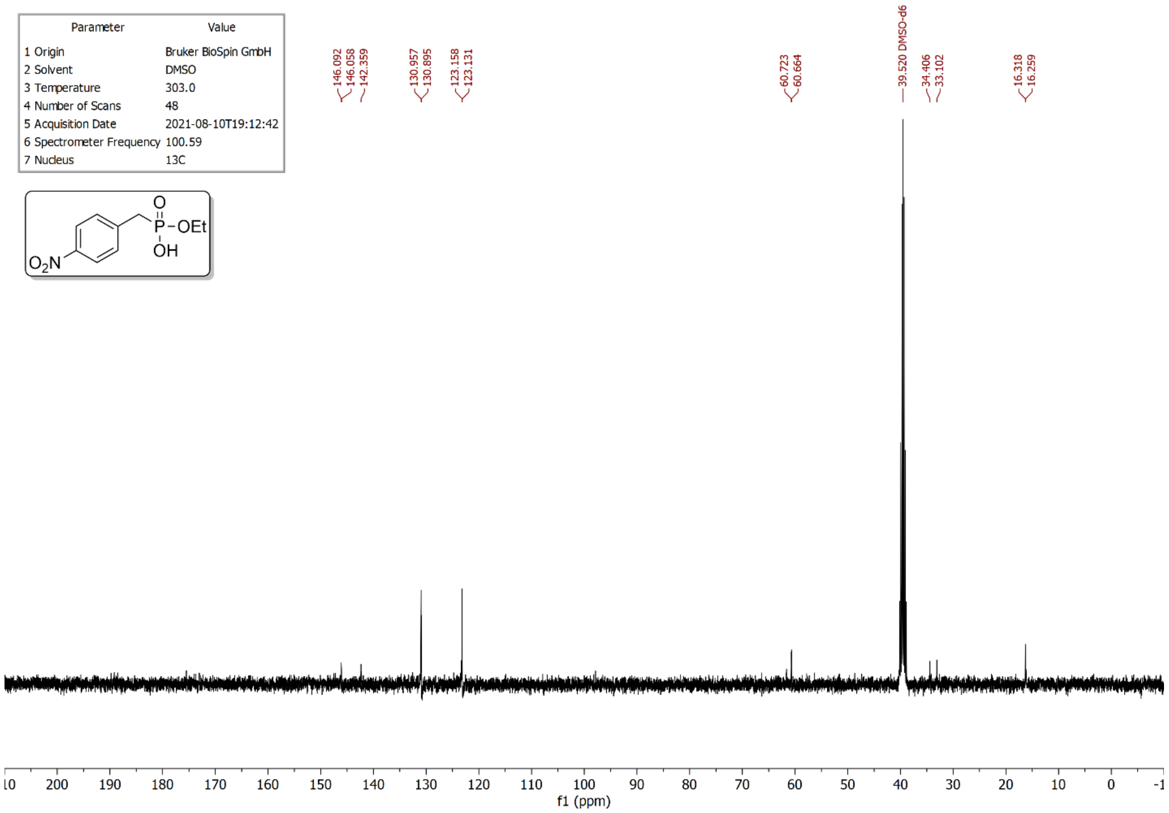
Parameter Value
1 Origin Bruker BioSpin GmbH
2 Solvent DMSO
3 Temperature 303.0
4 Number of Scans 48
5 Acquisition Date 2021-08-10T19:12:42
6 Spectrometer Frequency 100.59
7 Nucleus 13C
O
P-OEt
OH
O2N
146.092
146.058
142.359
130.957
130.895
123.158
123.131
60.723
60.664
39.520 DMSO-d6
34.406
33.102
16.318
16.259
f1 (ppm)

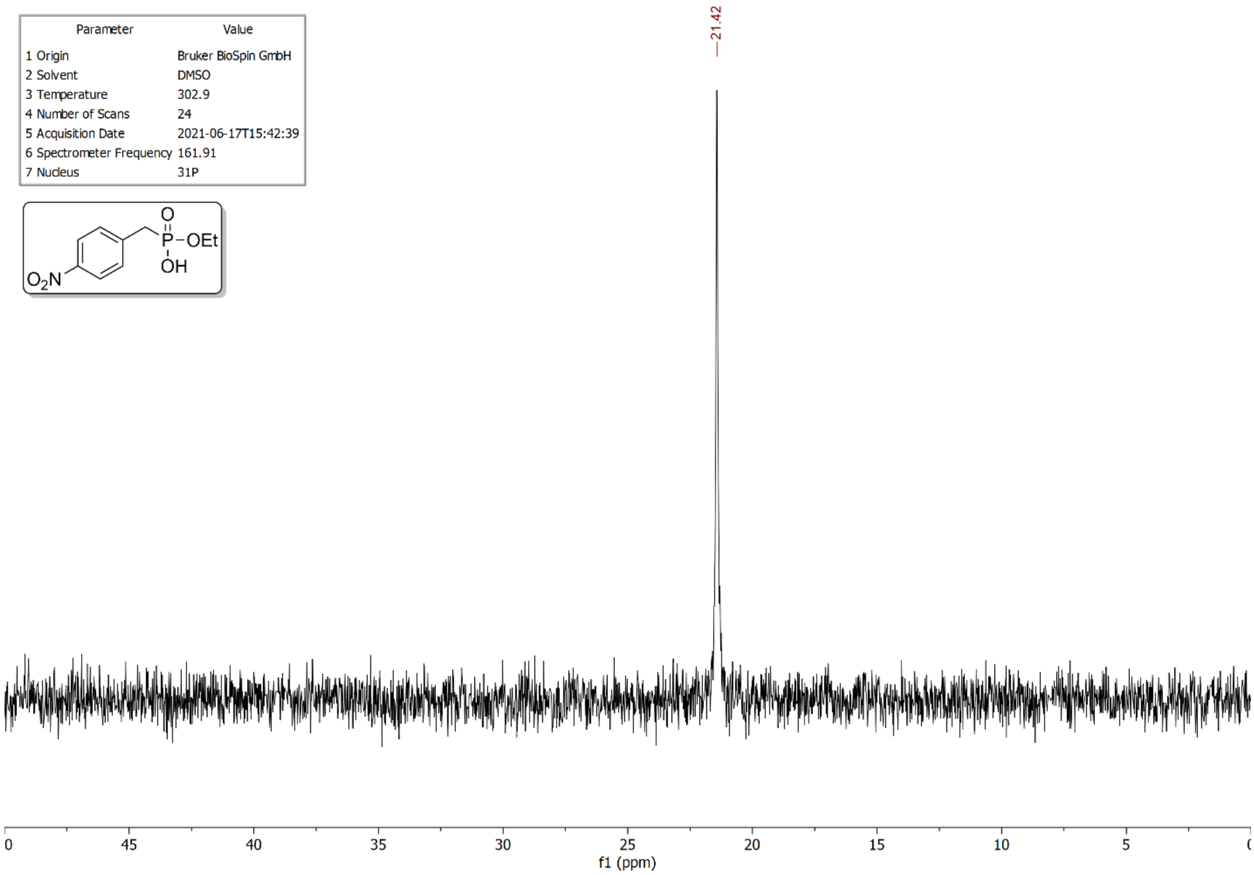
Parameter Value
1 Origin Bruker BioSpin GmbH
2 Solvent DMSO
3 Temperature 302.9
4 Number of Scans 24
5 Acquisition Date 2021-06-17T15:42:39
6 Spectrometer Frequency 161.91
7 Nucleus 31P
O
P-OEt
OH
O2N
21.42
f1 (ppm)

Ethyl hydrogen [(4-bromophenyl)methyl]phosphonate (**3k**)

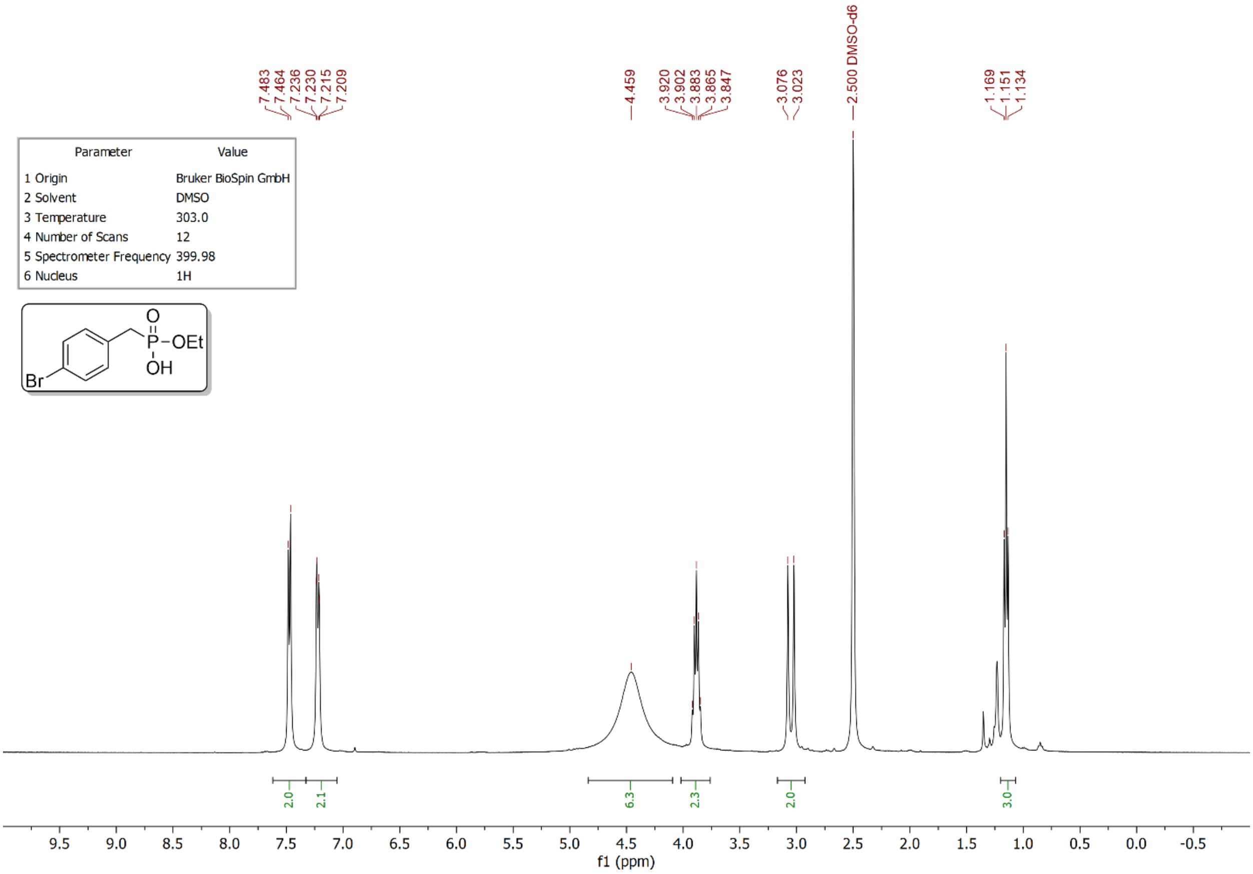

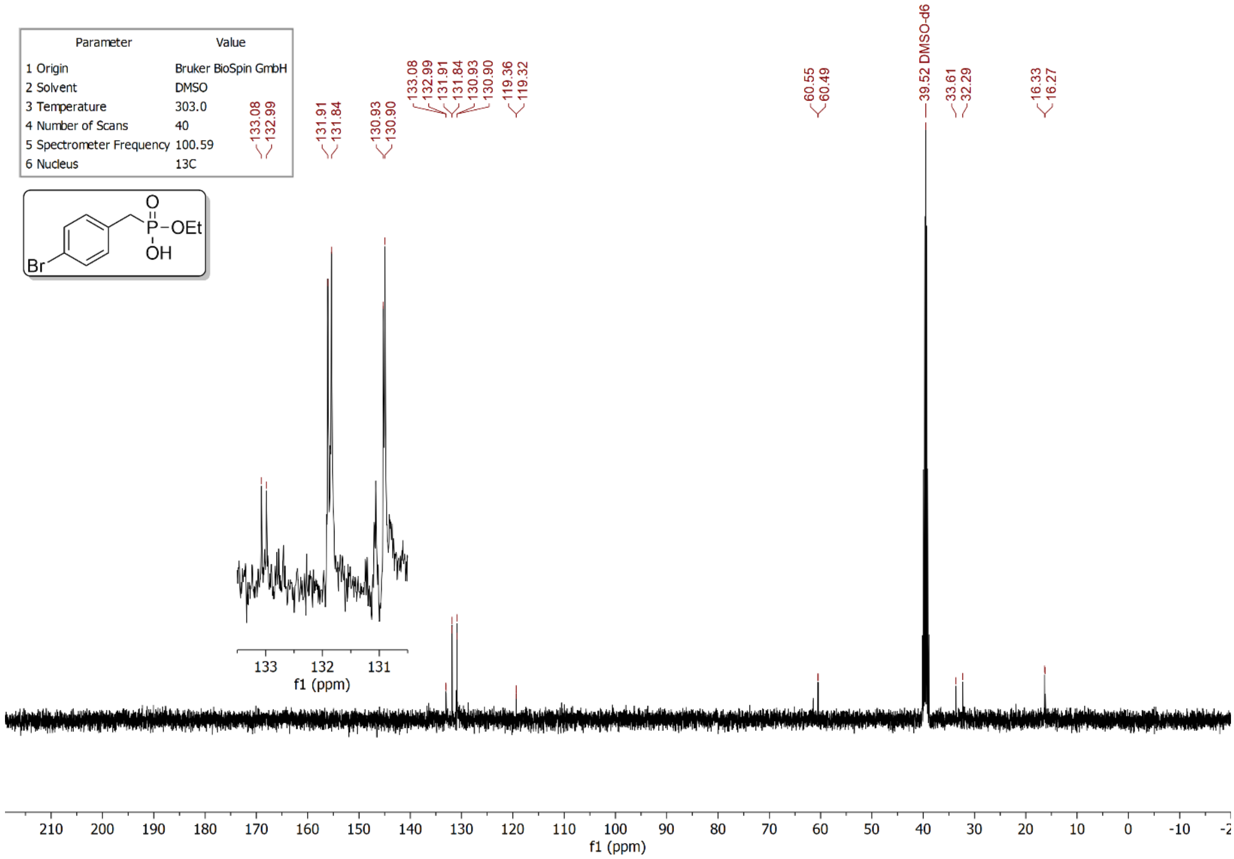
Parameter Value
1 Origin Bruker BioSpin GmbH
2 Solvent DMSO
3 Temperature 303.0
4 Number of Scans 40
5 Spectrometer Frequency 100.59
6 Nucleus 13C
133.08
132.99
131.91
131.84
130.93
130.90
119.36
119.32
60.55
60.49
39.52 DMSO-d6
33.61
32.29
16.33
16.27
O
P-OEt
OH
Br
f1 (ppm)

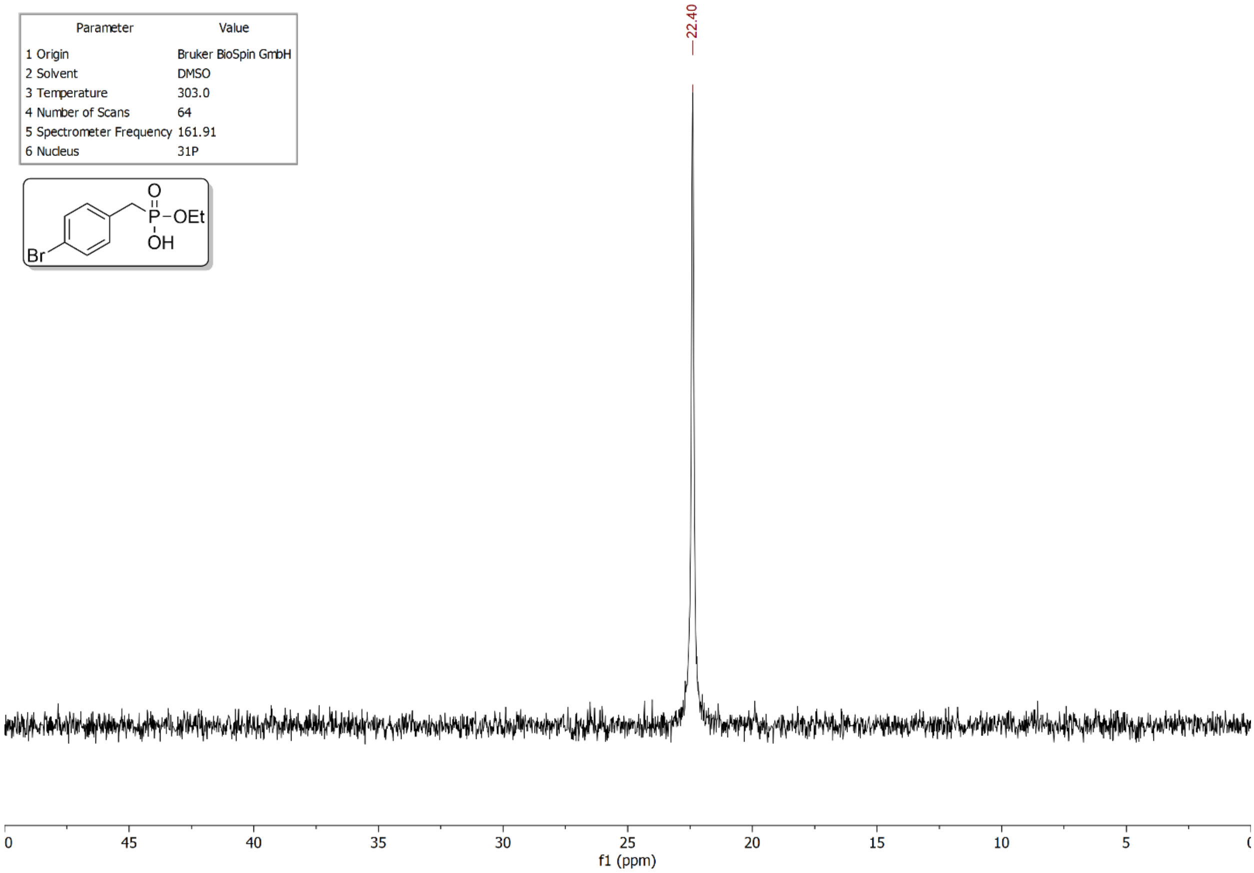
Parameter Value
1 Origin Bruker BioSpin GmbH
2 Solvent DMSO
3 Temperature 303.0
4 Number of Scans 64
5 Spectrometer Frequency 161.91
6 Nucleus 31P
22.40
O
P-OEt
OH
Br
f1 (ppm)

Ethyl hydrogen [(3-bromophenyl)methyl]phosphonate (**3l**)

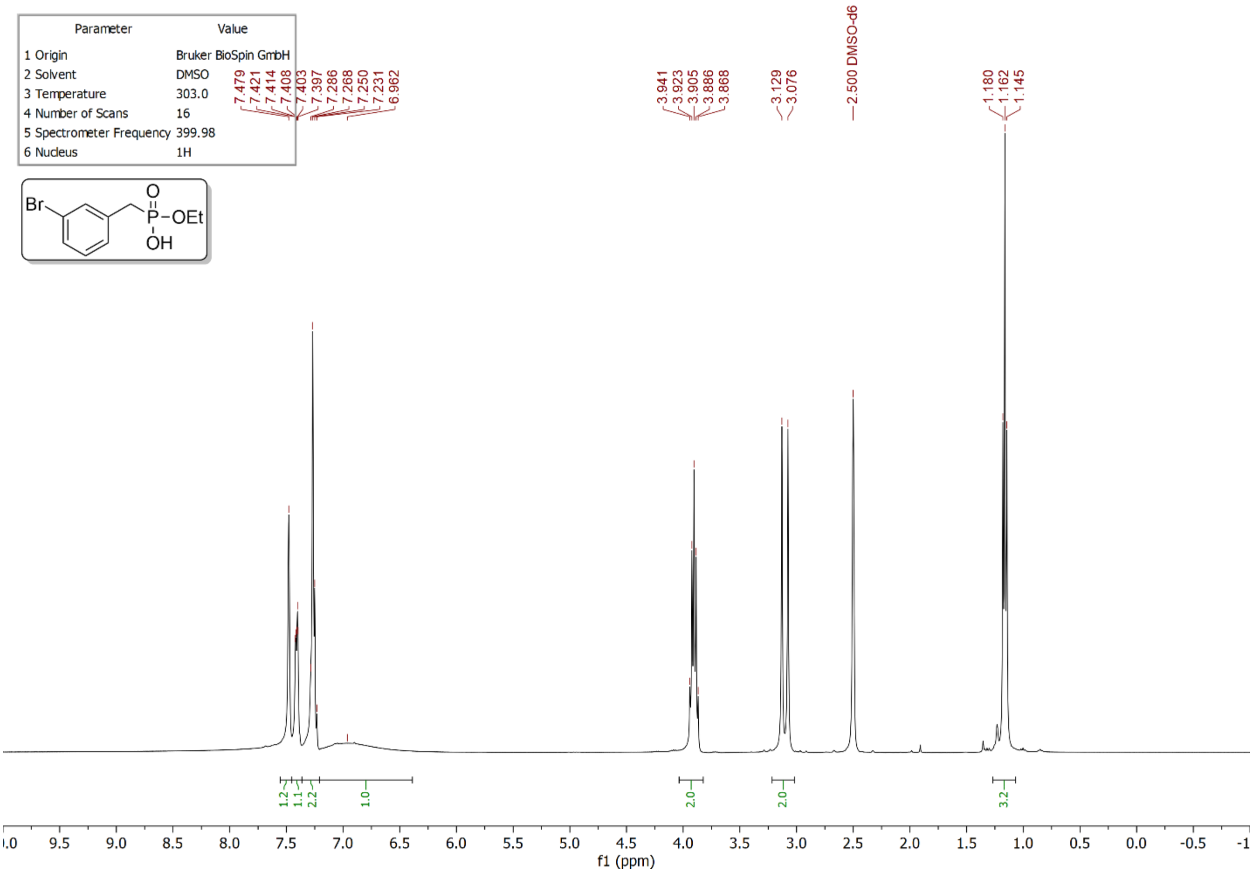

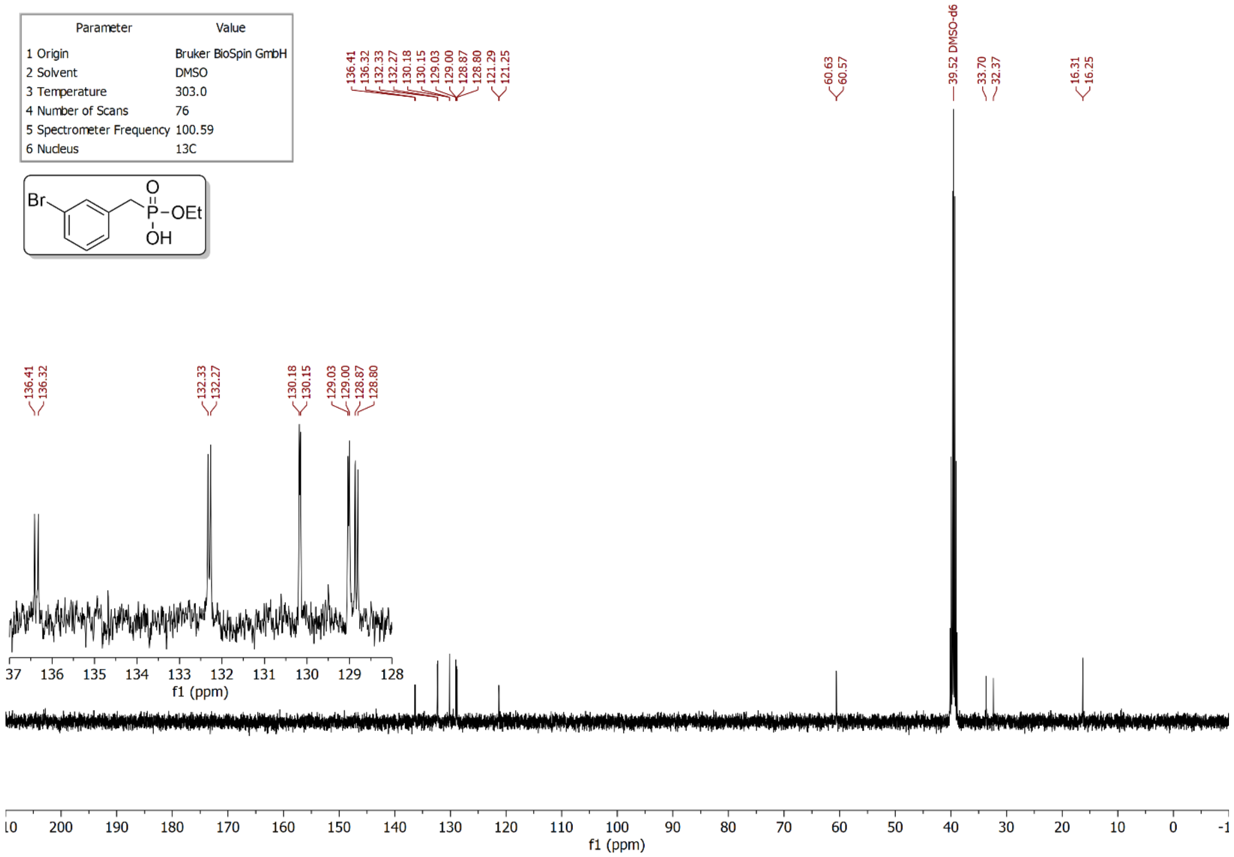

Parameter Value
1 Origin Bruker BioSpin GmbH
2 Solvent DMSO
3 Temperature 303.0
4 Number of Scans 76
5 Spectrometer Frequency 100.59
6 Nucleus 13C
Br
O
P–OEt
OH
136.41 136.32 132.33 132.27 130.18 130.15 129.03 129.00 128.87 128.80 121.29 121.25
60.63 60.57
39.52 DMSO-d6
33.70 32.37
16.31 16.25
f1 (ppm)

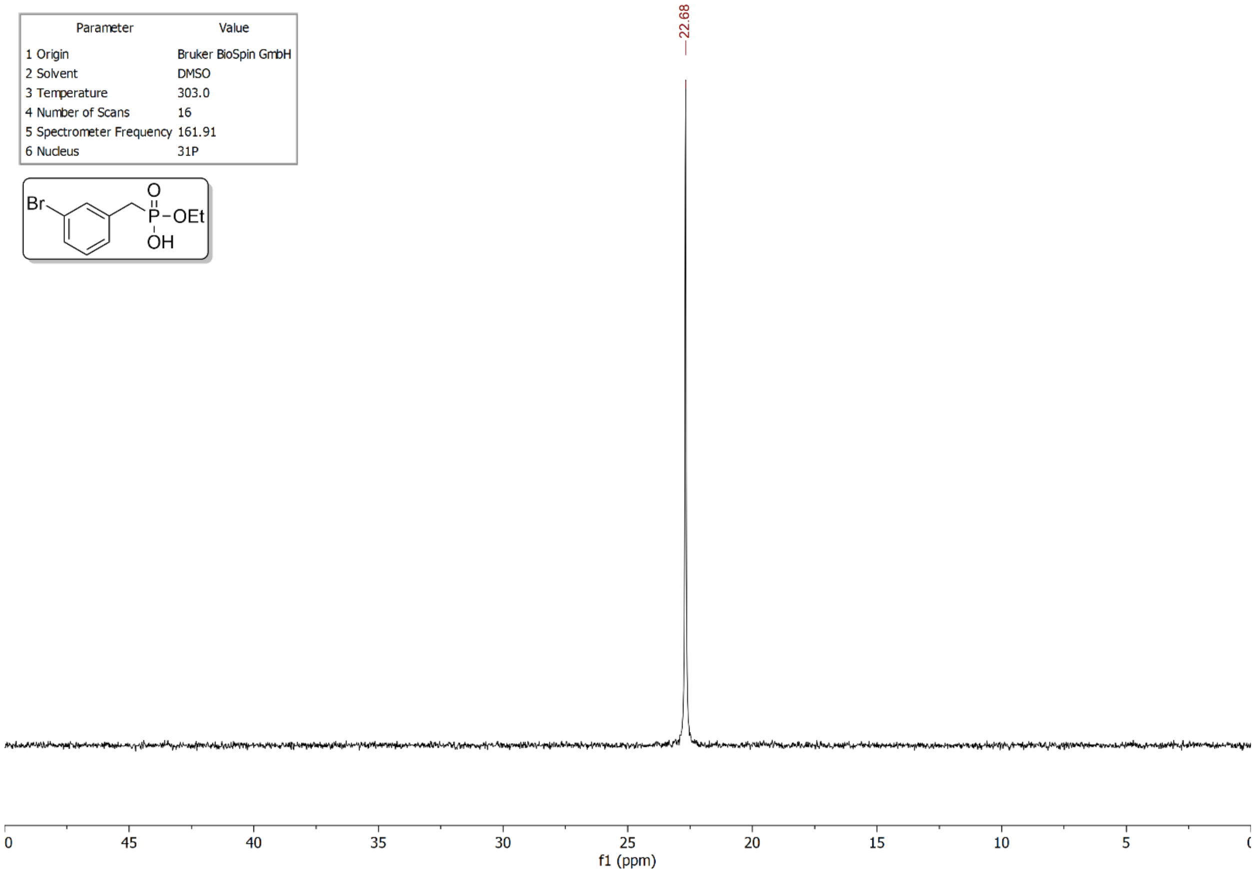

22.68
Parameter Value
1 Origin Bruker BioSpin GmbH
2 Solvent DMSO
3 Temperature 303.0
4 Number of Scans 16
5 Spectrometer Frequency 161.91
6 Nucleus 31P
Br
O
P–OEt
OH
f1 (ppm)

2.4. *^{1}H, ^{13}C and ^{31}P NMR spectra of the diesters*

Diethyl butylphosphonate (**4a**)

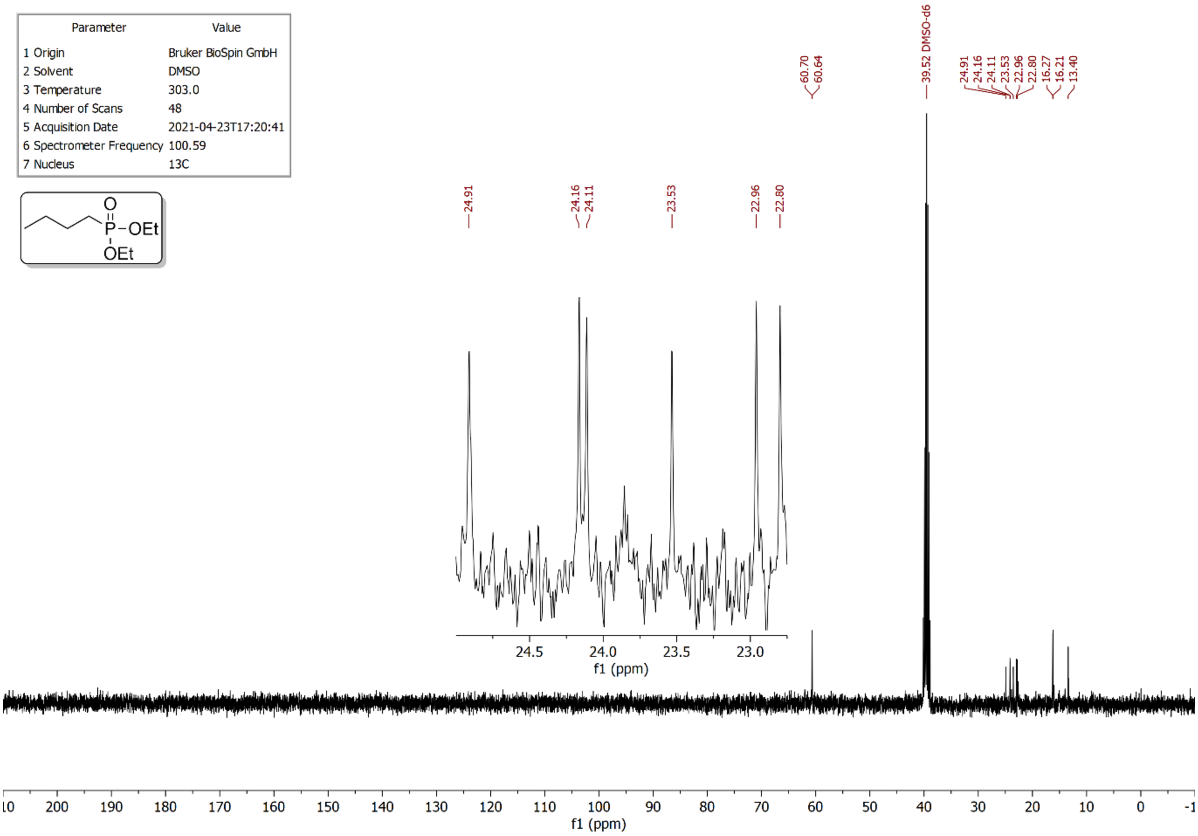

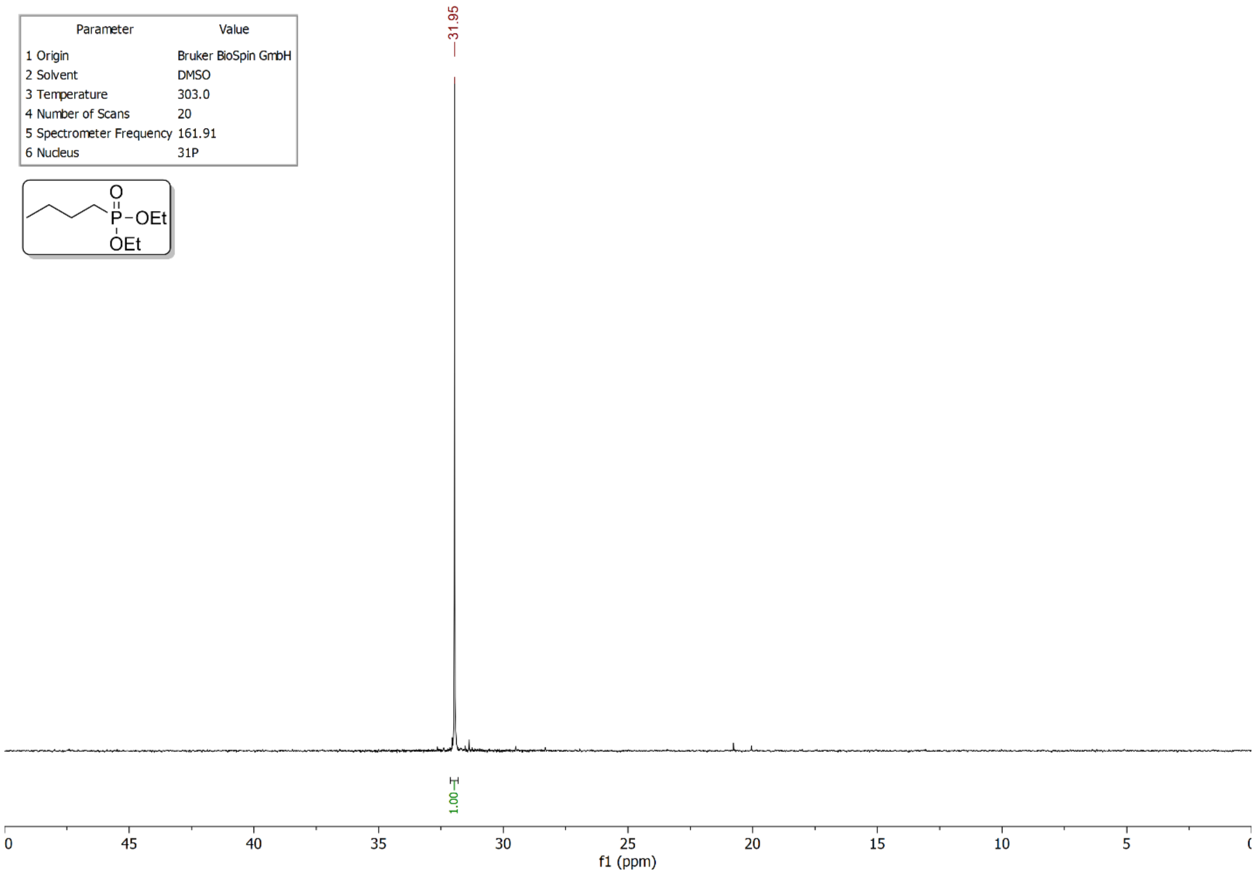

Parameter Value
1 Origin Bruker BioSpin GmbH
2 Solvent DMSO
3 Temperature 303.0
4 Number of Scans 20
5 Spectrometer Frequency 161.91
6 Nucleus 31P
O
P−OEt
OEt
31.95
1.00
0
45
40
35
30
25
20
15
10
5
f1 (ppm)

Diethyl benzylphosphonate (**4b**)

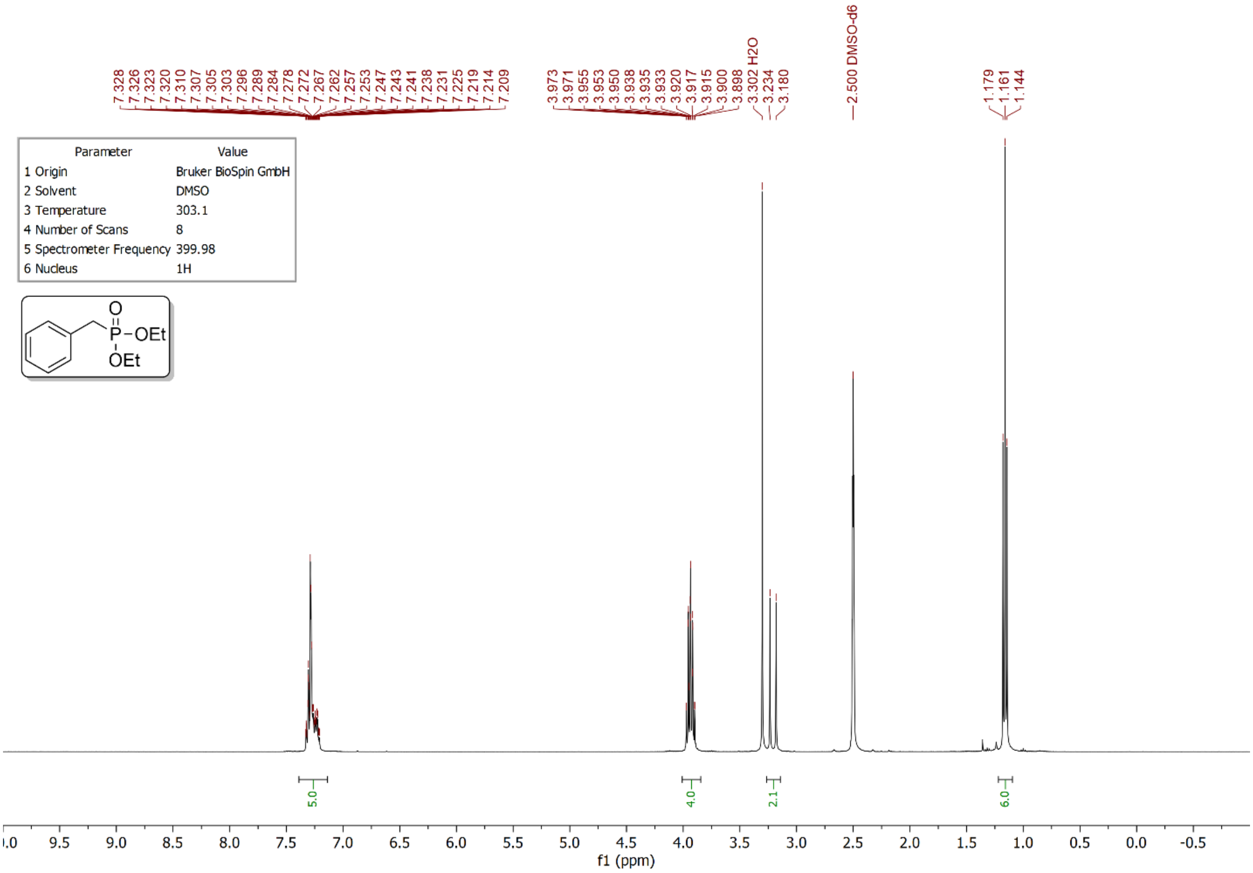

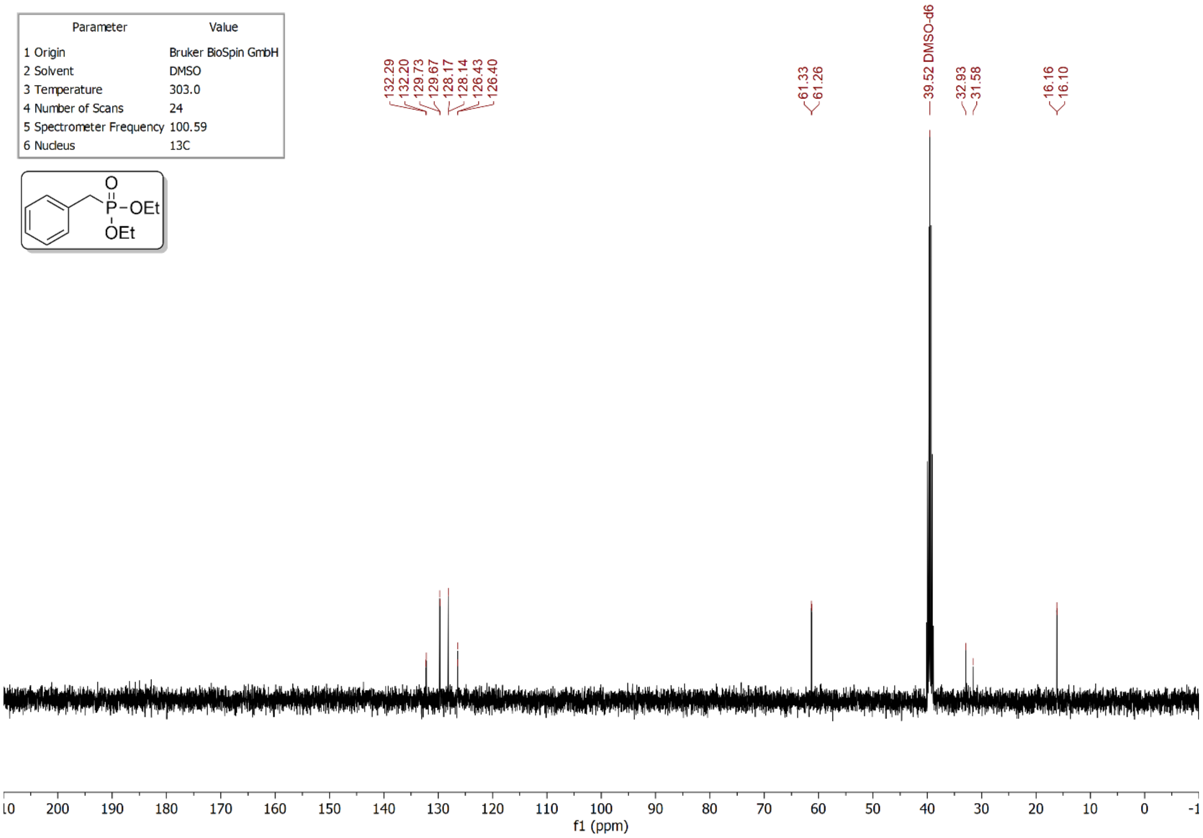
Parameter Value
1 Origin Bruker BioSpin GmbH
2 Solvent DMSO
3 Temperature 303.0
4 Number of Scans 24
5 Spectrometer Frequency 100.59
6 Nucleus 13C
P-OEt
OEt
132.29
132.20
129.73
129.67
128.17
128.14
126.43
126.40
61.33
61.26
39.52 DMSO-d6
32.93
31.58
16.16
16.10
f1 (ppm)

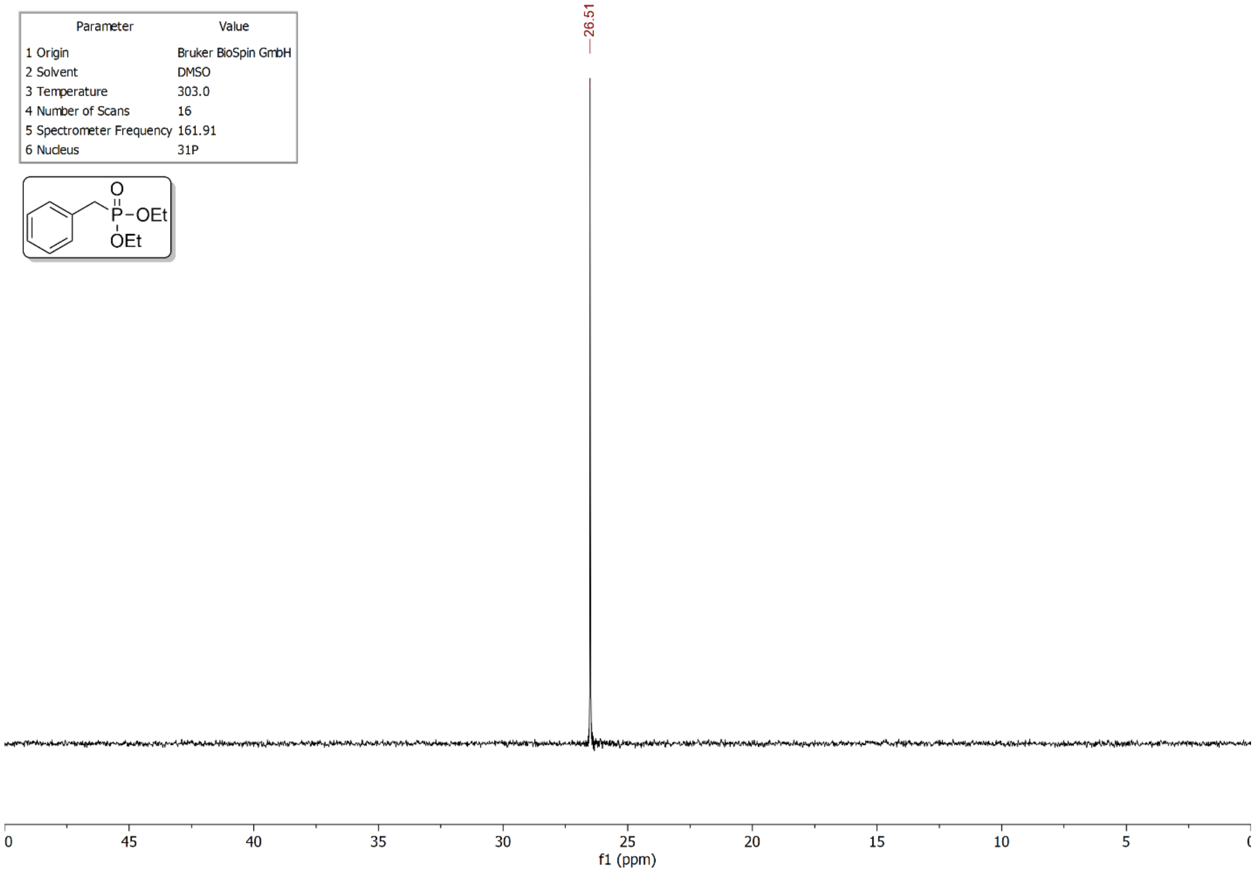
Parameter Value
1 Origin Bruker BioSpin GmbH
2 Solvent DMSO
3 Temperature 303.0
4 Number of Scans 16
5 Spectrometer Frequency 161.91
6 Nucleus 31P
P-OEt
OEt
26.51
f1 (ppm)

Diethyl ethylphosphonate (**4c**)

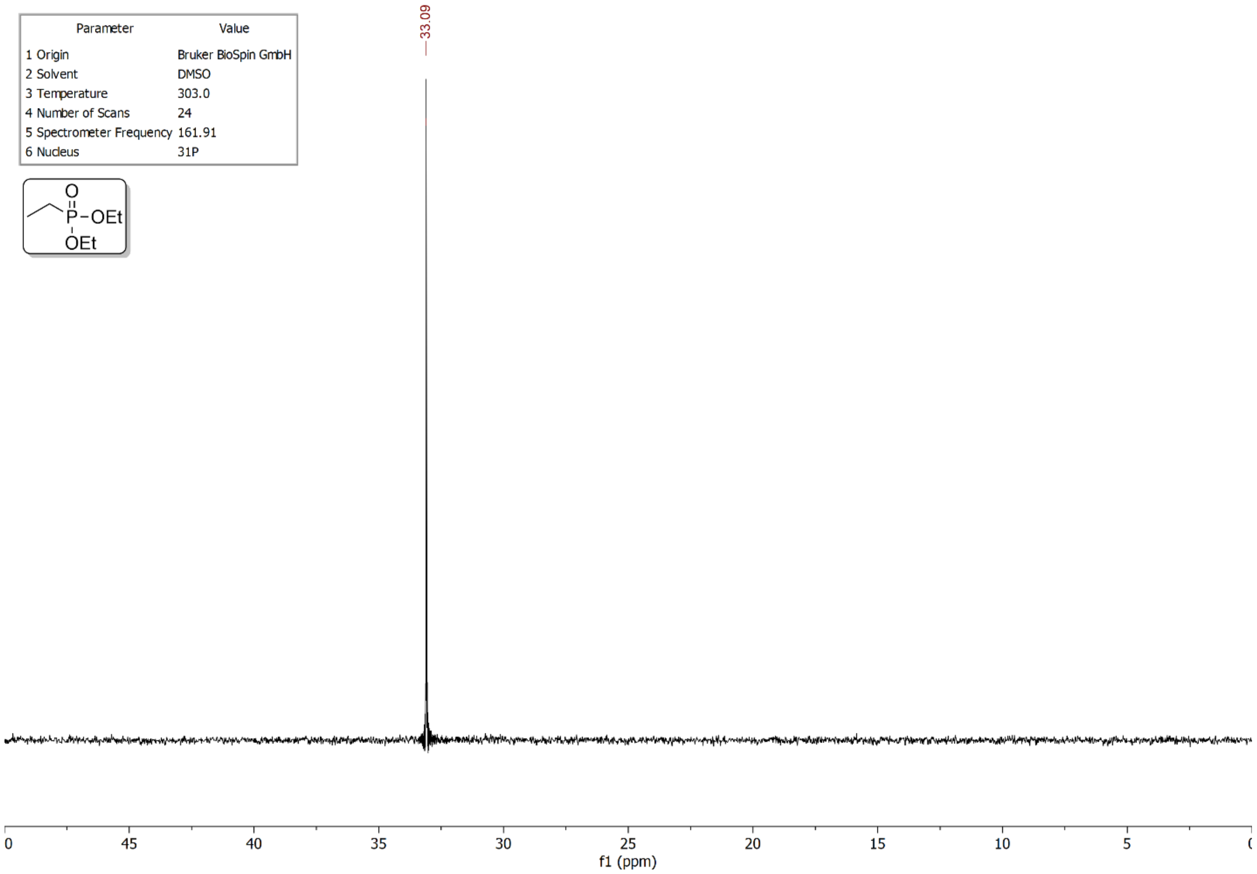

Diethyl hexylphosphonate (**4e**)

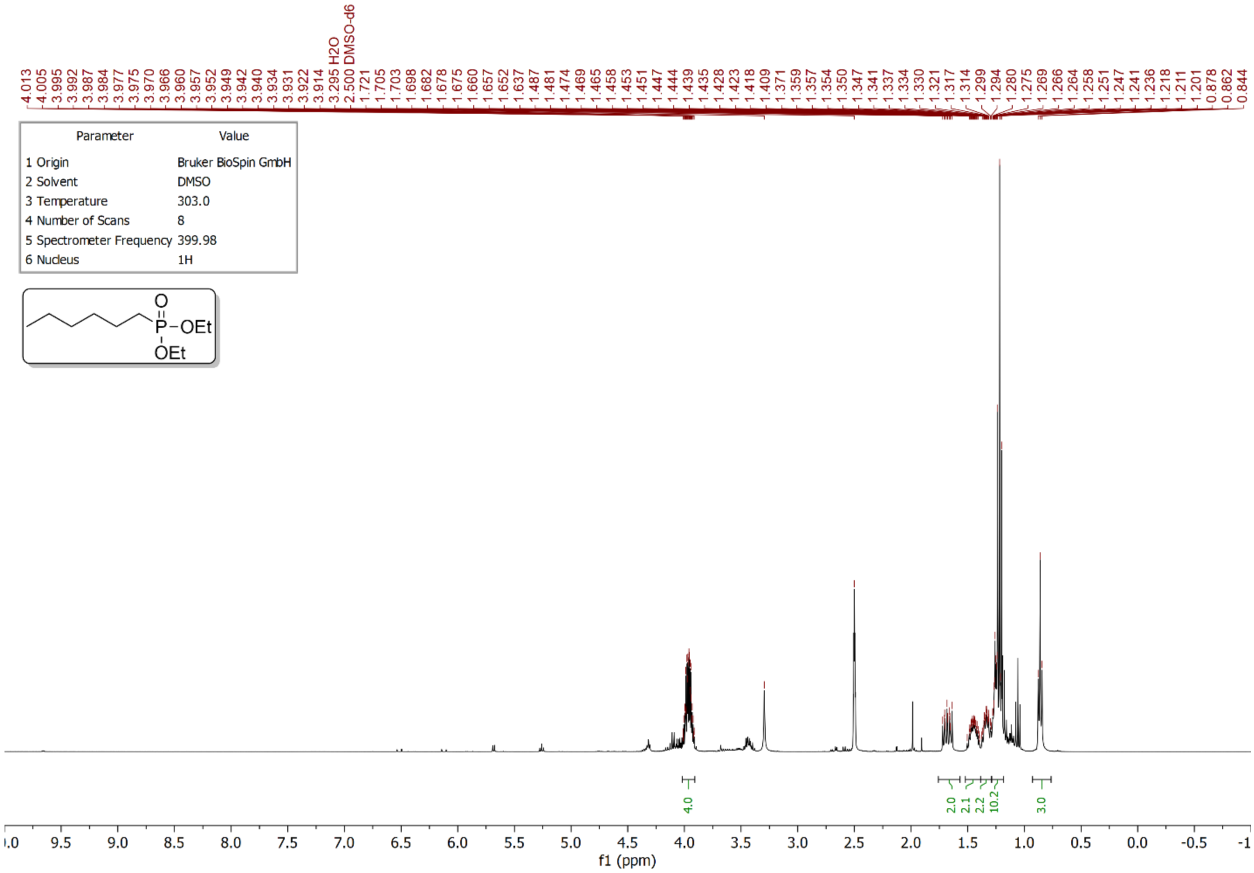

Parameter Value
1 Origin Bruker BioSpin GmbH
2 Solvent DMSO
3 Temperature 303.0
4 Number of Scans 8
5 Spectrometer Frequency 399.98
6 Nucleus 1H
P-OEt
OEt
3.295 H2O
2.500 DMSO-d6
f1 (ppm)

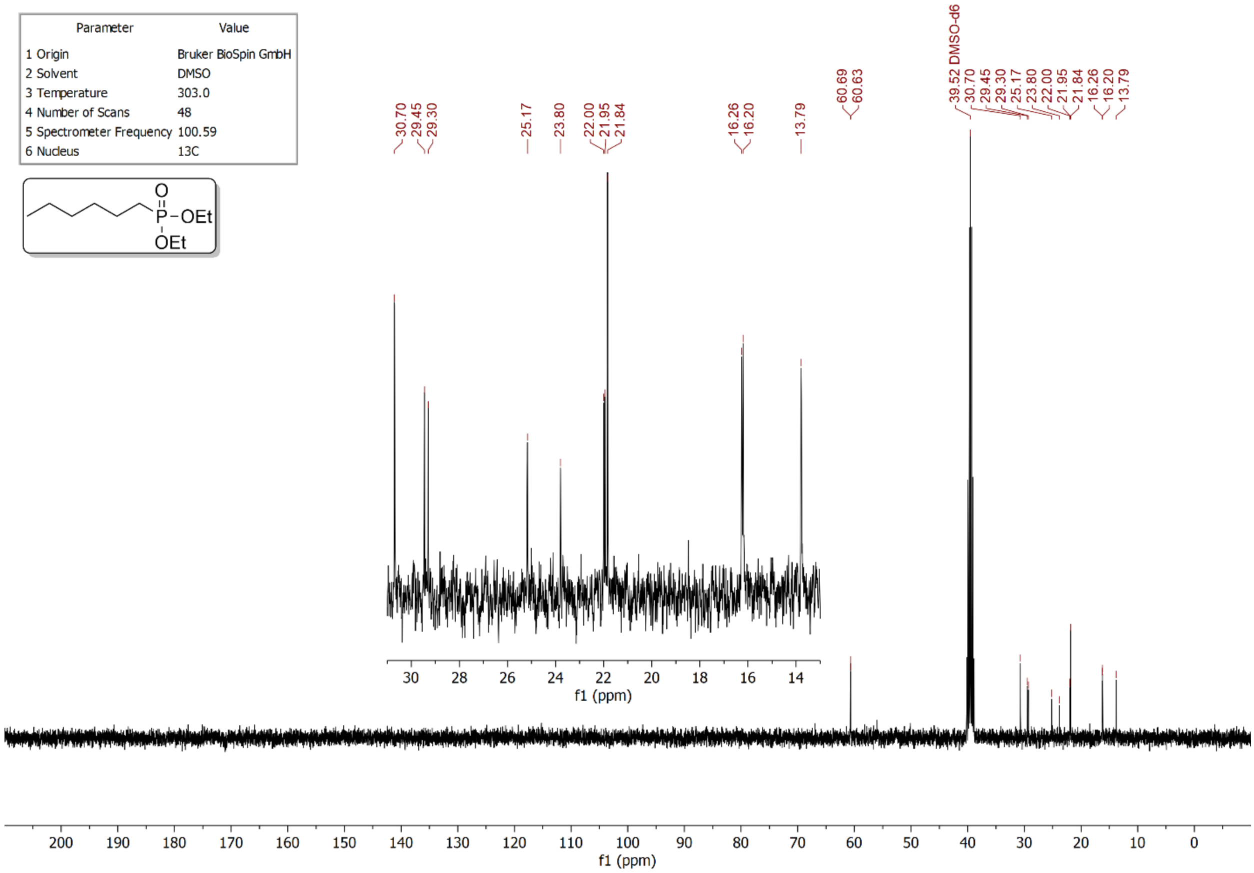

Parameter Value
1 Origin Bruker BioSpin GmbH
2 Solvent DMSO
3 Temperature 303.0
4 Number of Scans 48
5 Spectrometer Frequency 100.59
6 Nucleus 13C
O
P–OEt
OEt
30.70
29.45
29.30
25.17
23.80
22.00
21.95
21.84
16.26
16.20
13.79
60.69
60.63
39.52 DMSO-d6
f1 (ppm)

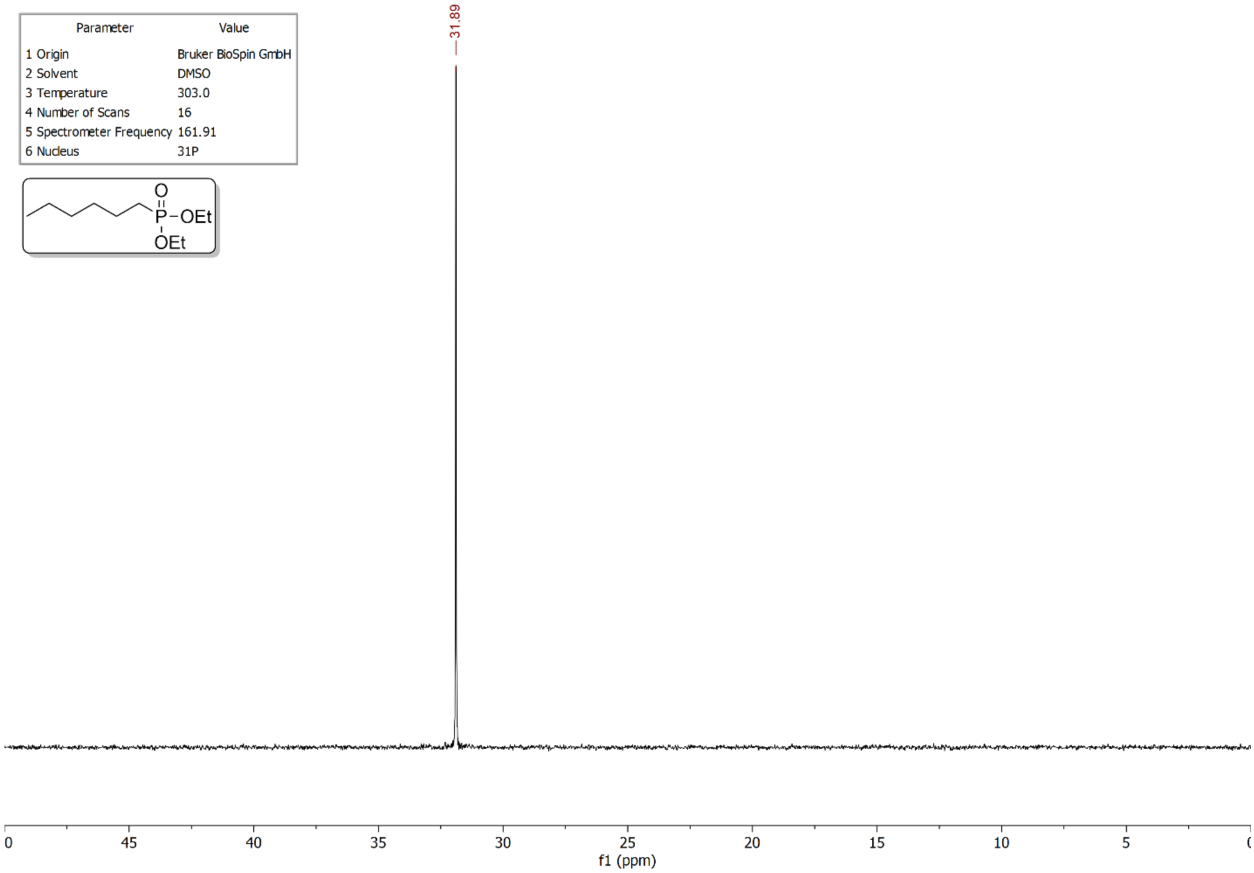

Parameter Value
1 Origin Bruker BioSpin GmbH
2 Solvent DMSO
3 Temperature 303.0
4 Number of Scans 16
5 Spectrometer Frequency 161.91
6 Nucleus 31P
O
P–OEt
OEt
31.89
f1 (ppm)

Diethyl dodecylphosphonate (**4f**)

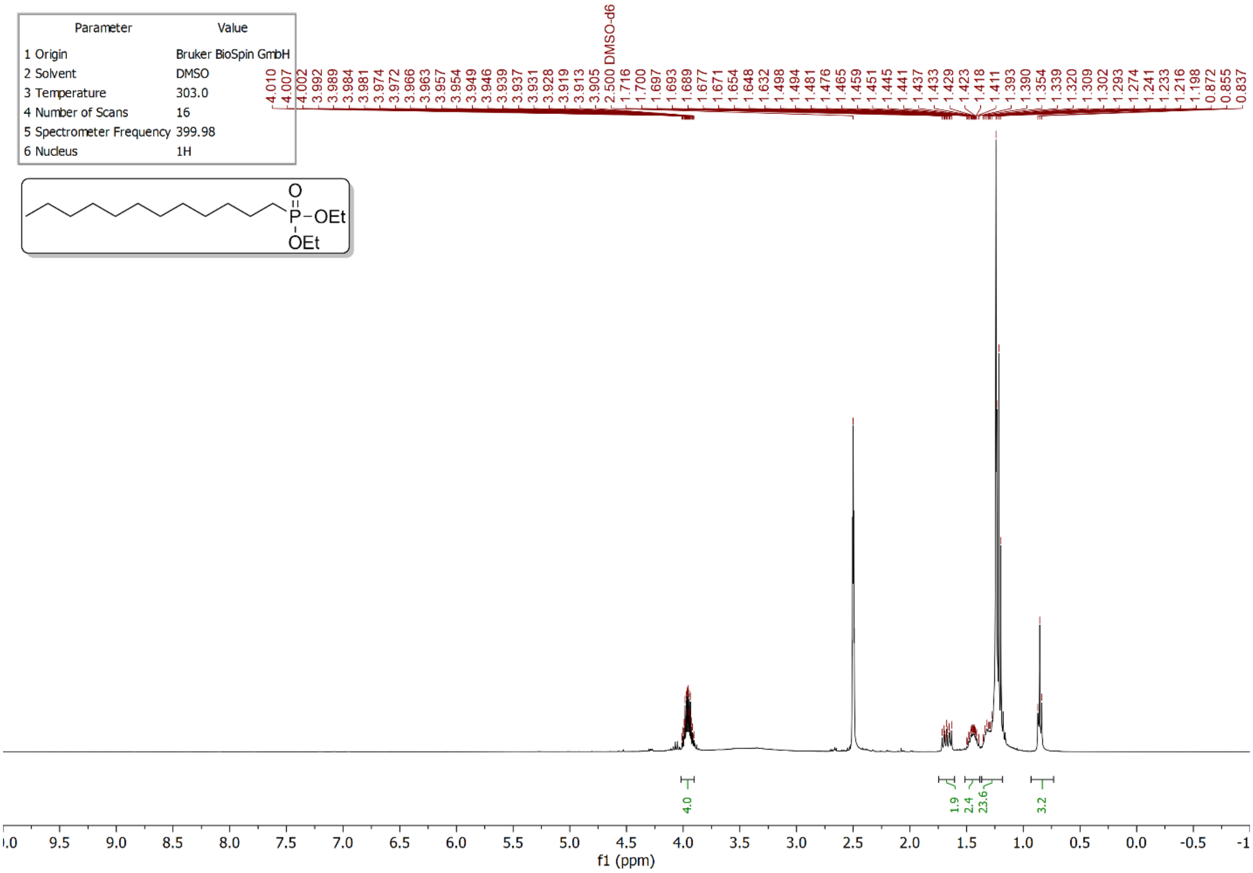

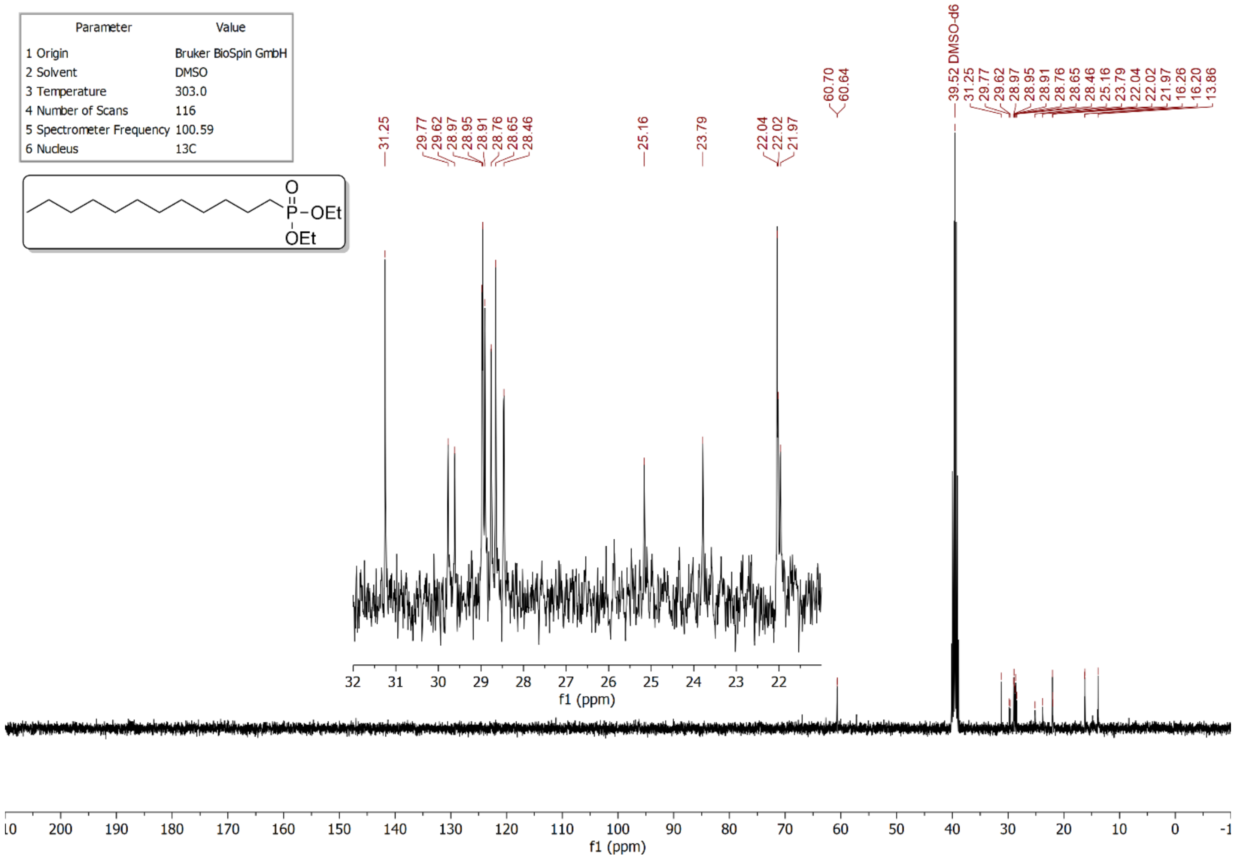

Parameter Value
1 Origin Bruker BioSpin GmbH
2 Solvent DMSO
3 Temperature 303.0
4 Number of Scans 116
5 Spectrometer Frequency 100.59
6 Nucleus 13C
P-OEt
OEt
39.52 DMSO-d6
f1 (ppm)

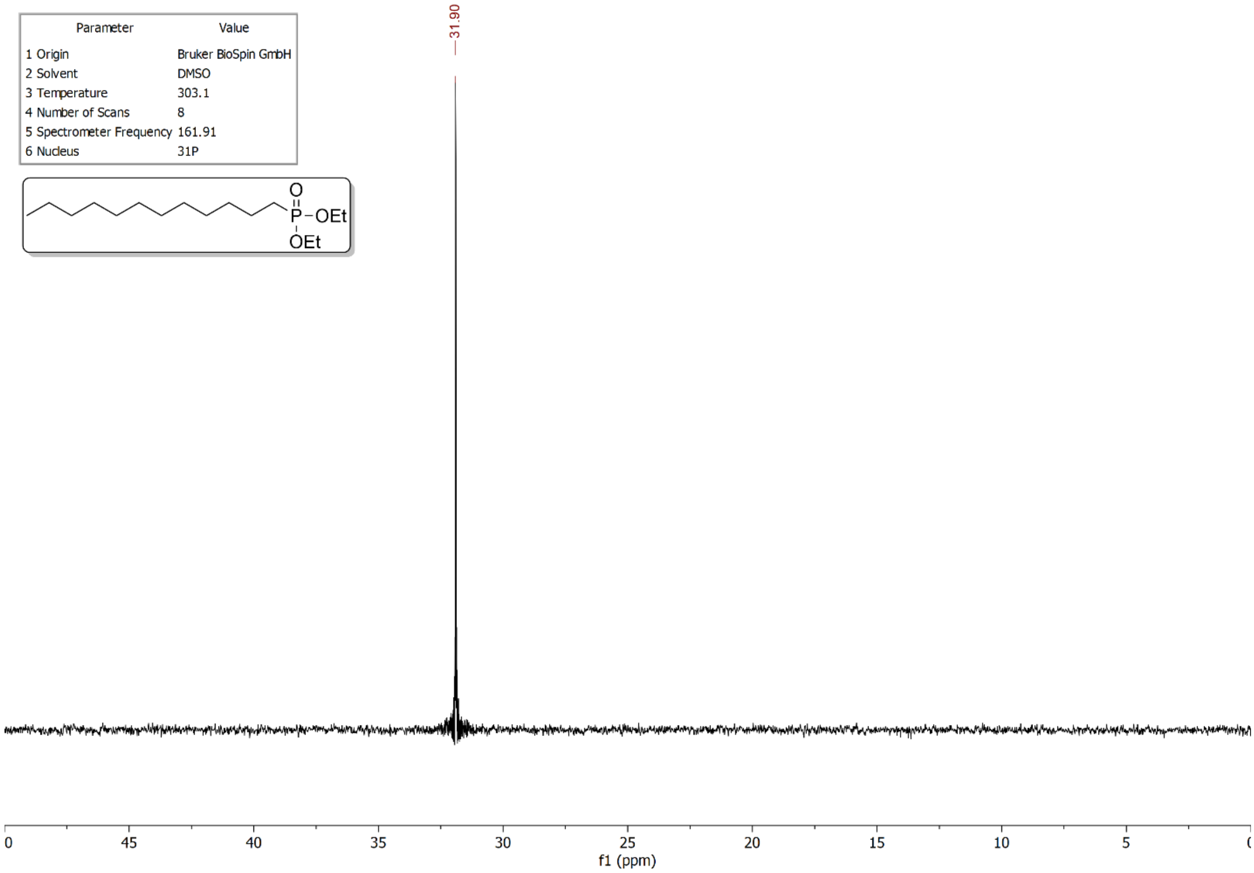

Parameter Value
1 Origin Bruker BioSpin GmbH
2 Solvent DMSO
3 Temperature 303.1
4 Number of Scans 8
5 Spectrometer Frequency 161.91
6 Nucleus 31P
P-OEt
OEt
31.90
f1 (ppm)

Diethyl phenylphosphonate (**4g**)

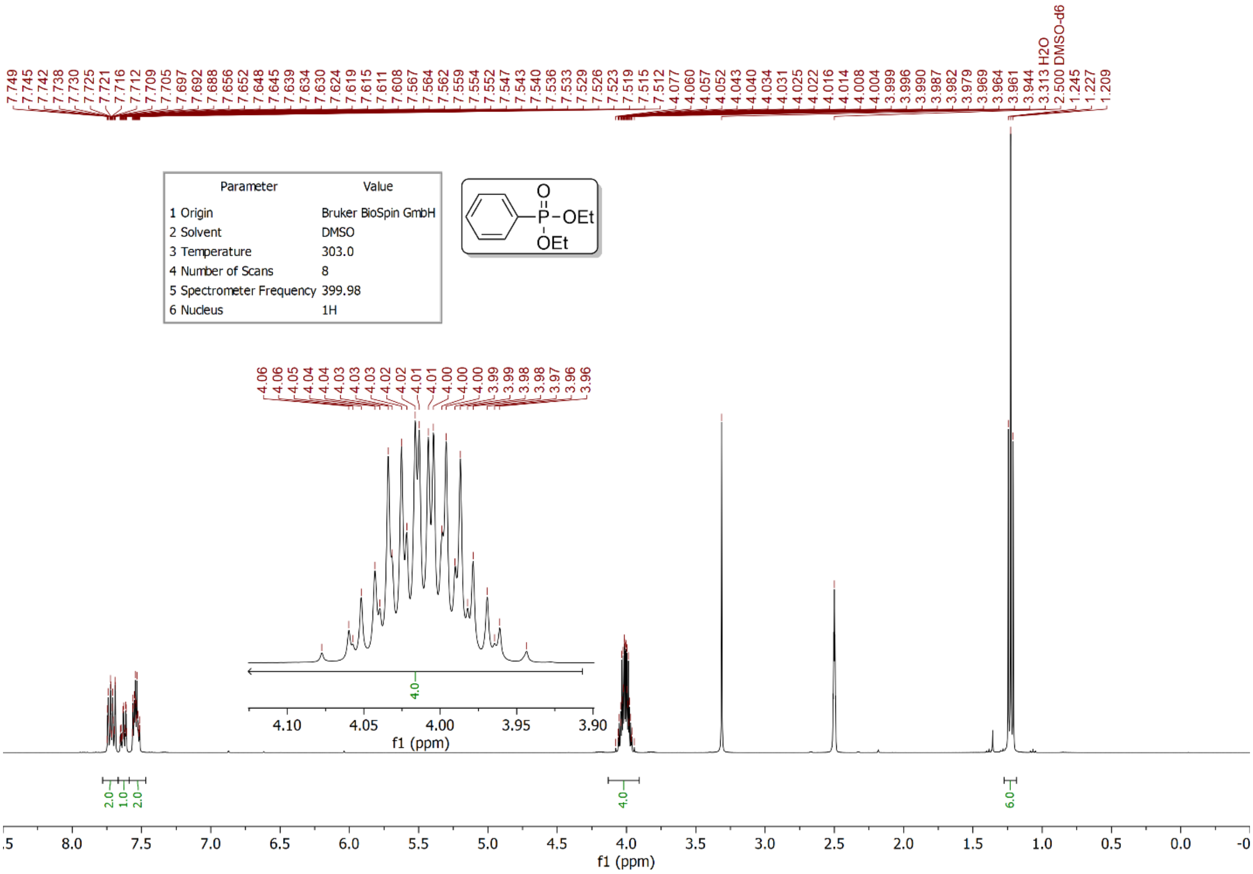

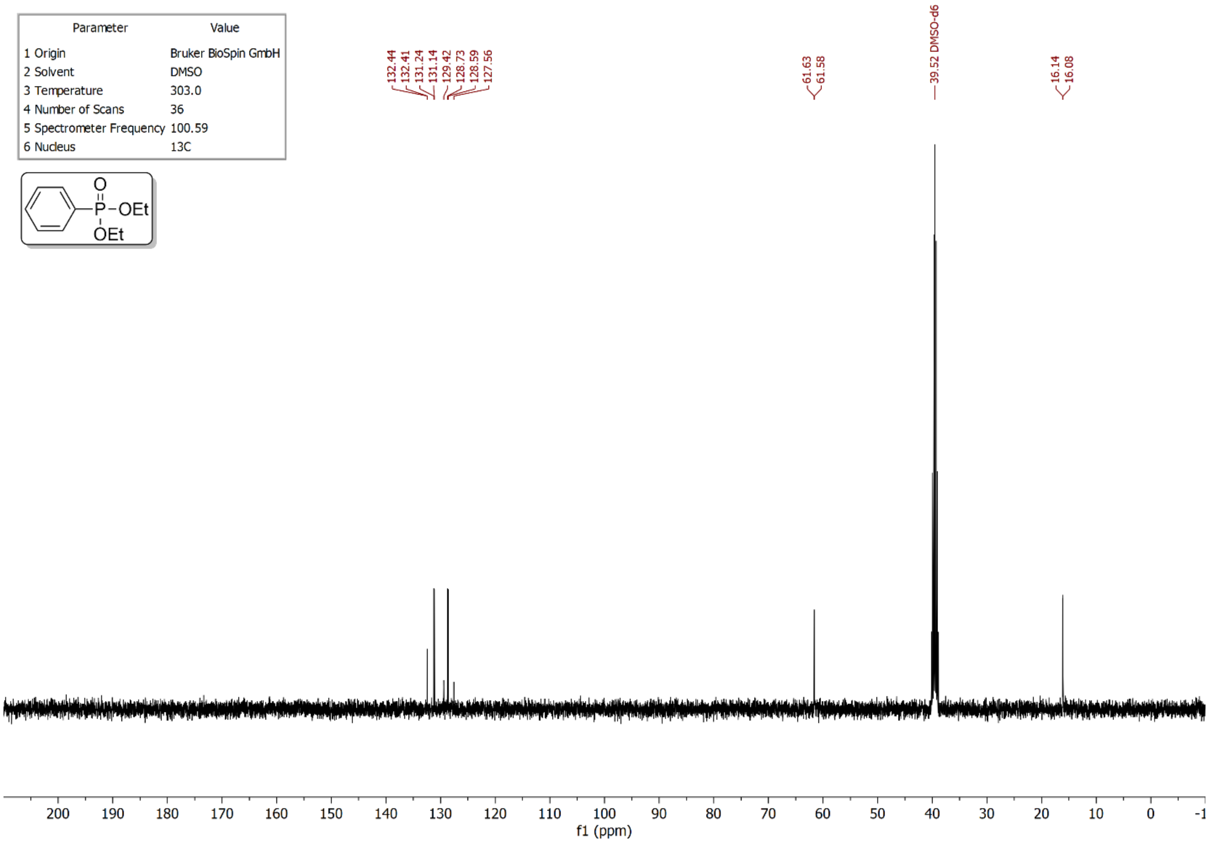
Parameter Value
1 Origin Bruker BioSpin GmbH
2 Solvent DMSO
3 Temperature 303.0
4 Number of Scans 36
5 Spectrometer Frequency 100.59
6 Nucleus 13C
O
P–OEt
OEt
132.44
132.41
131.24
131.14
129.42
128.73
128.59
127.56
61.63
61.58
39.52 DMSO-d6
16.14
16.08
f1 (ppm)

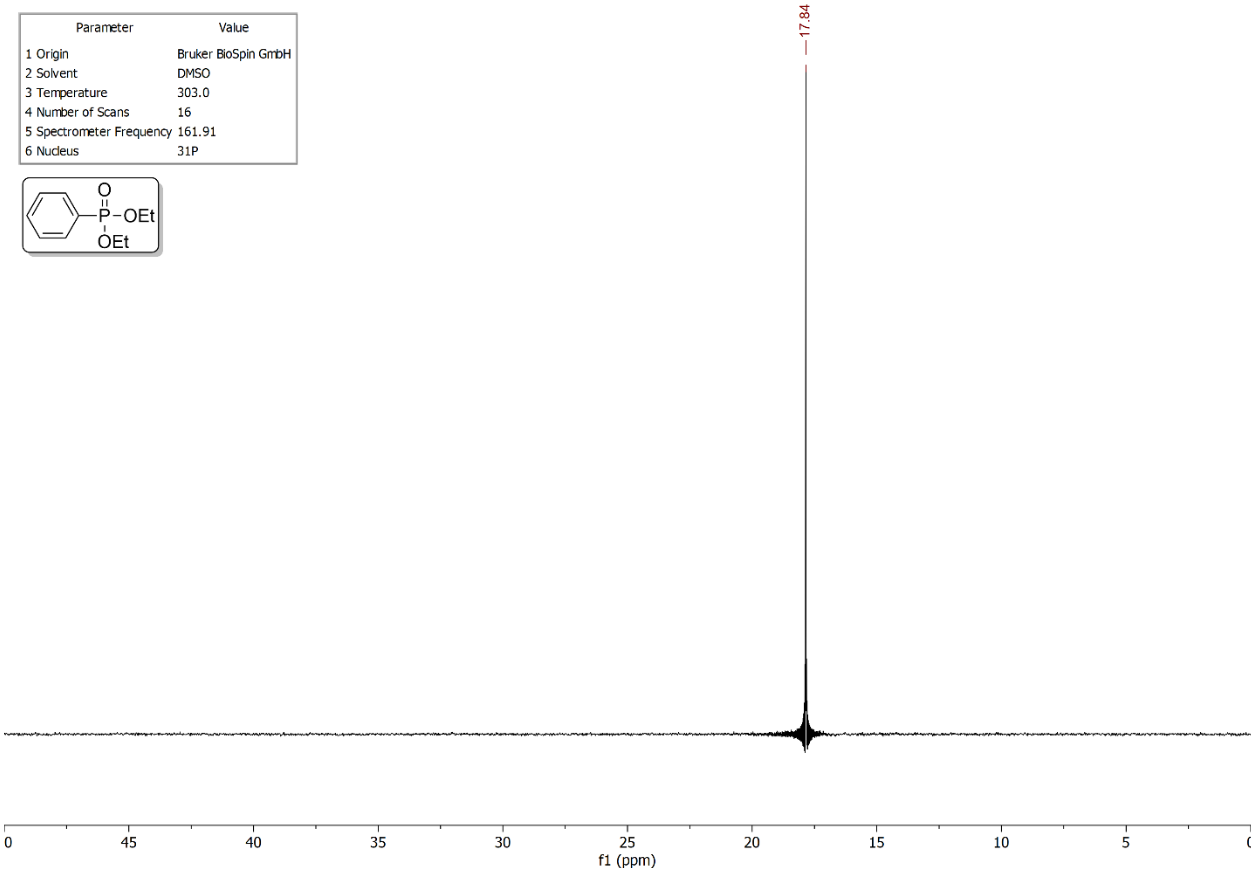
Parameter Value
1 Origin Bruker BioSpin GmbH
2 Solvent DMSO
3 Temperature 303.0
4 Number of Scans 16
5 Spectrometer Frequency 161.91
6 Nucleus 31P
O
P–OEt
OEt
17.84
f1 (ppm)

Diethyl (4-methoxyphenyl)phosphonate (**4h**)

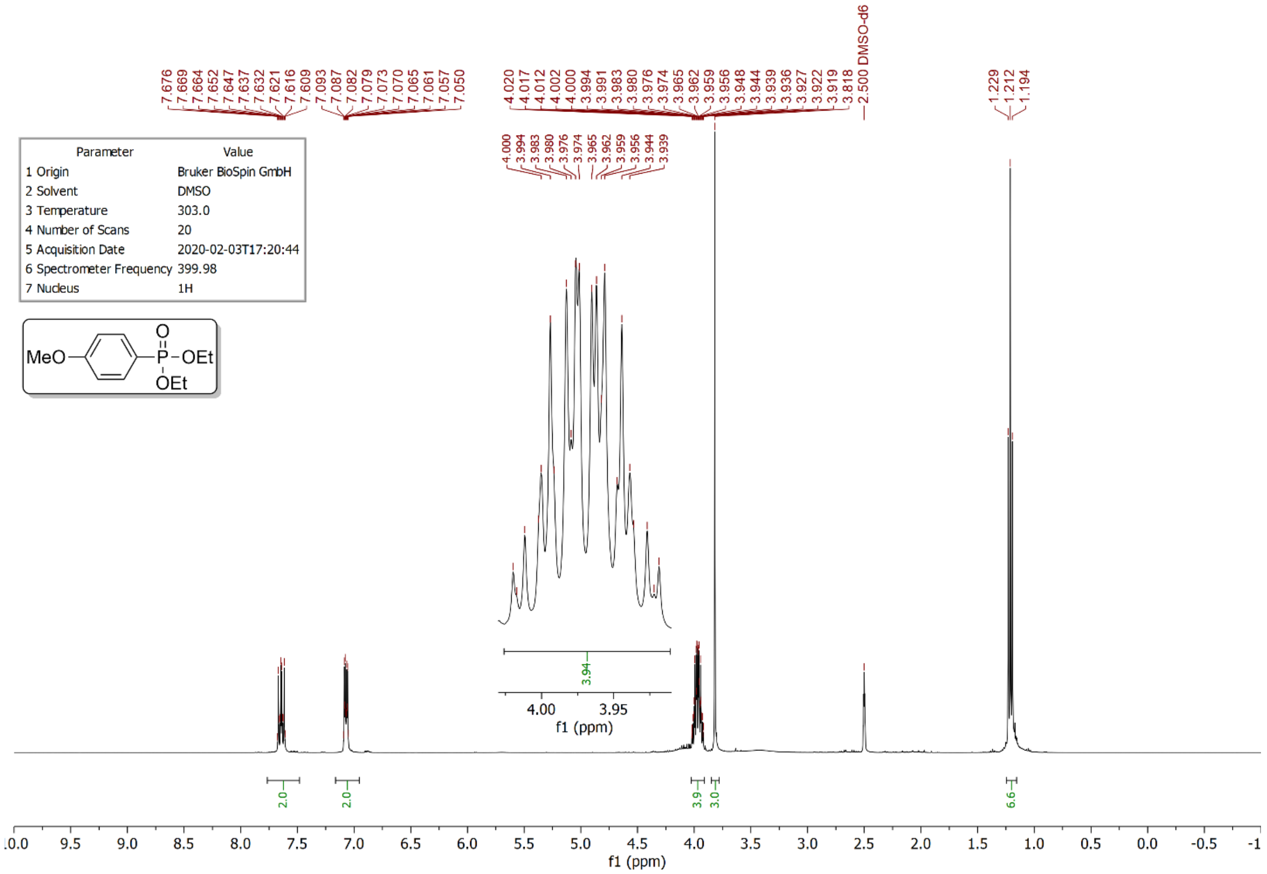

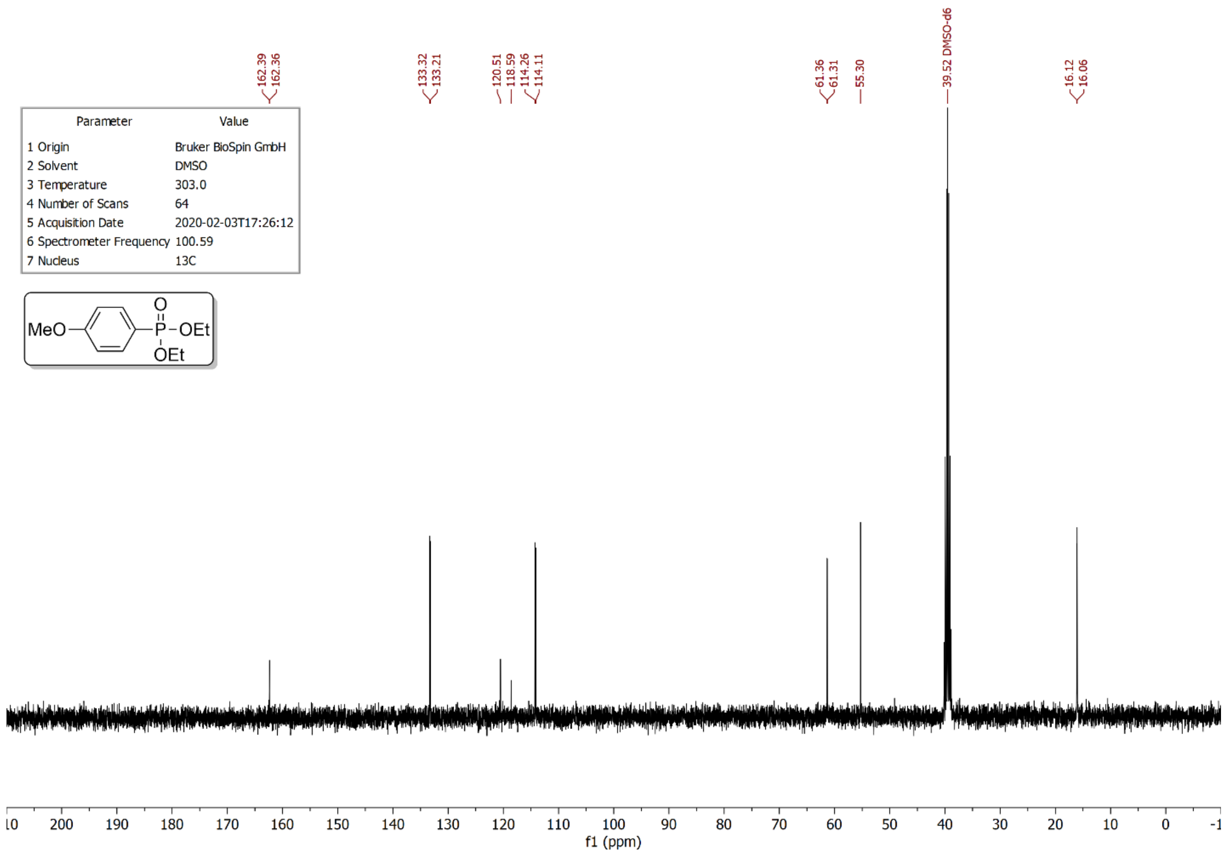
162.39
162.36
133.32
133.21
120.51
118.59
114.26
114.11
61.36
61.31
55.30
39.52 DMSO-d6
16.12
16.06
Parameter Value
1 Origin Bruker BioSpin GmbH
2 Solvent DMSO
3 Temperature 303.0
4 Number of Scans 64
5 Acquisition Date 2020-02-03T17:26:12
6 Spectrometer Frequency 100.59
7 Nucleus 13C
MeO
O
P
OEt
OEt
f1 (ppm)

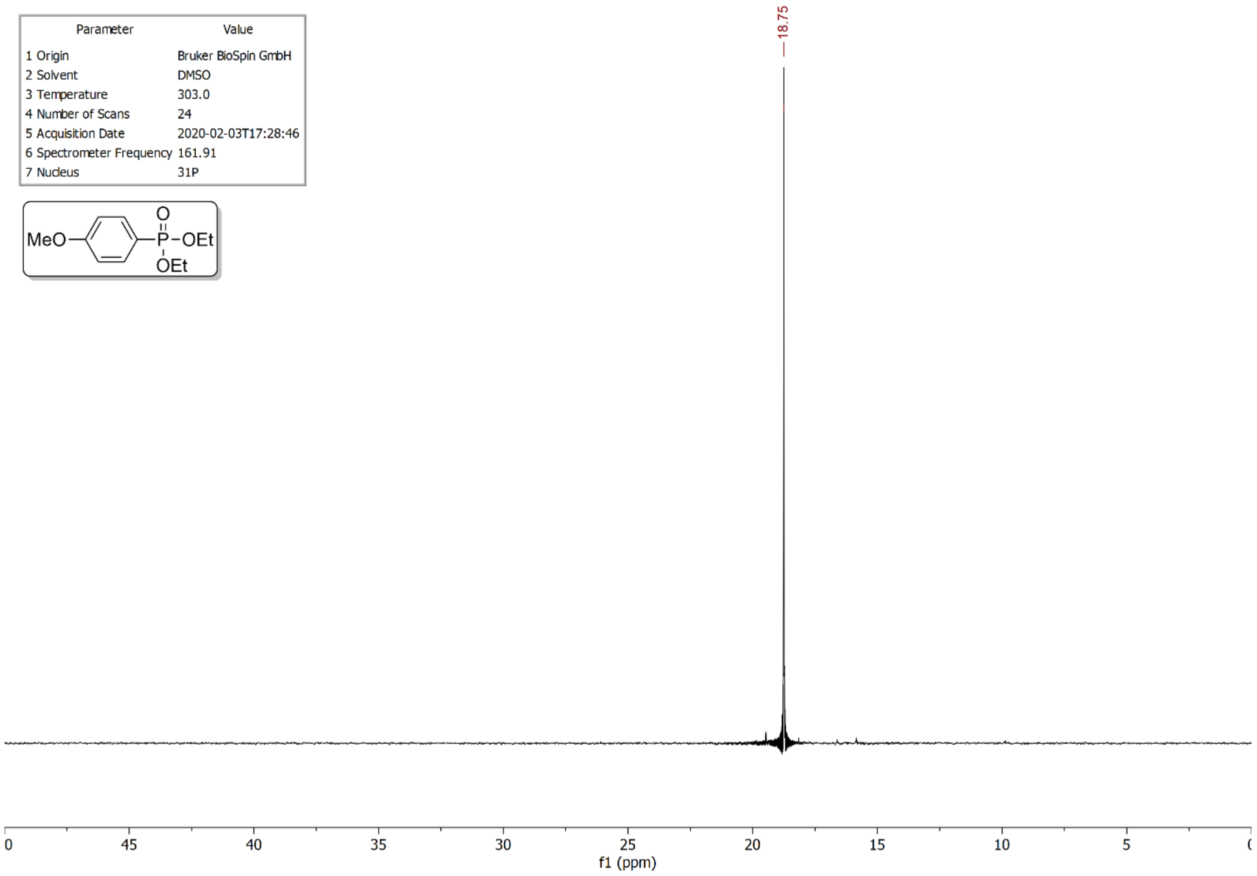
18.75
Parameter Value
1 Origin Bruker BioSpin GmbH
2 Solvent DMSO
3 Temperature 303.0
4 Number of Scans 24
5 Acquisition Date 2020-02-03T17:28:46
6 Spectrometer Frequency 161.91
7 Nucleus 31P
MeO
O
P
OEt
OEt
f1 (ppm)

Diethyl [(4-hydroxyphenyl)methyl]phosphonate (**4i**)

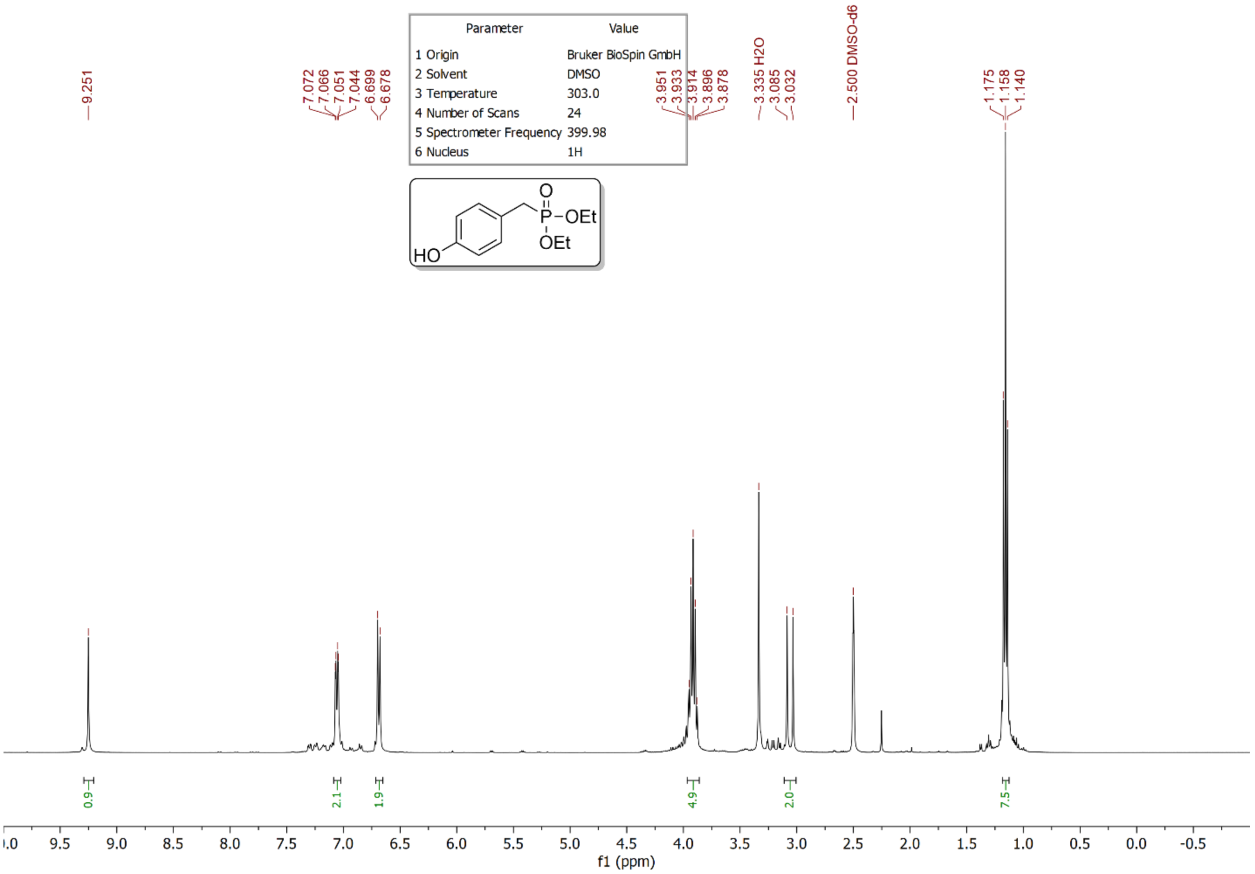

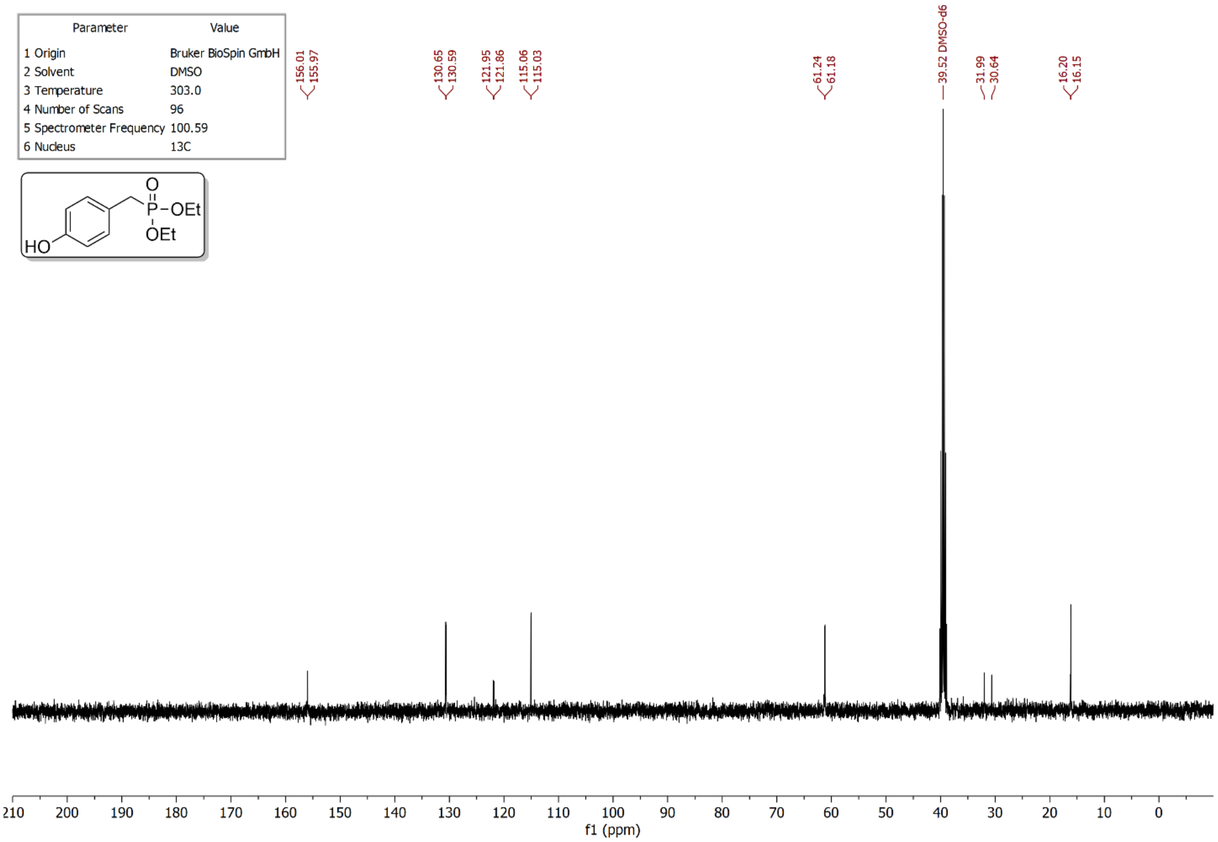
Parameter Value
1 Origin Bruker BioSpin GmbH
2 Solvent DMSO
3 Temperature 303.0
4 Number of Scans 96
5 Spectrometer Frequency 100.59
6 Nucleus 13C
HO
O
P–OEt
OEt
156.01
155.97
130.65
130.59
121.95
121.86
115.06
115.03
61.24
61.18
39.52 DMSO-d6
31.99
30.64
16.20
16.15
f1 (ppm)

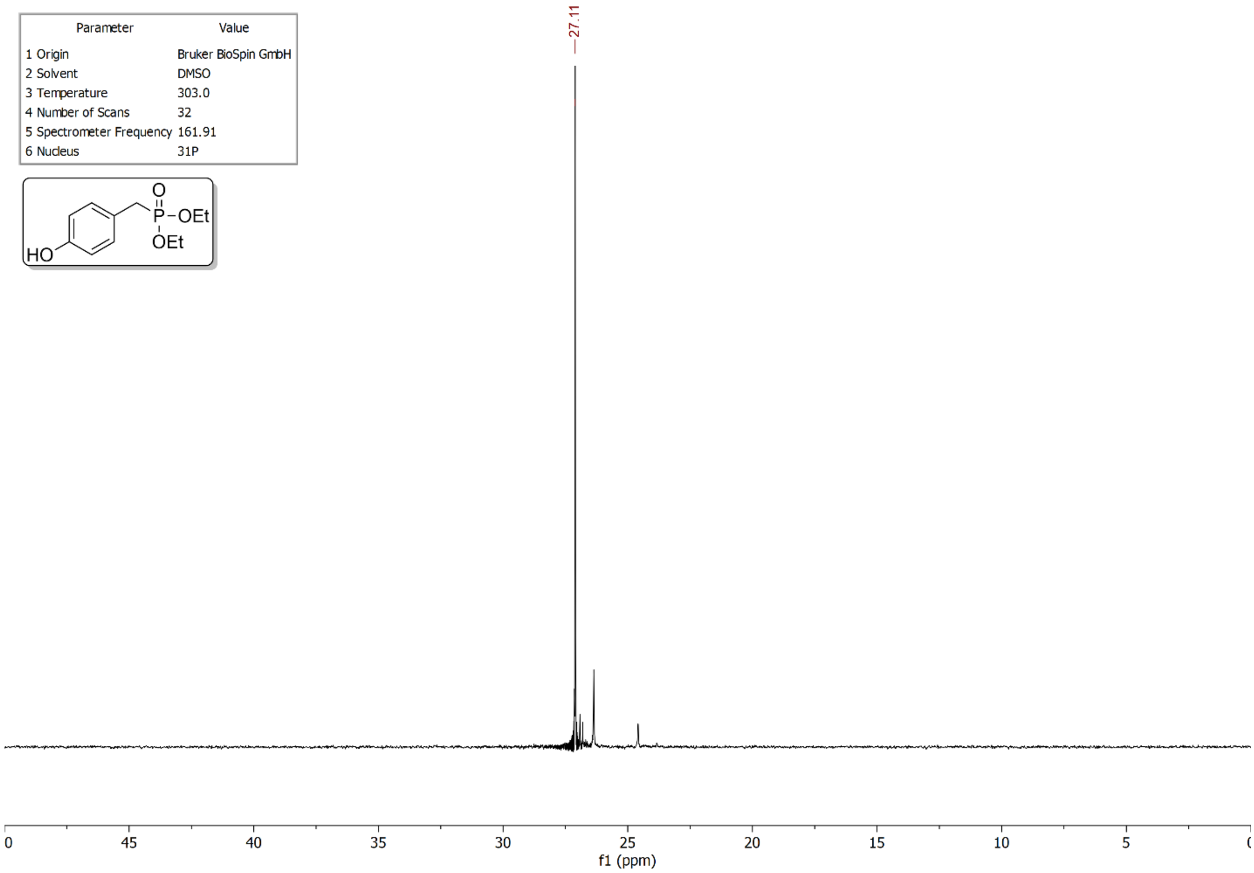
Parameter Value
1 Origin Bruker BioSpin GmbH
2 Solvent DMSO
3 Temperature 303.0
4 Number of Scans 32
5 Spectrometer Frequency 161.91
6 Nucleus 31P
HO
O
P–OEt
OEt
27.11
f1 (ppm)

Diethyl [(4-nitrophenyl)methyl]phosphonate (**4j**)

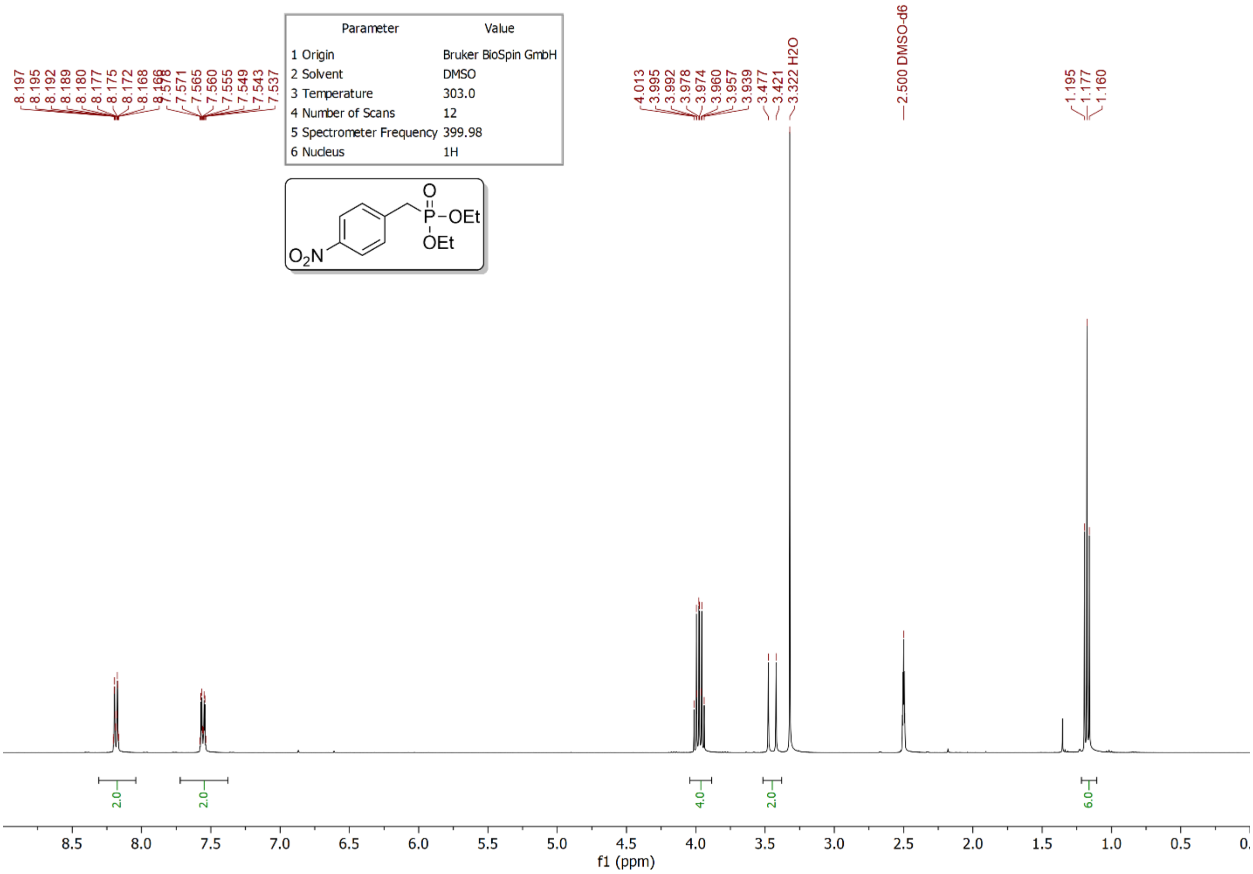

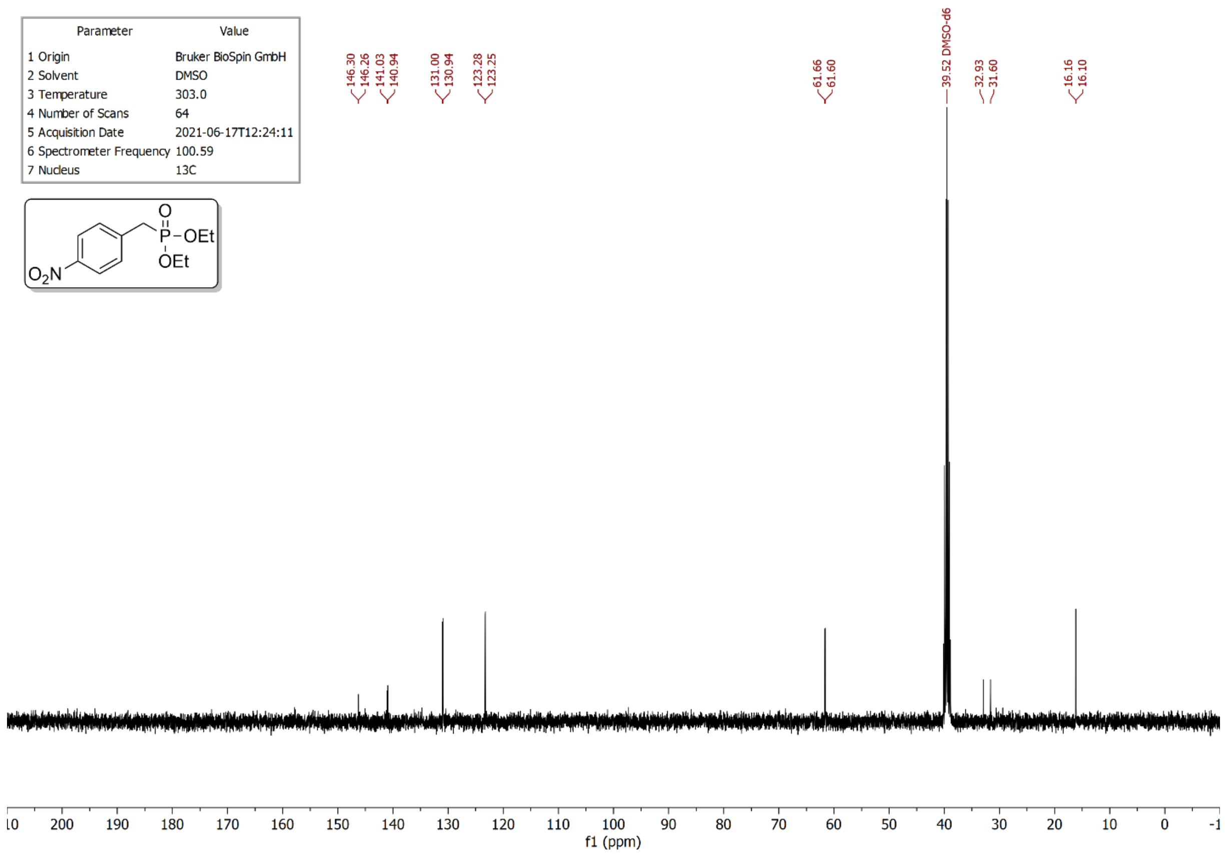
Parameter Value
1 Origin Bruker BioSpin GmbH
2 Solvent DMSO
3 Temperature 303.0
4 Number of Scans 64
5 Acquisition Date 2021-06-17T12:24:11
6 Spectrometer Frequency 100.59
7 Nucleus 13C
146.30
146.26
141.03
140.94
131.00
130.94
123.28
123.25
61.66
61.60
39.52 DMSO-d6
32.93
31.60
16.16
16.10
f1 (ppm)

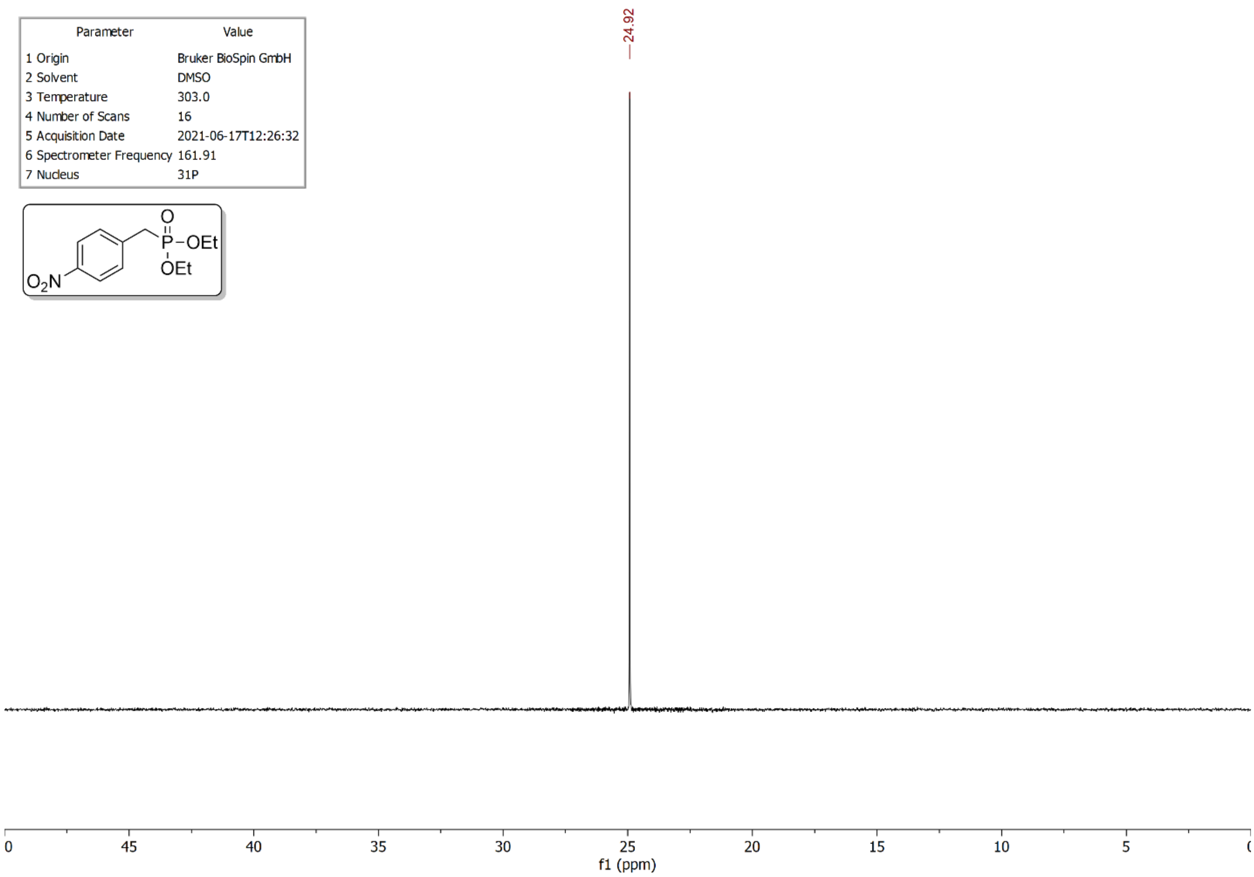
Parameter Value
1 Origin Bruker BioSpin GmbH
2 Solvent DMSO
3 Temperature 303.0
4 Number of Scans 16
5 Acquisition Date 2021-06-17T12:26:32
6 Spectrometer Frequency 161.91
7 Nucleus 31P
24.92
f1 (ppm)

Diethyl [(4-bromophenyl)methyl]phosphonate (**4k**)

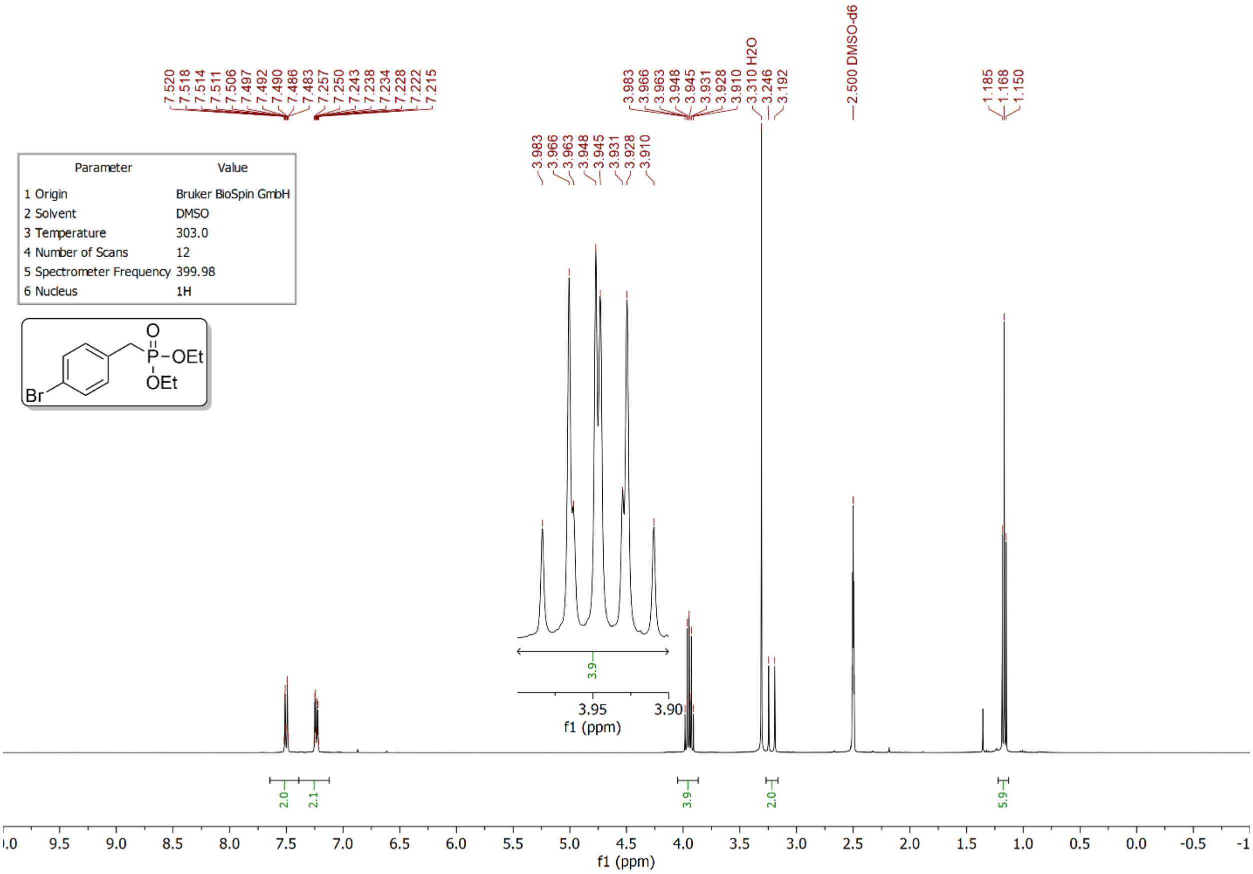

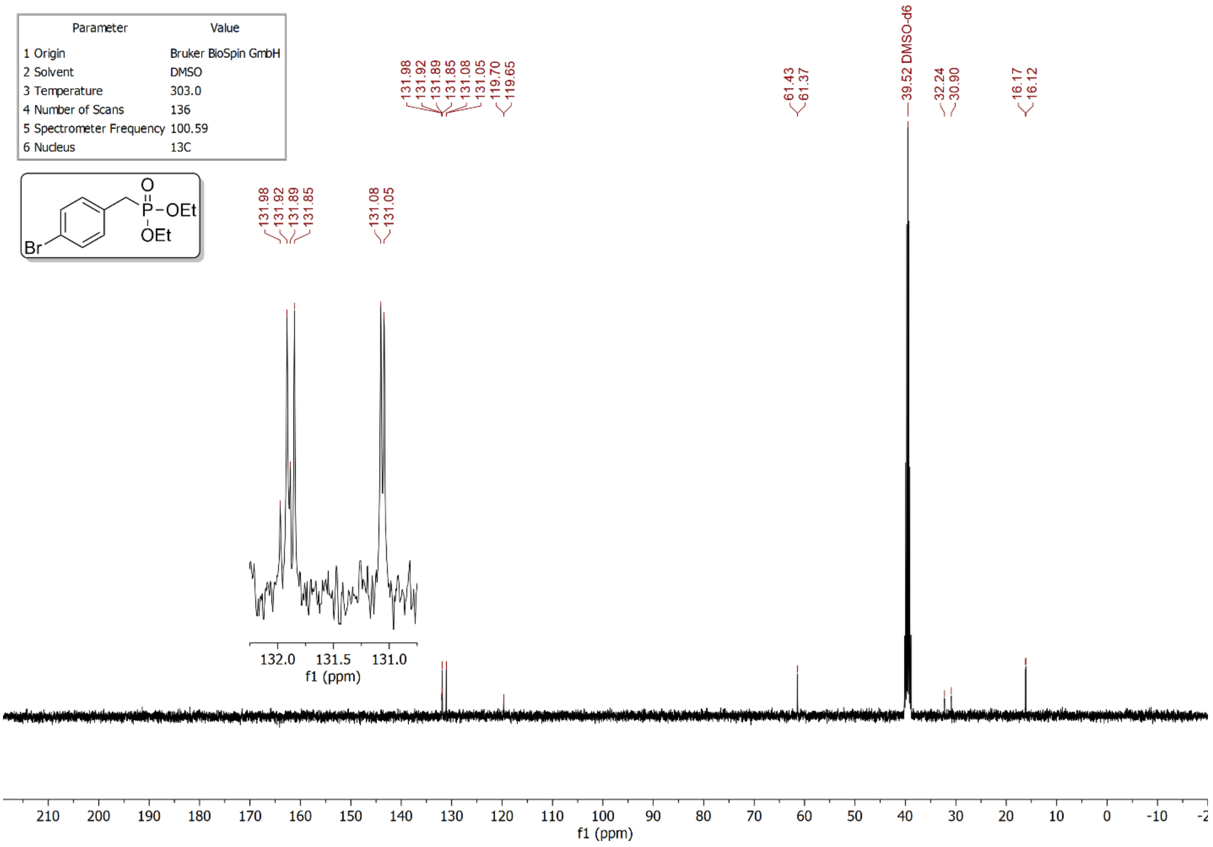
Parameter Value
1 Origin Bruker BioSpin GmbH
2 Solvent DMSO
3 Temperature 303.0
4 Number of Scans 136
5 Spectrometer Frequency 100.59
6 Nucleus 13C
P-OEt
OEt
Br
131.98
131.92
131.89
131.85
131.08
131.05
119.70
119.65
61.43
61.37
39.52 DMSO-d6
32.24
30.90
16.17
16.12
f1 (ppm)

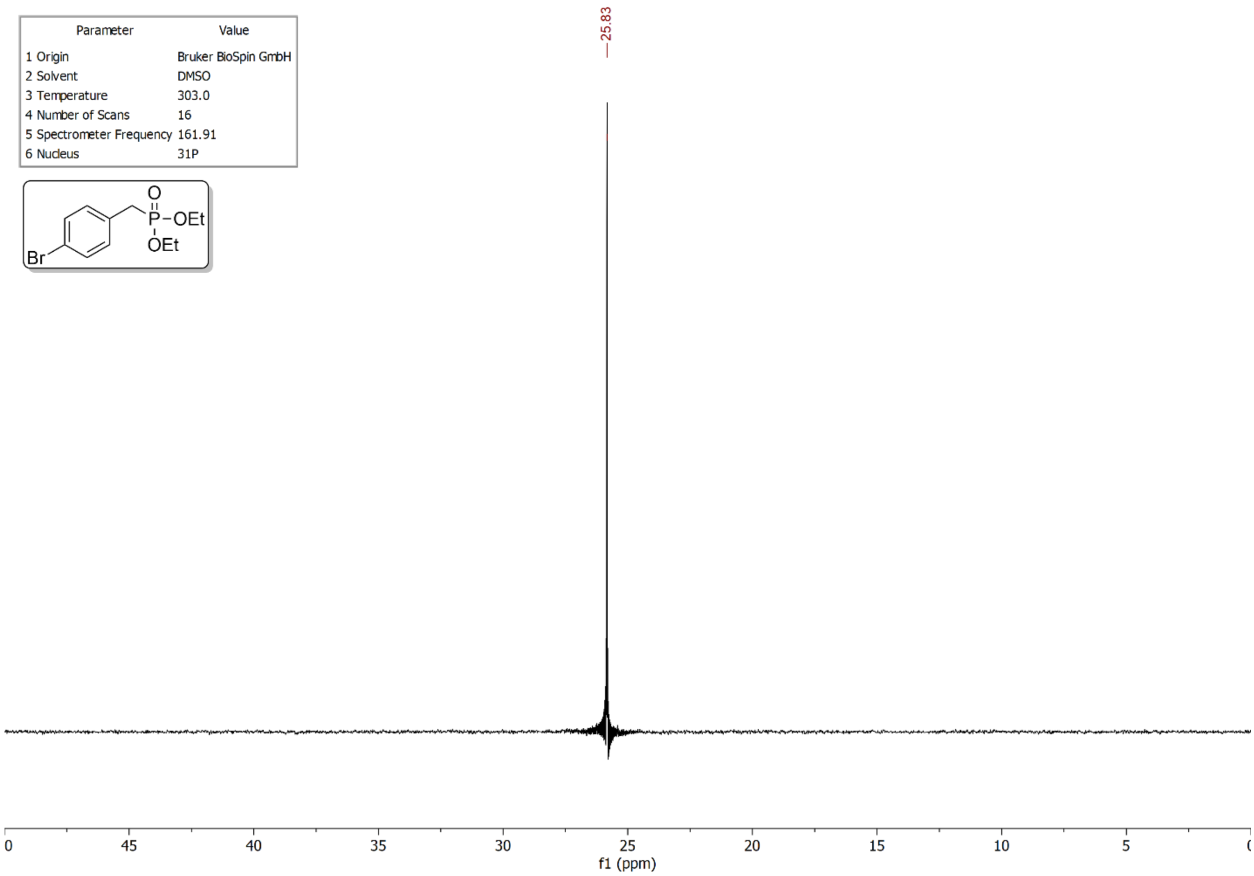
Parameter Value
1 Origin Bruker BioSpin GmbH
2 Solvent DMSO
3 Temperature 303.0
4 Number of Scans 16
5 Spectrometer Frequency 161.91
6 Nucleus 31P
P-OEt
OEt
Br
25.83
f1 (ppm)

Diethyl [(3-bromophenyl)methyl]phosphonate (**4l**)

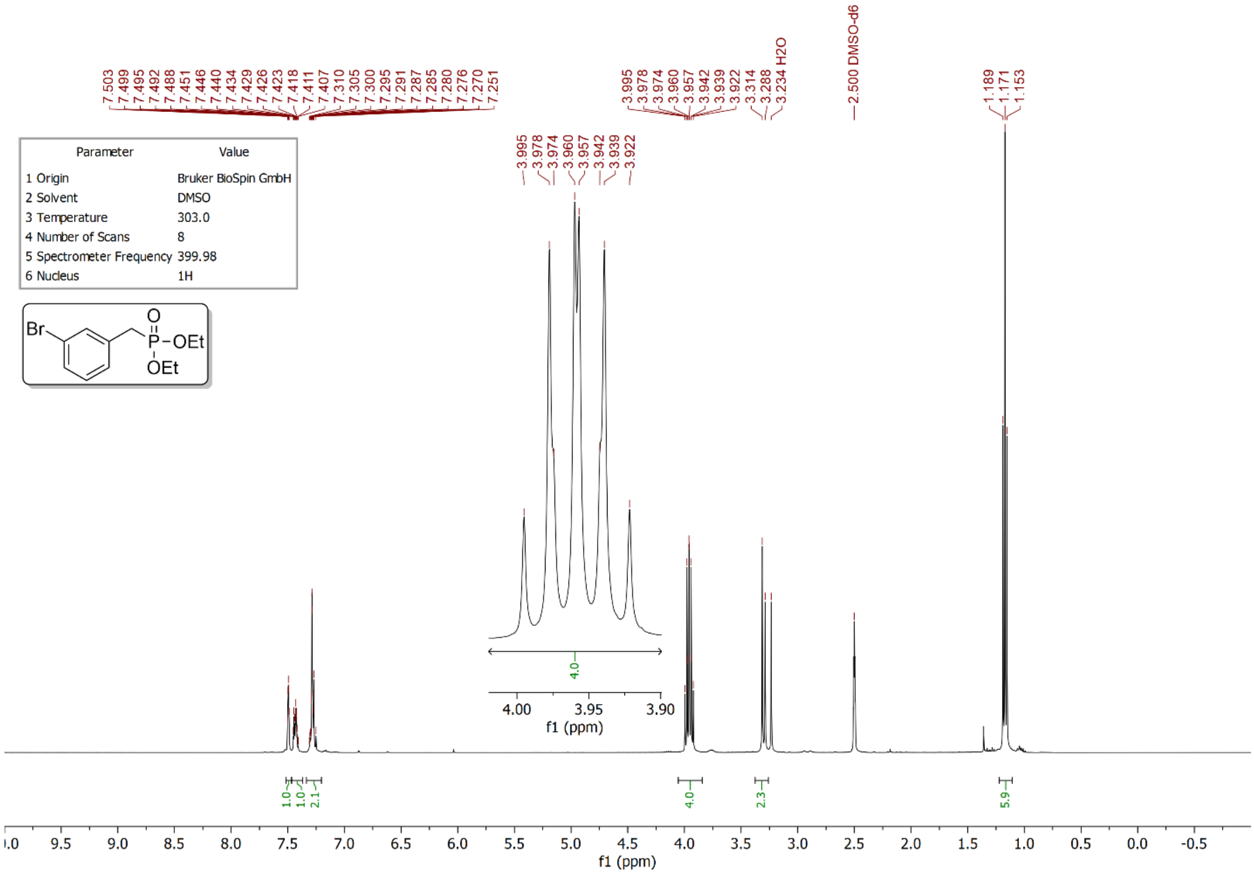

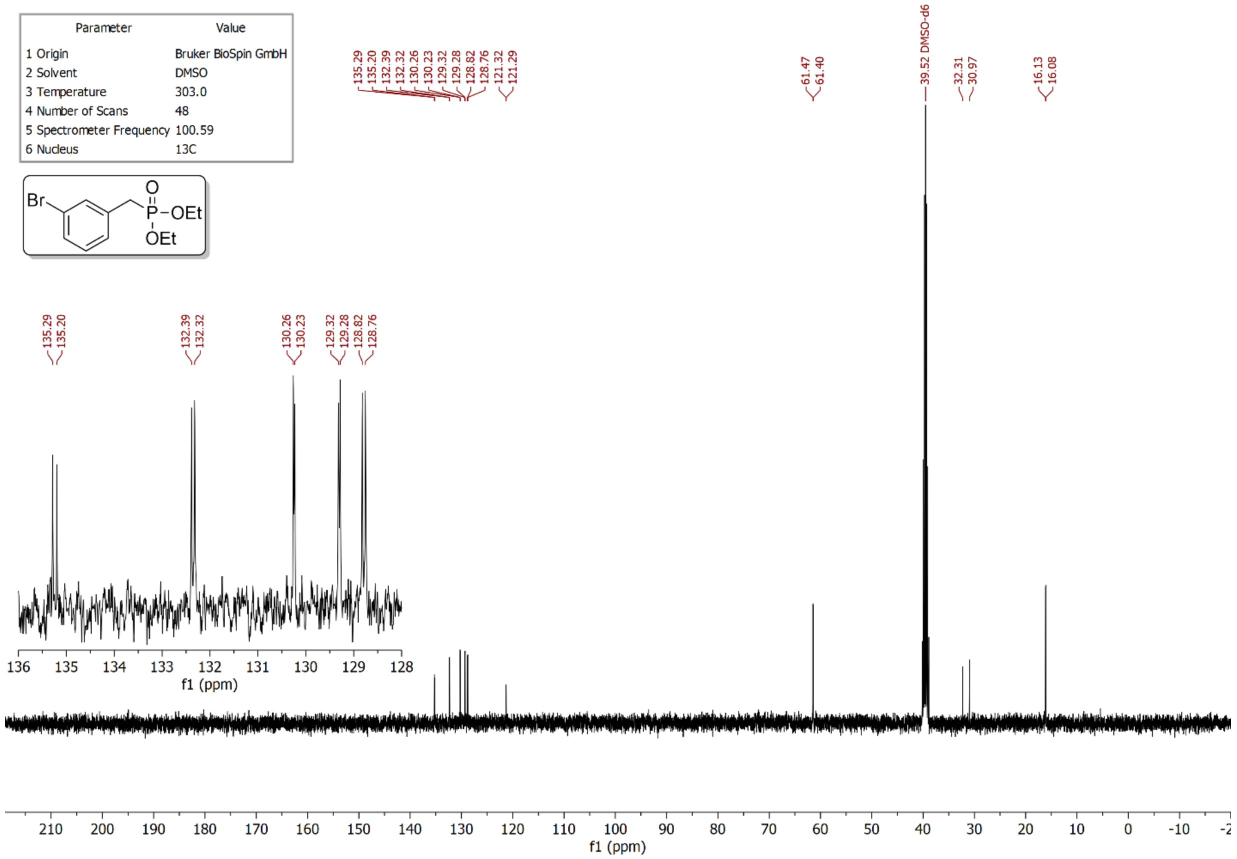
Parameter Value
1 Origin Bruker BioSpin GmbH
2 Solvent DMSO
3 Temperature 303.0
4 Number of Scans 48
5 Spectrometer Frequency 100.59
6 Nucleus 13C
Br
O
P–OEt
OEt
135.29
135.20
132.39
132.32
130.26
130.23
129.32
129.28
128.82
128.76
121.32
121.29
61.47
61.40
39.52 DMSO-d6
32.31
30.97
16.13
16.08
f1 (ppm)

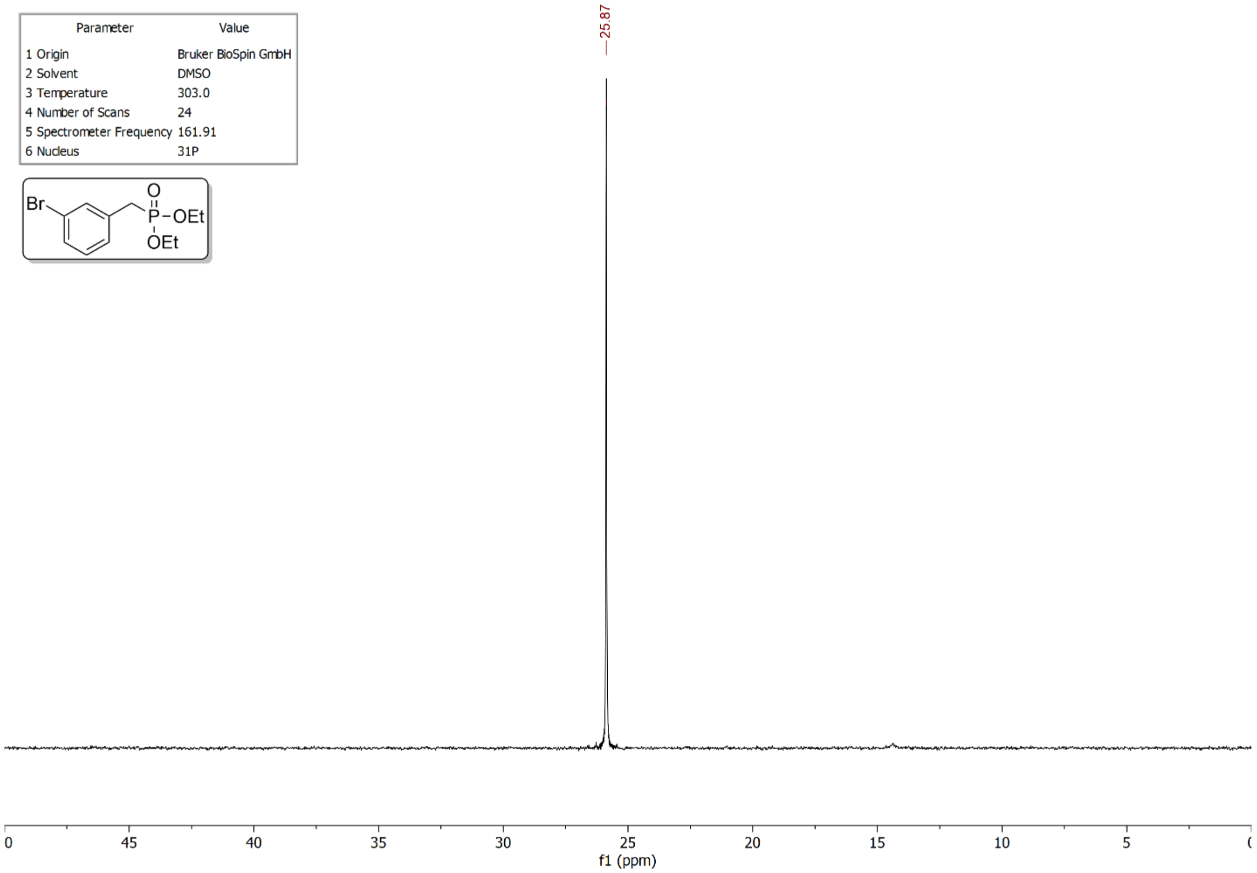
Parameter Value
1 Origin Bruker BioSpin GmbH
2 Solvent DMSO
3 Temperature 303.0
4 Number of Scans 24
5 Spectrometer Frequency 161.91
6 Nucleus 31P
Br
O
P–OEt
OEt
25.87
f1 (ppm)
